# HIV virologic failure and its predictors among HIV-infected adults on antiretroviral therapy in the African Cohort Study

**DOI:** 10.1371/journal.pone.0211344

**Published:** 2019-02-05

**Authors:** Francis Kiweewa, Allahna Esber, Ezra Musingye, Domonique Reed, Trevor A. Crowell, Fatim Cham, Michael Semwogerere, Rosemary Namagembe, Alice Nambuya, Cate Kafeero, Allan Tindikahwa, Leigh Anne Eller, Monica Millard, Huub C. Gelderblom, Babajide Keshinro, Yakubu Adamu, Jonah Maswai, John Owuoth, Valentine Chepkorir Sing’oei, Lucas Maganga, Emmanuel Bahemana, Samoel Khamadi, Merlin L. Robb, Julie A. Ake, Christina S. Polyak, Hannah Kibuuka

**Affiliations:** 1 Makerere University- Walter Reed Project, Kampala, Uganda; 2 U.S. Military HIV Research Program, Walter Reed Army Institute of Research, Silver Spring, Maryland, United States of America; 3 Henry M. Jackson Foundation for the Advancement of Military Medicine, Bethesda, United States of America; 4 International AIDS Vaccine Initiative, New York, New York, United States of America; 5 HJF Medical Research International, Abuja, Nigeria; 6 HJF Medical Research International, Kericho, Kenya; 7 HJF Medical Research International, Kisumu, Kenya; 8 Mbeya Medical Research Centre, Mbeya, Tanzania; 9 HJF Medical Research International, Mbeya, Tanzania; Consejo Superior de Investigaciones Cientificas, SPAIN

## Abstract

**Introduction:**

The 2016 WHO consolidated guidelines on the use of antiretroviral drugs defines HIV virologic failure for low and middle income countries (LMIC) as plasma HIV-RNA ≥ 1000 copies/mL. We evaluated virologic failure and predictors in four African countries.

**Materials and methods:**

We included HIV-infected participants on a WHO recommended antiretroviral therapy (ART) regimen and enrolled in the African Cohort Study between January 2013 and October 2017. Studied outcomes were virologic failure (plasma HIV-RNA ≥ 1000 copies/mL at the most recent visit), viraemia (plasma HIV-RNA ≥ 50 copies/mL at the most recent visit); and persistent viraemia (plasma HIV-RNA ≥ 50 copies/mL at two consecutive visits). Generalized linear models were used to estimate relative risks with their 95% confidence intervals.

**Results:**

2054 participants were included in this analysis. Viraemia, persistent viraemia and virologic failure were observed in 396 (19.3%), 160 (7.8%) and 184 (9%) participants respectively. Of the participants with persistent viraemia, only 57.5% (92/160) had confirmed virologic failure. In the multivariate analysis, attending clinical care site other than the Uganda sitebeing on 2^nd^ line ART (aRR 1.8, 95% CI 1·28–2·66); other ART combinations not first line and not second line (aRR 3.8, 95% CI 1.18–11.9), a history of fever in the past week (aRR 3.7, 95% CI 1.69–8.05), low CD4 count (aRR 6.9, 95% CI 4.7–10.2) and missing any day of ART (aRR 1·8, 95% CI 1·27–2.57) increased the risk of virologic failure. Being on 2^nd^ line therapy, the site where one receives care and CD4 count < 500 predicted viraemia, persistent viraemia and virologic failure.

**Conclusion:**

In conclusion, these findings demonstrate that HIV-infected patients established on ART for more than six months in the African setting frequently experienced viraemia while continuing to be on ART. The findings also show that being on second line, low CD4 count, missing any day of ART and history of fever in the past week remain important predictors of virologic failure that should trigger intensified adherence counselling especially in the absence of reliable or readily available viral load monitoring. Finally, clinical care sites are different calling for further analyses to elucidate on the unique features of these sites.

## Introduction

The goal of antiretroviral therapy (ART) for HIV infection is to achieve and maintain virologic suppression, thereby preventing disease progression and transmission. In 2014, the Joint United Nations Programme on HIV/AIDS (UNAIDS) set the 90-90-90 global targets for elimination of HIV, whereby the third 90 represents a target to achieve viral suppression in at least 90% of patients initiating ART by 2020 [1], Abrams and Strasser [2]. The 2016 WHO consolidated antiretroviral guidelines define virologic suppression for a public health approach as HIV RNA < 1000 copies/mL [3]. By the end of 2016, at least 19 million people living with HIV globally had initiated ART [4]. Of these, 72% were living in sub-Saharan Africa [5]. The public health impact of this achievement will depend on the extent to which those initiating ART are able to achieve and maintain virologic suppression [6].

Progress towards the 90-90-90 targets in Sub-Saharan Africa is hampered by reports from the region that viraemia and virologic failure are common (11% to 24%), with 71% to 90% of the latter having HIV drug resistance mutations [7–11]. Identifying factors contributing to viraemia and virologic failure is key to achieving this target.

Previously published research findings highlight a number of factors that may be associated with viraemia, including WHO stage, clinician skill level, age, CD4 count, treatment history, and suboptimal adherence [12–15]. However, many of the studies on the factors associated with viraemia are in high income countries that use a lower threshold to determine viraemia [16]. In contrast, studies from the majority of low and middle income countries use the WHO public health approach based thresholds for determining viraemia [17]. Additionally, some of the published studies on HIV viral suppression or viraemia have been limited by small sample size [18, 19]. The relative role of various factors associated with viraemia may also be dependent on the ART program setting and the local context. Therefore, a comparison of viraemia and predictors across different contexts, and a comparison of the different definitions is key to informing adjustments in program level strategies. We evaluated the prevalence of virologic failure, viraemia and the associated factors among adult male and female HIV-infected participants in four African countries.

## Materials and methods

### Study design and participants

**Fig 1:**
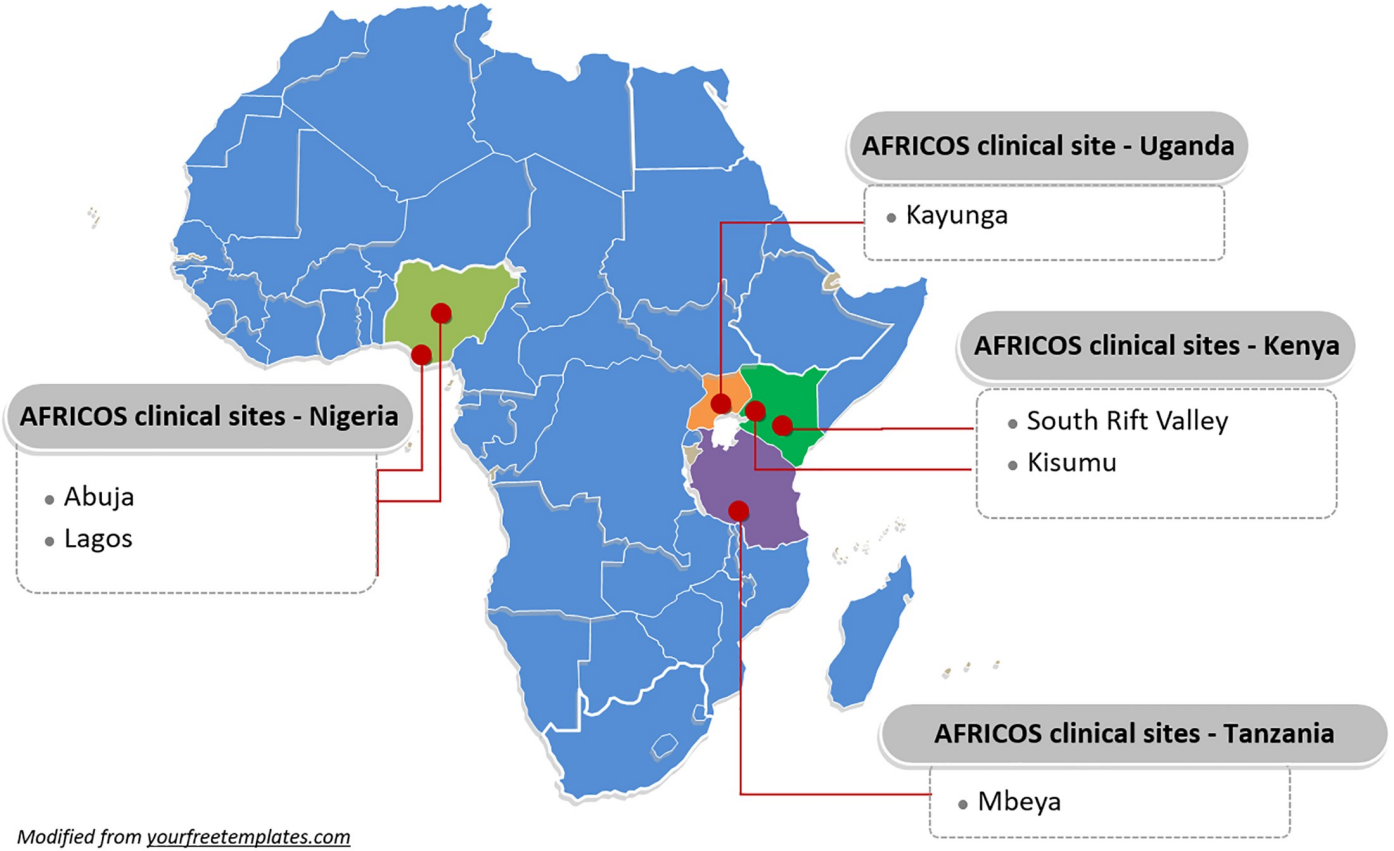
The African Cohort Study (AFRICOS) clinical sites.

The African Cohort Study (AFRICOS) is a prospective, multicenter cohort study following HIV-infected participants aged 18 years and older attending outpatient clinical care facilities supported by the U.S. Military HIV Research Program (MHRP) through the U.S. President’s Emergency Fund for AIDS Relief (PEPFAR) in East Africa and Nigeria (Fig 1). A detailed description of the AFRICOS objectives, methodology and procedures can be found in the AFRICOS protocol (https://www.hivresearch.org/sites/default/files/RV%20329%20Protocol%20v2.6%2021NOV2016%20%28clean%29.pdf) and has previously been described [20]. In brief, participants are evaluated at baseline and biannually thereafter. At each visit, participants were administered a medical history and physical exam, completed a broad demographic and behavioural questionnaire, extracted ART treatment history and other clinical outcomes from the medical records, and underwent phlebotomy. ART was started at provider discretion based on local guidelines in place at time of care.

### Laboratory assessment

Laboratory assessment at each visit included CD4 T-cell count, plasma HIV viral load, full heamogram, serum chemistry, serum cryptococcal antigen, and QuantiFERON for TB exposure. HIV RNA viral load was assessed every 6 months using several different platforms; Roche Cobas Ampliprep/Cobas TaqMan HIV-1 Test, v2.0 (linear range 20–10,0000,000 copies/mL), Roche High Pure/COBAS TaqMan HIV-1 Test v2·0 (linear range 34–10,000,000 copies/mL), Roche COBAS AmpliPrep/COBAS TaqMan 48 HIV-1 Test (linear range 48–10,000,000 copies/mL) or Abbott Real Time HIV-1 Viral Load assay (linear range 40–10,000,000 copies/mL). Adherence was assessed by self-report.

### Data collection and outcomes

Data were entered and verified into the ClinPlus Data Management system (DZS Software Solutions, Bound Brock, NJ). Participants’ data were eligible for inclusion in the analysis if they had been continuously taking ART for at least six months and had viral load data at the most recent visit. The 6 months cut off was informed by evidence that the majority of patients achieve viral suppression within 3–6 months of initiating ART [21–23] and the WHO recommendation to check for viral suppression after at least 6 months of treatment. The primary outcome was virologic failure, defined as plasma HIV RNA ≥ 1000 copies/mL at the most recent visit. As secondary outcomes we examined viraemia (HIV RNA >50 copies/mL) and persistent viraemia, defined as plasma HIV RNA ≥50 copies/mL at the two most recent visits six months apart. Exploratory variables considered in the analysis included gender, age, study enrollment sites, education, employment, and marital status. Body mass index (BMI) was categorized as low (<18·5kg/m^2^), normal (18·5 kg/m^2^–24·9 kg/m^2^) or high (≥25 kg/m^2^). Current CD4 count was categorized as <500 vs ≥500 cells/mm^3^. Other exploratory variables considered included duration on ART (0·5–2 years, 2–5 years, ≥5 years), history of fever in the past week (Yes vs No), adherence (zero days of ART missed vs at least one day of ART missed in the past month), ART regimen type (1^st^ line as efavirenz/nevirapine based regimens vs 2^nd^ line as lopinavir-ritonavir/atazanavir-ritonavir based regimens), drug or alcohol use (no drugs or <3 drinks per day vs. drug use or ≥3 drinks per day).

### Statistical analysis

Participants were dichotomized as those with and without virologic failure at their most recent visit. Characteristics were compared across the two groups using chi-squared testing for categorical variables and Kruskall-Wallis test for continuous variables. Generalized linear models with a robust standard error and Poisson distribution were used to estimate unadjusted, site-adjusted, and fully adjusted relative risks (RRs) and 95% confidence intervals (CIs) for associations between factors of interest and virologic failure. Backwards selection with 0.05 significance level was used to select variables for inclusion in the final multivariable models. We repeated the analyses examining our two secondary outcomes, viraemia and persistent viraemia. All analyses were performed using Stata 14·0 (StataCorp, College Station, Texas).

### Ethics

The study was approved by institutional review boards of the Walter Reed Army Institute of Research, Makerere University School of Public Health, Kenya Medical Research Institute, Tanzania National Institute of Medical Research, and Nigerian Ministry of Defence. Each participant provided informed consent that was documented with a signature or fingerprint if illiterate.

### Role of the funding source

This work was primarily funded by PEPFAR. The funding source had no role in the study design, data collection, data analysis, data interpretation, or writing of the report. The authors had full access to all the data related to this analysis and independently made the decision to submit the findings for publication.

## Results

**Fig 2:**
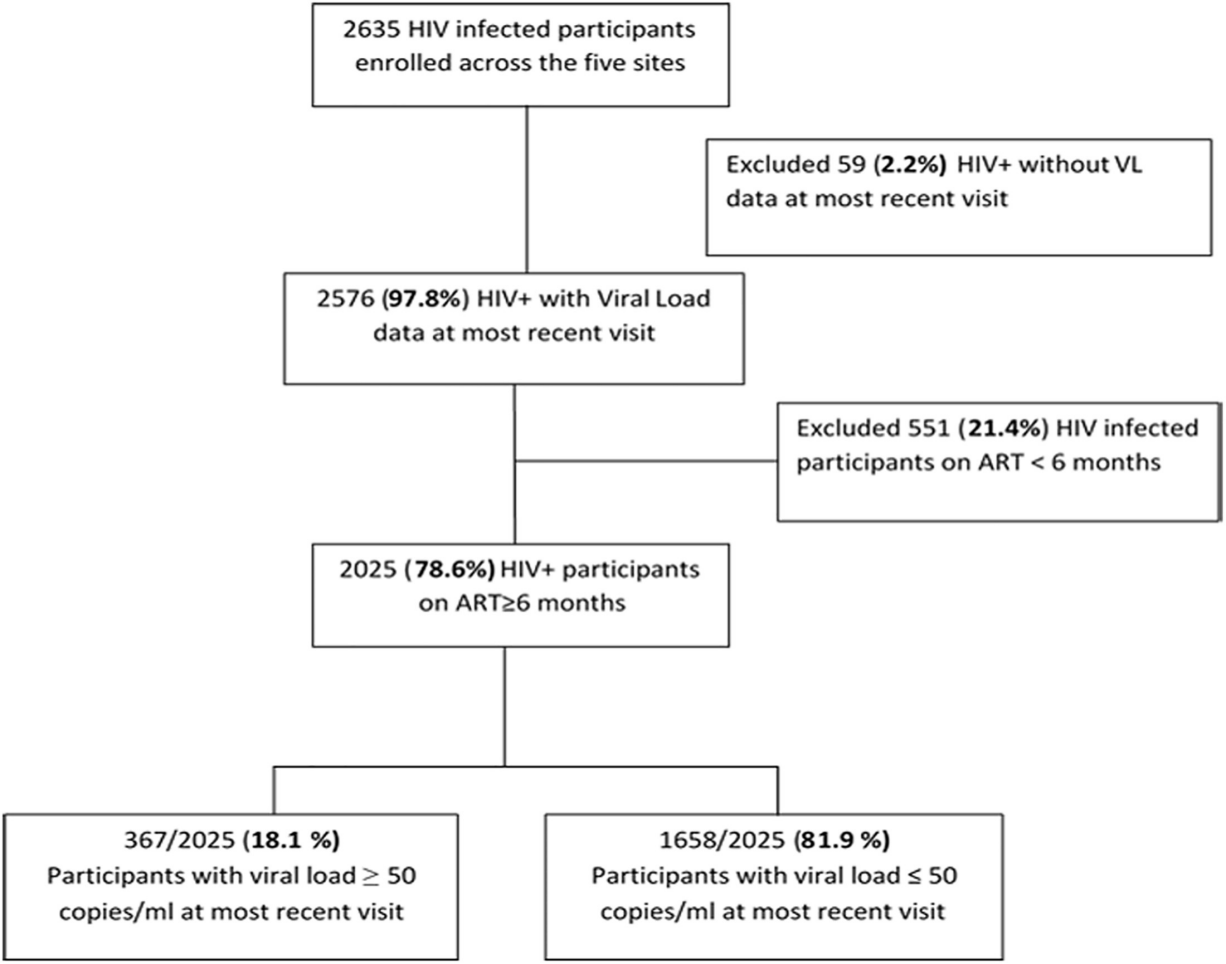
Flow diagram of AFRICOS participants included in the current analysis.

Between January 2013 and October 2017, 2678 HIV-infected participants enrolled in AFRICOS. Of these, 2577 (96·2%) had viral load data for the most recent visit. After excluding 523 participants on ART for less than six months, 2054 HIV-infected participants were included in the final analysis (Fig 2).

### Demographic and clinical characteristics

Participants were enrolled from sites at the South Rift Valley-Kenya (n = 820, 39·9%), Kisumu-Kenya (n = 365, 17·8%), Uganda (n = 352, 17·1%), Tanzania (n = 307, 14·9%) and Nigeria (n = 210, 10·2%). Most participants were female (57·4%), aged 40 years or older (60·5%), married or cohabiting (59·4%), and had some form of employment (59·2%). Overall, 4·1% of all participants reported a history of illicit drug use or consumption of ≥ 3 drinks of alcohol per day. The majority of participants (89·2%) were on 1^st^ line therapy; and 73% had been on ART for at least 2 years (Table 1).

**Table 1:** Socio-demographic and clinical characteristics of 2054 HIV infected AFRICOS-participants by viral load category at the most recent visit.

Level	Total on ART ≥ 6 months (N = 2054)	Viral Load category (copies/mL)		p-value^∞^
		Viral Load <1000 (n = 1870)	Viral Load > = 1000 (n = 184)	
**AFRICOS Site**				
Uganda	352 (17.1%)	340 (18.2%)	12 (6.5%)	<0.001
SRV,Kenya	820 (39.9%)	751 (40.2%)	69 (37.5%)	
Kisumu, Kenya	365 (17.8%)	341 (18.2%)	24 (13.0%)	
Tanzania	307 (14.9%)	256 (13.7%)	51 (27.7%)	
Nigeria	210 (10.2%)	182 (9.7%)	28 (15.2%)	
**Age in Years**				
18–24	72 (3.5%)	58 (3.1%)	14 (7.6%)	<0.001
25–39	739 (36.0%)	657 (35.1%)	82 (44.6%)	
40–49	791 (38.5%)	729 (39.0%)	62 (33.7%)	
> = 50	452 (22.0%)	426 (22.8%)	26 (14.1%)	
**Participant’s Gender**				
Male	874 (42.6%)	793 (42.4%)	81 (44.0%)	0.67
Female	1180 (57.4%)	1077 (57.6%)	103 (56.0%)	
**Marital Status**				
Not Married	831 (40.5%)	745 (39.8%)	86 (46.7%)	0.072
Married	1220 (59.4%)	1122 (60.0%)	98 (53.3%)	
Unknown	3 (0.1%)	3 (0.2%)	0 (0.0%)	
**Highest Education Level**				
None or some primary	694 (33.8%)	648 (34.7%)	46 (25.0%)	0.027
Some secondary	789 (38.4%)	711 (38.0%)	78 (42.4%)	
Post-Secondary	568 (27.7%)	508 (27.2%)	60 (32.6%)	
Missing	3 (0.1%)	3 (0.2%)	0 (0.0%)	
**Current Employment Status**				
Yes	1216 (59.2%)	1089 (58.2%)	127 (69.0%)	0.005
No	835 (40.7%)	778 (41.6%)	57 (31.0%)	
Missing	3 (0.1%)	3 (0.2%)	0 (0.0%)	
**BMI Category** (kg/m^2^_)_				
<18.5	193 (9.4%)	174 (9.3%)	19 (10.3%)	0.89
18.5-<25	1277 (62.2%)	1163 (62.2%)	114 (62.0%)	
> = 25	584 (28.4%)	533 (28.5%)	51 (27.7%)	
**Duration on ART (Years)**				
0.5-<2	560 (27.3%)	503 (26.9%)	57 (31.0%)	0.33
2-<5	578 (28.1%)	524 (28.0%)	54 (29.3%)	
> = 5	916 (44.6%)	843 (45.1%)	73 (39.7%)	
**Current CD4 Count** (cells/**mm**^**3**^**)**				
<200	204 (9.9%)	135 (7.2%)	69 (37.5%)	<0.001
200–349	395 (19.2%)	354 (18.9%)	41 (22.3%)	
350–499	501 (24.4%)	465 (24.9%)	36 (19.6%)	
500+	954 (46.4%)	916 (49.0%)	38 (20.7%)	
**ART Regiment**				
1^st^ Line	1832 (89.2%)	1688 (90.3%)	144 (78.3%)	<0.001
2^nd^ Line	215 (10.5%)	178 (9.5%)	37 (20.1%)	
Other	7 (0.3%)	4 (0.2%)	3 (1.6%)	
Adherence (#days missed)				
None	1775 (86.4%)	1635 (87.4%)	140 (76.1%)	<0.001
Any	275 (13.4%)	232 (12.4%)	43 (23.4%)	
Missing	4 (0.2%)	3 (0.2%)	1 (0.5%)	
**Fever within the past week**				
No	2033 (99.0%)	1856 (99.3%)	177 (96.2%)	<0.001
Yes	21 (1.0%)	14 (0.7%)	7 (3.8%)	

^∞^p-value in this table indicates if the variables in question are significantly different without necessarily indicating the direction of the difference. P-value <0.005 indicates statistical significance.

**Fig 3:**
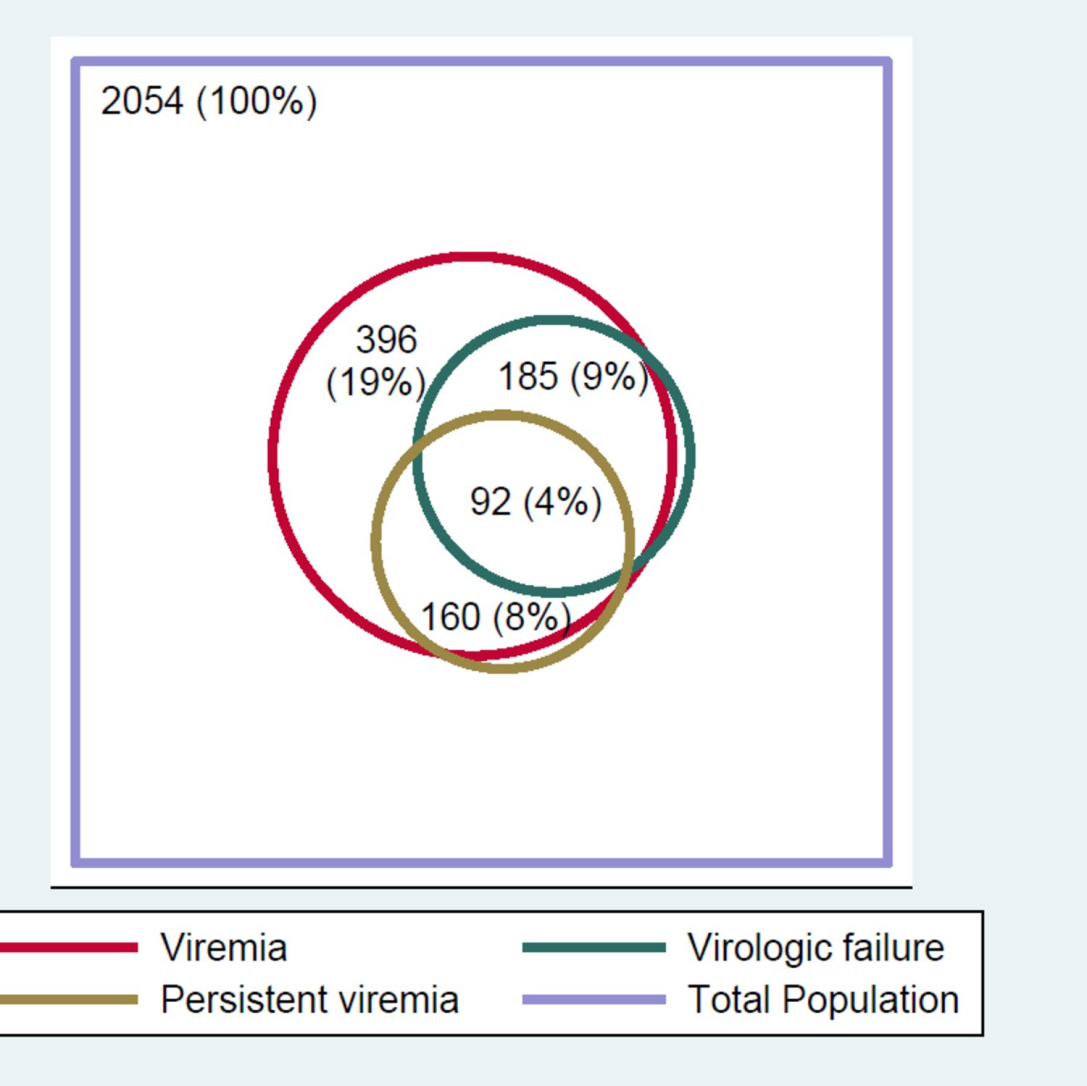
Overlap of participants with different thresholds of viraemia.

Of the 2054 eligible participants, 184 (9%) had virologic failure at the most recent visit, varying by study site with a low of 3.4% (12/352) at the Uganda site to a high of 16.6% (51/307) at the Tanzania site (Table 1). Viraemia (HIV RNA >50 copies/mL at the most recent visit) was observed in 19.3% (n-396) participants and persistent viraemia (HIV RNA >50 copies/mL at the two consecutive most recent visits) observed in 7·8% (n = 160) of participants. Of the participants with persistent viraemia, only 57.5% (92/160) had confirmed virologic failure, i.e. HIV RNA >1000 copies/mL at two consecutive visits (Fig 3).

### Predictors of virologic failure, viraemia and persistent viraemia

Results of the unadjusted and site adjusted analyses are shown in Tables 2 and 3. In the fully adjusted model, factors associated with increased risk of virologic failure at the most recent visit (Table 2) included being a participant at a site other than the Ugandan site, (SRV-Kenya aRR 2.6, 95%CI 1.40–4.91; Kisumu-Kenya aRR 2.2, 95%CI1.12–4.51; Tanzania aRR 4·3, 95%CI 2·29–8.21; and Nigeria aRR 3.3, 95%CI 1.68–6.52); being on 2^nd^ line ART (aRR 1.28, 95% CI 1. 8–2.66); a history of fever in the past week (aRR 3.7, 95% CI 1·69–8.05), and missing any day of ART dosing/adherence (aRR 1·8, 95% CI 1.27–2.57). The factors that were associated with decreased risk for virologic failure included having a current CD4 count ≥ 500 cells/mm^3^ (aRR 0·30, 95%CI, 0.23–0.49); older age (aRR 0·4, 95%CI 0·22–0·0.84); and high BMI (aRR 0·7, 95% CI 0·56–0·94).

**Table 2:** Unadjusted and adjusted factors associated with virologic failure (Viral load≥1000 copies/ml).

Characteristics	Unadjusted		Site adjusted		Multivariate	
	RR	95% CI	RR	95% CI	RR	95% CI
**HIV Program**						
Uganda	1		-	-	1	
SRV, Kenya	2.5	1.35–4.50	-	-	2.6	1.40–4.91
Kisumu, Kenya	1.9	0.98–3.80	-	-	2.2	1.12–4.51
Tanzania	4.9	2.65–8.97	-	-	4.3	2.29–8.21
Nigeria	3.9	2.03–7.52	-	-	3.3	1.68–6.52
**Age at most recent visit (years)**						
≤24	1		1		1	
25–39	0.6	0.34–0.95	0.7	0.40–1.14	0.9	0.48–1.61
40–49	0.4	0.24–0.68	0.5	0.28–0.81	0.6	0.32–1.11
≥ 50	0.3	0.16–0.54	0.3	0.19–0.65	0.4	0.22–0.84
**Gender**						
Male	1		1			
Female	0.9	0.71–1.24	0.9	0.70–1.21	-	-
**Marital Status**					-	-
Not Married	1		1			
Married	0.8	0.59–1.02	0.8	0.63–1.09	-	-
**Education level**						
None	1		1		-	-
Primary	1.5	1.05–2.12	1.1	0.76–1.59	-	-
Secondary School or above	1.6	1.10–2.30	1.0	0.69–1.57	-	-
**Employment status**						
Employed	1		1		-	
Unemployed	0.6	0.48–0.88	0.7	0.45–1.09	-	-
**Body Mass Index (kg/m2)**						
<18.5	1		1		1	
18.5 to 24.99	0.9	0.57–1.44	0.76	0.48–1.21	0.7	0.56–0.94
>25	0.9	0.54–1.46	0.64	0.38–1.06	0.7	0.50–0.90
**CD4 at most recent visit (cells/mm**^**3**^**)**						
500 plus	1		1		1	
Less 200	8.5	5.9–12.1	7.6	5.3–10.9	6.9	4.7–10.2
200–349	2.8	1.9–4.3	2.7	1.8–4.0	2.5	1.6–3.8
350–499	1.8	1.1–2.7	1.7	1.1–2.6	1.7	1.1 2.6
Less than 500	1		1		1	
At least 500	0.3	0.21–0.42	0.3	0.22–0.45	0.3	0.23–0.49
**Duration on ART (Years)**						
Less than 2 years	1		1		-	-
2–<5	0.9	0.64–1.31	0.9	0.63–1.28	-	-
≥5 years	0.8	0.56–1.09	0.7	0.52–1.00	-	-
**ART regimen type**						
First line, NNRTI-based	1		1		1	
Second line, PI-based	2.2	1.57–3.05	2.0	1.47–2.87	1.8	1.28–2.66
Other	5.4	2.28–13.01	4.8	1.99–11.37	3.8	1.18–11.98
**History of fever**						
No	1		1		1	
Yes	3.8	2.06–7.12	4.1	2.33–7.15	3.7	1.69–8.05
**Adherence (# days missed)**						
None	1		1		1	
Any days	2.0	1.44–2.72	2.0	1.40–2.73	1.8	1.27–2.57
**Current Alcohol, & Drug Use**						
No	1		1		-	-
Yes	1.2	0.64–2.27	1.1	0.58–2.08	-	-

**Table 3:** Unadjusted and adjusted factors associated with viraemia.

Characteristics	Viraemia (Viral Load > 50copies/mL)				Persistent Viraemia (Viral Load > 50copies/mL at 2 consecutive visits)			
	Unadjusted		Multivariate		Unadjusted		Multivariate^¶^	
	RR	95% CI	RR	95% CI	RR	95% CI	RR	95% CI
**HIV Program**								
Uganda	1		1		1		1	
SRV, Kenya	2.0	1.33–2.99	1.6	1.04–2.59	3.1	1.50–6.46	2.3	1.15–4.68
Kisumu, Kenya	2.1	1.33–3.23	1.7	1.01–2.75	2.7	1.25–6.01	2.9	1.34–6.15
Tanzania	5.9	4.00–8.73	4.5	2.81–7.13	7.6	3.66–15.75	4.9	2.36–10.13
Nigeria	3.7	2.43–5.74	3.7	2.43–5.72	5.8	2.69–12.40	4.5	2.20–9.35
**Age at most recent visit (Years)**								
≤24	1		-	-	1		1	
25–39	0.6	0.44–0.90	-	-	0.3	0.21–0.54	0.7	0.44–1.25
40–49	0.5	0.38–0.78	-	-	0.2	0.15–0.41	0.5	0.32–0.96
≥ 50	0.5	0.32–0.70	-	-	0.2	0.14–0.41	0.5	0.28–0.85
**Gender**								
Male	1		-		1		-	
Female	0.87	0.72–1.03	-	-	1.0	0.73–1.32	-	-
**Current Marital Status**								
Not Married	1				1		1	
Married	0.77	0.65–0.92	-	-	0.6	0.46–0.82	0.7	0.54–0.97
**Education level**								
None	1		-	-	1		-	-
Primary	1.54	1.23–1.93	-	-	1.4	0.96–2.04	-	-
> = Secondary School	1.53	1.20–1.95	-	-	1.8	1.20–2.57	-	-
**Employment status**								
Employed	1		1		1		-	
Unemployed	0.62	0.51–0.75	0.7	0.54–0.93	0.6	0.43–0.81	0.6	0.38–1.00
**Body Mass Index (kg/m2)**								
<18.5	1		1		1		-	
18.5 to 24.99	0.80	0.60–1.04	0.7	0.56–0.94	0.8	0.50–1.24	-	-
≥25	0.77	0.57–1.04	0.7	0.50–0.90	0.7	0.43–1.18	-	-
**CD4 at most recent visit (cells/mm**^**3**^**)**								
Less than 500	1		1		1			
At least 500	0.6	0.47–0.68	0.63	0.52–0.765	0.3	0.24–0.48	0.4	0.28–0.58
**Duration on ART (Years)**								
Less than 2 years	1		-	-	1		-	-
2–<5	1.0	0.79–1.27	-	-	0.8	0.54–1.27	-	-
≥5 years	0.97	0.78–1.20	-	-	1.0	0.68–1.45	-	-
**ART regimen type**								
1^st^ Line	1		1		1		1	
2^nd^ Line	2.3	1.91–2.82	2.1	1.70–2.50	3.9	2.93–5.28	3.0	2.26–4.10
Other	2.5	1.08–6.03	1.5	0.82–2.59	4.9	1.57–15.54	3.8	1.40–0.47
**History of fever**								
No	1		1		1		-	
Yes	2.2	1.37–3.73	1.7	1.14–2.68	2.5	1.03–5.97	-	-
**Adherence (# days missed)**								
None	1		1		1		-	
Any days	1.4	1.12–1.76	1.3	1.03–1.60	1.4	1.00–2.10	-	-
**Current Alcohol, & Drug Use**								
No	1		-	-	1		-	-
Yes	1.4	0.96–2.01	-	-	1.7	0.96–3.00	-	-

^¶^Backwards selection with 0.05 significance level was used to select variables for inclusion in the final multivariable models

Viraemia and persistent viraemia had similar risk factors as those identified for virologic failure, including being on 2^nd^ line ART, being a participant at a site other than the Ugandan site, and current CD4 count < 500 cells/mm^3^ (Table 3). Similar to the findings for virologic failure, adherence and history of fever in the past week increased the risk of viraemia but were not significantly associated with persistent viraemia.

In contrast to the findings for virologic failure, unemployment was protective for viraemia at the most recent visit and persistent viraemia (Table 3). Older participants were less likely to have persistent viraemia (aRR 0·5, 95% CI 0·28–0·85), while being married (aRR 0·7, 95% CI 0·53–0·93) was only protective for persistent viraemia (Table 2).

## Discussion

Our study evaluated the prevalence of virologic failure and factors associated with virologic failure in a large African HIV cohort receiving ART based on the WHO’s public health approach. Our findings showed that viraemia at the most recent visit occurred commonly, while persistent viraemia and virologic failure occurred less frequently.

Our findings further demonstrate that the site one attended for their ART care, the current CD4 count, and the ART regimen type were consistently associated with adverse virologic outcomes irrespective of the specific definition used. Other factors like low BMI, fever in the past week, and adherence were only associated with single measurement viraemia and virologic failure but not persistent viraemia. Younger age was only associated with persistent viraemia and virologic failure but not viraemia.

Our findings are significant in three important ways: first, viraemia as used in this analysis represents the definition of viral non-suppression (plasma HIV RNA > 50 copies/mL) as used in the high-income countries [24, 25] while virologic failure represents the definition of viral non-suppression (plasma HIV RNA > 1000 copies/mL) used by the WHO Public health approach for low and middle income countries [26]. As such the rate of viral suppression observed will depend on the threshold of viral suppression used. Second, this analysis indicates that only 57.5% of participants with persistent viraemia (plasma HIV RNA > 50 copies/mL at two consecutive visits) will also have confirmed virologic failure, highlighting a need for reviewing the WHO’s threshold for viral suppression for the LMICs. Last, the findings demonstrate that ART programs in the regions are moving at different paces towards the 3^rd^ UNAIDS 90 target and are not homogenous. This may be attributed to the different levels of quality of care that the programs have or the nature of the HIV epidemic in the different regions. Similar variation in the prevalence of virologic failure by geographic location has been reported before in other settings [27, 28]. Program level differences, for example unique site level processes, cultural differences or other factors such as variations in policies and their implementation [29], might explain some of these observations. It is also possible that these differences may be a reflection of the variation in the predominant HIV subtypes circulating in the participating sites. In Uganda and Kenya the predominant HIV subtypes are D and A, while in Tanzania it is subtype C and A, and in Nigeria subtype G and CRF02_AG predominate [30]. Whereas some published literature found an association between HIV subtype and treatment outcomes [31, 32], other studies have found no such association [33, 34]. In general, studies have found better ART response for HIV non-B compared to B subtypes [31], although a 2014 study in Spain found a lower virologic response on ART for HIV subtypes F compared to B subtypes [35].

We also found that younger age was significantly associated with virologic failure but not viraemia. Whereas some published studies have found that socio-demographic factors like age are associated with virologic suppression [36–39], other studies have found no such association [40, 41]. In general, younger age is usually associated with inadequate adherence to ART [42, 43], due to a number of unique behavioural and psychosocial factors like anxiety, stigma, lack of disclosure and low social economic status. This finding highlights the value of focusing on the special needs of young HIV-infected patients if we are to achieve the UNAIDS third 90 target.

Last, being on a second-line ART regimen was significantly associated with risk of virologic failure, independent of adherence. This is in line with findings reported by previous studies that have shown being on second line therapy is associated with viraemia and virologic failure [44]. Patients who are usually on second line therapy are those who have already failed on an earlier regimen. Although poor adherence on first line therapy usually predicts poor adherence on second therapy [45], the observed association between 2^nd^ line therapy and virologic failure in this study appears to be independent of adherence or other measured factors like the current CD4 count, BMI or clinical site.

The authors recognize four major limitations of the analysis. First, the cross sectional nature of the analysis does not allow for causal inference or calculations of rates of failure. Second, these analyses do not include underlying HIV drug resistance that could be a driver of virologic failure. There is variability in the rates of baseline/pre-treatment HIVDR resistance in the literature. For example, while some reports indicate low rates of transmitted HIVDR [46], the WHO HIV drug resistance report 2017 reported very high rates of up to 15.4%, in East Africa, 7.2% in West Africa, and 11% in Southern Africa. Future analysis of data from our cohort will evaluate the contribution of HIV drug resistance to virologic failure. Third, adherence data was based on self-report, which may be impacted by recall and social desirability bias. Last, the finding that a sizable proportion of participants with persistent viraemia did not have virologic failure confirmed as per WHO guidelines, although not a major objective of the current analysis, is potentially significant as it suggests a need for a review of the current WHO viral suppression threshold for LMICs in order to achieve the 3^rd^ of the UNAIDS 90-90-90 targets. We are aware of randomised trials like the SESOTHO clinical trial [47] that are examining the subject of the WHO threshold for viral suppression for the LMICs that will include data on clinical outcomes for patients with persistent viraemia, which will complement the current analysis. Despite these limitations we would like to highlight two major strengths of our study: 1) all participants were monitored and received standardized laboratory and clinical evaluations allowing for accurate measurements, 2) a constant and adequate sample size for the majority of variables considered for this analysis, strengthening the robustness of the analysis results. Therefore, it is likely that our results are a reflection of the ART programs in these countries.

## Conclusions

In conclusion, this multi-center analysis demonstrates that HIV-infected patients established on ART for more than six months in the African setting frequently experienced viraemia while continuing to be on ART. We have shown that clinical care sites home to AFRICOS are different and that further analyses will be required to elucidate on the unique features of these sites. We have also demonstrated that low BMI, low CD4 count, and history of fever in the past week remain important predictors of virologic failure that should be used as a marker for intensified adherence counselling especially in the absence of reliable or readily available viral load monitoring. Finally, younger age and single marital status are associated with virologic failure, which has implications for programmatic treatment monitoring practices.

## Supporting information

S1 PDF African Cohort Study blank CRFs. (PDF)

S2 PDF African Cohort Study blank subject questionnaire. (PDF)
