# Supplementary material for: HIV virologic failure and its predictors among HIV-infected adults on antiretroviral therapy in the African Cohort Study
Source: PLoS One. 2019 Feb 5;14(2):e0211344. doi: 10.1371/journal.pone.0211344 (PMC6363169; doi:10.1371/journal.pone.0211344)
Supplement: S1 PDF — (PDF) [file pone.0211344.s001.pdf]

Annotated

Case Report Forms

For

Protocol RV329 / WRAIR 1897

Version 1.7

Dec. 26, 2013

African Cohort Study (AFRICOS)

Study Conducted by US Military HIV Research Program

Study Supported by

Data Coordinating and Analysis Center, (DCAC), MHRP

Henry M. Jackson Foundation (HJF)

Summary of RV329 CRFs

Case Report Form	Page #	Total #	Visit	Source Document
1. Eligibility / Enrollment	4	1	Initial [1]	x
2. Demographics	5	1	Initial	x
3. HIV Status	6	1	All [2]	x
4. Medical Record Extraction (HIV Positive)	7	1	Initial	x
5. WHO Classification	8	3	Initial	x
6. Medical History	11	4	Initial	x
7. Medication Record	15	3	All	x
8. Recent Symptoms	18	2	All	x
9. Vital Signs / Physical Exam	20	1	All	x
10. Specimen Collection	21	1	All	x
11. Blood Chemistry	22	1	All	
12. Hematology	23	1	All	
13. Urinalysis	24	1	All	
14. Lymphocyte Subset Profile	25	1	All	
15. Additional Microbiology	26	1	All	
16. Viral Load (HIV Positive)	27	1	All	
17. Serology (HIV Positive & Negative)	28	1	All	
18. Serology (HIV Negative Only)	29	1	All	
19. Resistance Test (HIV Positive)	30	1	All [3]	
20. Cognitive Evaluation	31	2	Annual	x
21. Current Medical Conditions	33	1	SV	x
22. Acute Febrile Illness	34	1	All [3]	x
23. Hospitalization / Serious/Acute Visit	35	1	All [3]	x
24. Women's Health (Female)	36	1	All	x
25. Past/Current Obstetric History (Female)	37	2	All	x
26. Cervical Cancer Screening (Female)	39	1	Annual	x
27. Missed Visit	40	1	SV [3]	
28. Status Change	41	1	SV [3]	
29. Pathology	42	1	All [3]	

Initial = Visit 1 (initial visit)
SV = Subsequent Visits
All = both Initial and all Subsequent Visits
Annual=Initial Visit and all Annual SVs

[1] Eligibility / Enrollment CRF also used in cases of re-enrollment
[2] Questions 2d – 2f apply to all scheduled visits; see CRF for details
[3] As needed

RV329 AFRICOS – CASE REPORT FORMS

MENU OF CODE LISTS

CODE	CRF	Version
**ClinPlus-Formatted Code Lists**		
PROGRAM CODE	All	1.0
SITE CODE	All	1.0
STUDY CODE	CRF 1	1.0
DIAGNOSIS METHOD CODE	CRF 5, 6, 21, 23	1.0
STOP CODE	CRF 7	1.0
LABORATORY CODE	CRF 11-19	1.0
SPECIMEN CODE	CRF 15, 29	1.0
TREATMENT CODE	CRF 23	1.0
TREATMENT OUTCOME CODE	CRF 21, 23	1.0
**Non-Formatted Code Lists**		
DIAGNOSIS CODE	CRF 6, 7, 21 – 23, 28, 29	1.0
MEDICATION CODE	CRF 7, 10, 19	1.0
ORGANISM CODE	CRF 15	1.0

ELIGIBILITY / ENROLLMENT

Subject ID: |__|__|__|__|-|__|__|__|__| SUBJID Visit Date: |__|__|-|__|__|__|-|__|__|__|__| VISITDT Visit: |____| VISIT

HIV Negative (Must be completed prior to Protocol Enrollment)

Inclusion Criteria	Yes 1	No 0
	YES	NO.
1. Consents to HIV testing, and to pre-test and post-test counseling N_INC1	○	○
2. Adult aged 18 years or older N_INC2	○	○
3. Ability and willingness to sign/mark/thumbprint the Informed Consent form N_INC3	○	○
4. Intends to be a long term resident of the area (i.e., for at least 24 months) N_INC4	○	○
5. Willing to provide location or contact information and be contacted by study staff N_INC5	○	○
6. Consents to data and specimen collection and storage for future use N_INC6	○	○
7. Must understand English or local language as approved by IRB N_INC7	○	○
**Exclusion Criteria**		
1. The subject is known to be HIV infected N_EXC1	○	○
2. Any significant condition (including medical and psychological/psychiatric disorder) that in the opinion of the study investigator might interfere with the conduct of the study N_EXC2	○	○
3. The subject is known to be pregnant N_EXC3	○	○
4. The subject is currently a prisoner N_EXC4	○	○

HIV Positive (Must be completed prior to Protocol Enrollment)

Inclusion Criteria	Yes 1	No 0
	YES	NO.
1. Known HIV infection P_INC1	○	○
2. Adult aged 18 years or older P_INC2	○	○
3. Ability and willingness to sign/mark/thumbprint the Informed Consent form P_INC3	○	○
4. Intends to be a long term resident of the area and to undergo long term HIV care at AFRICOS participating clinic (i.e., for at least 24 months) P_INC4	○	○
5. Willing to provide location or contact information and to be contacted by study staff P_INC5	○	○
6. Consents to data and specimen collection and storage for future use P_INC6	○	○
7. Must understand English or local language as approved by IRB P_INC7	○	○
**Exclusion Criteria**		
1. Any significant condition (including medical and psychological/psychiatric disorder) that in the opinion of the study investigator might interfere with the conduct of the study P_EXC1	○	○
2. The subject is known to be pregnant P_EXC2	○	○
3. The subject is currently a prisoner P_EXC3	○	○

Protocol Enrollment

1. Did subject meet the eligibility criteria? MEETCRIT ○ 1. Yes ○ 0. No YESNO.
2. Did subject sign an Informed Consent Form? SIGNFORM ○ 1. Yes ○ 0. No YESNO.
3. Date Informed Consent was signed: CONSENDT Date: |__|__|-|__|__|__|-|__|__|__|__|
4. Did subject agree to host genetic testing? GENTEST ○ 1. Yes ○ 0. No YESNO.
5. Subject was recruited for enrollment based on ***(Mark all that apply)***:
   - ☐ a. Representative of general clinic population CLINPOP
   - ☐ b. Identified as member of a serodiscordant couple SEROCOUP
   - ☐ c. Subject in prior study (refer to ***Study Code List***): PRISTUDY |__|__|__| Study #: |PRINUM ____| PRICODE STDYCODE.
   - ☐ d. Suspected long term non-progressor NONPROG
   - ☐ z. Other, *Specify*: OTHER OTH_TXT ____________

Form Completed by: FORMBY SIGNED. ____________ Date: |__|__|-|__|__|__|-|__|__|__|__| FORMDT

QC/QA: QC_QA SIGNED. ________ Data Entry: 1st ________ 2nd ________

DEMOGRAPHICS

Subject ID: |__|__|__|-|__|__|__|__|__| SUBJID **Visit Date:** |__|__|-|__|__|__|-|__|__|__|__| VISITDT **Visit:** |______| VISIT

1. Date of Birth: DOBDT |__|__|-|__|__|__|-|__|__|__|__|

2. Gender: GENDER GENDER.
   - ◯ 1. Male
   - ◯ 2. Female

3. Tribal:

   3a. **Kenya** TRIBAL_K TRIBAL_K.
   - ◯ 1. Kalenjin ◯ 2. Kisii ◯ 3. Luhya ◯ 4. Luo
   - ◯ 90. Other, *Specify*: TRIBTXTK ____________

   3b. **Nigeria** TRIBAL_N TRIBAL_N.
   - ◯ 1. Hausa ◯ 2. Yoruba ◯ 3. Igbo ◯ 90. Other, *Specify*: TRIBTXTN ____________

   3c. **Tanzania** TRIBAL_T TRIBAL_T.
   - ◯ 1. Nyakyus ◯ 2. Safwa ◯ 3. Nyiha ◯ 4. Ndali ◯ 5. Kinga
   - ◯ 90. Other, *Specify*: TRIBTXTT ____________

   3d. **Uganda** TRIBAL_U TRIBAL_U.
   - ◯ 1. Ganda ◯ 2. Soga ◯ 3. Gisu ◯ 90. Other, *Specify*: TRIBTXTU ____________

4. Religion: RELIGION RELIGION.
   - ◯ 1. Catholic Christian
   - ◯ 2. Non-Catholic Christian
   - ◯ 3. Muslim
   - ◯ 4. Traditionalist
   - ◯ 7. Don't Know
   - ◯ 8. Refused
   - ◯ 90. Other, *Specify*: RELIGTXT ____________

5. At what age did you have your first sexual intercourse? FIRSTSEX FIRSTSEX.
   - ◯ 0. Never had sex
   - ◯ 1. < 13
   - ◯ 2. Between 13 – 18
   - ◯ 3. > 18
   - ◯ 8. Refused

Form Completed by: **FORMBY** SIGNED. ____________ Date: FORMDT |__|__|-|__|__|__|-|__|__|__|__|

QC/QA: ____________ **Data Entry: 1st** ____________ **2nd** ____________

HIV STATUS

Subject ID: |__|__|__|-|__|__|__|__| SUBJID **Visit Date:** |__|__|-|__|__|__|-|__|__|__|__| VISITDT **Visit:** |____| VISIT

1. Date of last Negative HIV test (**Visit 1 only**): |__|__|-|__|__|__|-|__|__|__|__| NTESTDT

2. HIV Status (**Visit 1 only**): HIVSTAT NEGPOSNK.

○ 0. Negative → ***(if 0. Negative, Skip to 3.)***
○ 1. Positive* → ***(if 1. Positive, Continue to 2a.)***
○ 7. Unknown → ***(if 7. Unknown, Skip to 3.)***

***If Positive:**

Visit 1 only:

2a. Diagnosis Date: |__|__|-|__|__|__|-|__|__|__|__| DIAGDT

2b. Date first enrolled in HIV care: |__|__|-|__|__|__|-|__|__|__|__| ENROLDT

2c. Date enrolled in HIV care at this clinic: |__|__|-|__|__|__|-|__|__|__|__| ENROCDT

Visit 1 or at Subsequent Visit if subject not medically eligible at Visit 1:

→ *If subject is not medically eligible for ART at Visit 1, mark "NA, not on ART."*
→ *If subject becomes medically eligible for ART after Visit 1, complete 2d., 2e., and 2f. for the appropriate visit number and leave all other questions on the form blank.*

2d. Date medically eligible for ART: |__|__|-|__|__|__|-|__|__|__|__| ELIGDT ○ **NA, not on ART** NANOTART

2e. Why was the subject eligible for ART? ***(Mark all that apply)***

☐ a. Clinical stage ELIG_A – ELIG_D
☐ b. Low CD4 count
☐ c. High Viral Load ☐ d. Plan B+
○ 7. Unknown ELIG_NK

2f. Date started ART: |__|__|-|__|__|__|-|__|__|__|__| ART_SDT

3. Is the subject's partner currently enrolled in RV329? PARTENRL YESNONA.

○ 1. Yes* ○ 0. No ○ 5. NA

3a. ***If Yes,**
Partner's RV329 Subject ID: |__|__|__|-|__|__|__|__| PTSUBJID

Form Completed by: FORMBY SIGNED. ______________________ Date: |__|__|-|__|__|__|-|__|__|__|__| FORMDT

QC/QA: ____________ Data Entry: 1st ____________ 2nd ____________

MEDICAL RECORD EXTRACTION

Page/Seq. #: |____| SEQUENCE

Subject ID: |__|__|__|-|__|__|__|__|__| SUBJID Visit Date: |__|__|-|__|__|__|-|__|__|__|__| VISITDT Visit: |____| VISIT

○ Mark if All Unknown (no medical data) ALLUNK ○ NA (HIV Negative) NAHIVNEG

Data closest to the time of HIV Diagnosis (1-9):

1. WHO Clinical Stage: WHOSTG WHOSTG.
○ 1. I ○ 2. II ○ 3. III ○ 4. IV ○ 7. Unknown Date: |__|__|-|__|__|__|-|__|__|__|__| WHOSTGDT

2. Function: FUNCTION FUNCTION. ○ 1. Unimpaired ○ 2. Impaired but ambulatory ○ 3. Bedridden ○ 7. Unknown Date: |__|__|-|__|__|__|-|__|__|__|__| FUNCDT

3. Height: |______| cm HT Date: |__|__|-|__|__|__|-|__|__|__|__| HTDT

4. Weight: |____.__| kg WT Date: |__|__|-|__|__|__|-|__|__|__|__| WTDT

5. CD4: |________| cells/uL CD4N Draw Date: |__|__|-|__|__|__|-|__|__|__|__| CD4DT
|____.__| % CD4P Draw Date: |__|__|-|__|__|__|-|__|__|__|__| CD4PDT

6. Hemoglobin: |____.__| g/dL HEMOG Draw Date: |__|__|-|__|__|__|-|__|__|__|__| HEMOGDT

7. ALT/SGPT: |______| U/L ALT Draw Date: |__|__|-|__|__|__|-|__|__|__|__| ALTDT

8. Creatinine: CREAT |____.__| ○ 1. µmol/L ○ 2. mg/dL CREAT_UN MGDL_U. Draw Date: |__|__|-|__|__|__|-|__|__|__|__| CREATDT

9. HIV-1 RNA: RNA_CIRC *Circle One:* < = > LESEQGR. |______________| copies/mL RNA
○ Not Detected NODETECT Draw Date: |__|__|-|__|__|__|-|__|__|__|__| RNADT

10. Record the lowest CD4 cell count (NADIR) subject attained since HIV Diagnosis:
CD4: |______| cells/uL CD4NADIR Draw Date: |__|__|-|__|__|__|-|__|__|__|__| NADIRDT

11. Record the CD4 cell count at the time of ART initiation:
CD4: |______| cells/uL CD4ARTIN Draw Date: |__|__|-|__|__|__|-|__|__|__|__| ARTINDT

12. Virologic monitoring: ○ None Available VL_NONE FIELD_DA.
[1] *Record CD4 Count if within 30 Days of Viral Load*
[2] *Record on Resistance Test CRF*

	Viral Load Draw Date	CD3+CD4+ count (CD4 count)[1]	% CD3+CD4+ [1]	Viral Load Copies/mL	Resistance Test[2]
	VLDT_A-J	VLCD4_A-J	VLCD4P_A-J	VL_LGE_A-J VLCOPY_A-J	RESDN_A-J
a.	|__|__|-|__|__|__|-|__|__|__|__|	|________| /mm3	|____.__| %	|________|	☐ Done
b.	|__|__|-|__|__|__|-|__|__|__|__|	|________| /mm3	|____.__| %	LESEQGR. |________|	☐ Done
c.	|__|__|-|__|__|__|-|__|__|__|__|	|________| /mm3	|____.__| %	|________|	☐ Done
d.	|__|__|-|__|__|__|-|__|__|__|__|	|________| /mm3	|____.__| %	|________|	☐ Done

Form Completed by: FORMBY SIGNED. ________________ Date: |__|__|-|__|__|__|-|__|__|__|__| FORMDT

QC/QA: __________ Data Entry: 1st __________ 2nd __________

MEDICAL RECORD EXTRACTION

Page/Seq. #: |____|

Subject ID: |__|__|__|-|__|__|__|__|__| **Visit Date:** |__|__|-|__|__|__|-|__|__|__|__| **Visit:** |____|

nahivneg

12. Virologic monitoring: Allunk ○ None Available Vl_none

[1] *Record CD4 Count if within 30 Days of Viral Load*

[2] *Record on Resistance Test CRF*

	Viral Load Draw Date	CD3+CD4+ count (CD4 count)[1]	% CD3+CD4+ [1]	Viral Load Copies/mL	Resistance Test [2]
	Vl_d vl_m vl_y		vlcd4p Vl_lge	vlcopy	resdn
a.	|__|__|-|__|__|__|-|__|__|__|__|	|__________| /mm3 vlcd4	|_____.____| %	|__________|	☐ Done
b.	|__|__|-|__|__|__|-|__|__|__|__|	|__________| /mm3	|_____.____| %	|__________|	☐ Done
c.	|__|__|-|__|__|__|-|__|__|__|__|	|__________| /mm3	|_____.____| %	|__________|	☐ Done
d.	|__|__|-|__|__|__|-|__|__|__|__|	|__________| /mm3	|_____.____| %	|__________|	☐ Done

Form Completed by: FORMBY SIGNED. ____________________ Date: |__|__|-|__|__|__|-|__|__|__|__| FORMDT

QC/QA: __________ Data Entry: 1st __________ 2nd __________

SEQUENCED

WHO CLASSIFICATION (page 1 of 3)

Subject ID: |__|__|__|-|__|__|__|__| SUBJID Visit Date: |__|__|-|__|__|__|-|__|__|__|__| VISITDT Visit: |____| VISIT

○ Asymptomatic HIV Infection only*, or ○ HIV Negative with none of conditions below* → *End Form.
ASYMPTOM NEGNONE

Otherwise capture all past and current conditions listed for WHO Classification pages 1-3.

WHO CLASSIFICATION (Mark all that apply)	# of episodes	Start Date (First Episode)	Stop Date (Last Episode)	Ongoing Mark if ongoing	DX Method[1]
**1. WHO Stage I** WHO1A_3G	EPISODES	STARTDT	STOPDT	ONGO	DX_METH
☐ a. Persistent Generalized Lymphadenopathy (W10.0) WHO1A_3G.	|____|	|__|__|__|-|__|__|__|__|	|__|__|__|-|__|__|__|__|	○	|____| DXMETHOD.
**2. WHO Stage II**					
☐ a. Herpes Zoster (within last 5 years) (W20.0)	|____|	|__|__|__|-|__|__|__|__|	|__|__|__|-|__|__|__|__|	○	|____|
☐ b. Minor Mucocutaneous Manifestations (W21.0)	|____|	|__|__|__|-|__|__|__|__|	|__|__|__|-|__|__|__|__|	○	|____|
☐ c. Recurrent Upper Respiratory Tract Infections (W22.0)	|____|	|__|__|__|-|__|__|__|__|	|__|__|__|-|__|__|__|__|	○	|____|
☐ d. Weight Loss ≤ 10% of Body Weight (W23.0)	|____|	|__|__|__|-|__|__|__|__|	|__|__|__|-|__|__|__|__|	○	|____|
**3. WHO Stage III**					
☐ a. Severe Bacterial Infections (i.e. Pneumonia) (W30.0)	|____|	|__|__|__|-|__|__|__|__|	|__|__|__|-|__|__|__|__|	○	|____|
☐ b. Candidiasis–Oral (Thrush) (W31.0)	|____|	|__|__|__|-|__|__|__|__|	|__|__|__|-|__|__|__|__|	○	|____|
☐ c. Unexplained Chronic Diarrhea (>1 month) (W32.0)	|____|	|__|__|__|-|__|__|__|__|	|__|__|__|-|__|__|__|__|	○	|____|
☐ d. Oral Hairy Leukoplakia (W33.0)	|____|	|__|__|__|-|__|__|__|__|	|__|__|__|-|__|__|__|__|	○	|____|
☐ e. Unexplained Prolonged Fever (intermittent or constant, >1 month) (W34.0)	|____|	|__|__|__|-|__|__|__|__|	|__|__|__|-|__|__|__|__|	○	|____|
☐ f. Weight loss >10% of Body Weight (W35.0)	|____|	|__|__|__|-|__|__|__|__|	|__|__|__|-|__|__|__|__|	○	|____|
☐ g. Pulmonary Tuberculosis					
○ g1. Smear + (W36.1)	|____|	|__|__|__|-|__|__|__|__|	|__|__|__|-|__|__|__|__|	○	|____|
○ g2. Smear – (W36.2)	|____|	|__|__|__|-|__|__|__|__|	|__|__|__|-|__|__|__|__|	○	|____|
○ g6. Smear not done (W36.3)	|____|	|__|__|__|-|__|__|__|__|	|__|__|__|-|__|__|__|__|	○	|____|

[1]*Refer to Diagnosis Method Code List*

SEQUENCED

WHO CLASSIFICATION (page 2 of 3)

Subject ID: |__|__|__|-|__|__|__|__| SUBJID Visit Date: |__|__|-|__|__|__|-|__|__|__|__| VISITDT Visit: |__| VISIT

WHO CLASSIFICATION (Mark all that apply)	# of episodes	Start Date (First Episode)	Stop Date (Last Episode)	Ongoing Mark if ongoing	DX Method[1]
4. WHO Stage IV WHO4A_H	EPISODES	STARTDT	STOPDT	ONGO	DX_METH
a. Candidiasis WHO4A_H.					DXMETHOD.
☐ a1. Esophageal (W40.1)	|__|	|__|__|__|-|__|__|__|__|	|__|__|__|-|__|__|__|__|		
☐ a2. Lungs (W40.2)	|__|	|__|__|__|-|__|__|__|__|	|__|__|__|-|__|__|__|__|	○	|__|
b. Extrapulmonary cryptococcal					
☐ b1. CNS/Meningitis (W41.1)	|__|	|__|__|__|-|__|__|__|__|	|__|__|__|-|__|__|__|__|	○	|__|
☐ b2. Skin (W41.2)	|__|	|__|__|__|-|__|__|__|__|	|__|__|__|-|__|__|__|__|	○	|__|
☐ c. Cryptosporidiosis with Diarrhea (>1 month) (W42.0)	|__|	|__|__|__|-|__|__|__|__|	|__|__|__|-|__|__|__|__|	○	|__|
d. CMV Disease (other than spleen, liver, lymph nodes)					
☐ d1. Esophagitis (W43.1)	|__|	|__|__|__|-|__|__|__|__|	|__|__|__|-|__|__|__|__|	○	|__|
☐ d2. Retinitis (W43.2)	|__|	|__|__|__|-|__|__|__|__|	|__|__|__|-|__|__|__|__|	○	|__|
☐ d3. Pneumonia (W43.3)	|__|	|__|__|__|-|__|__|__|__|	|__|__|__|-|__|__|__|__|	○	|__|
☐ d4. Colitis (W43.4)	|__|	|__|__|__|-|__|__|__|__|	|__|__|__|-|__|__|__|__|	○	|__|
☐ d5. CNS (W43.5)	|__|	|__|__|__|-|__|__|__|__|	|__|__|__|-|__|__|__|__|	○	|__|
e. Herpes simplex					
☐ e1. Mucocutaneous >1month (W44.1)	|__|	|__|__|__|-|__|__|__|__|	|__|__|__|-|__|__|__|__|	○	|__|
☐ e2. Any visceral (W44.2)	|__|	|__|__|__|-|__|__|__|__|	|__|__|__|-|__|__|__|__|	○	|__|
☐ f. HIV Encephalopathy (AIDS dementia complex) (W45.0)	|__|	|__|__|__|-|__|__|__|__|	|__|__|__|-|__|__|__|__|	○	|__|
☐ g. HIV Wasting Syndrome (W46.0)	|__|	|__|__|__|-|__|__|__|__|	|__|__|__|-|__|__|__|__|	○	|__|
h. Kaposi's Sarcoma (KS)					
☐ h1. Skin (W47.1)	|__|	|__|__|__|-|__|__|__|__|	|__|__|__|-|__|__|__|__|	○	|__|
☐ h2. Gastrointestinal (W47.2)	|__|	|__|__|__|-|__|__|__|__|	|__|__|__|-|__|__|__|__|	○	|__|
☐ h3. Pulmonary (W47.3)	|__|	|__|__|__|-|__|__|__|__|	|__|__|__|-|__|__|__|__|	○	|__|
☐ h90. Other (W47.9): *Specify:* ______ H90_TXT	|__|	|__|__|__|-|__|__|__|__|	|__|__|__|-|__|__|__|__|	○	|__|

[1]*Refer to Diagnosis Method Code List*

QC/QA: ____________ Data Entry: 1st ____________ 2nd ____________

SEQUENCED

WHO CLASSIFICATION (page 3 of 3)

Subject ID: |__|__|__|__|-|__|__|__|__|__| SUBJID Visit Date: |__|__|__|-|__|__|__|-|__|__|__|__| VISITDT Visit: |__| VISIT

WHO CLASSIFICATION (Mark all that apply)	# of episodes	Start Date (First Episode)	Stop Date (Last Episode)	Ongoing Mark if ongoing	DX Method[1]
4. WHO Stage IV (Cont'd) WHO4I_P WHO4I_P.	EPISODES	STARTDT	STOPDT	ONGO	DX_METH DXMETHOD.
**i. Lymphoma**					
☐ i1. Primary CNS (W48.1)	|__|	|__|__|__|-|__|__|__|__|__|	|__|__|__|-|__|__|__|__|__|	○	|__|
☐ i2. Burkitts (W48.2)	|__|	|__|__|__|-|__|__|__|__|__|	|__|__|__|-|__|__|__|__|__|	○	|__|
☐ i3. Diffuse Large B Cell (W48.3)	|__|	|__|__|__|-|__|__|__|__|__|	|__|__|__|-|__|__|__|__|__|	○	|__|
☐ i4. Other Non-Hodgkins (W48.4): *Specify:* I4_TXT	|__|	|__|__|__|-|__|__|__|__|__|	|__|__|__|-|__|__|__|__|__|	○	|__|
☐ i5. Hodgkins (W48.5)	|__|	|__|__|__|-|__|__|__|__|__|	|__|__|__|-|__|__|__|__|__|	○	|__|
**j. Atypical (non-TB) Mycobacteria**					
☐ j1. Pulmonary (W49.1)	|__|	|__|__|__|-|__|__|__|__|__|	|__|__|__|-|__|__|__|__|__|	○	|__|
☐ j2. Systemic (W49.2)	|__|	|__|__|__|-|__|__|__|__|__|	|__|__|__|-|__|__|__|__|__|	○	|__|
**k. Tuberculosis, extra pulmonary**					
☐ k1. Meningitis (W50.1)	|__|	|__|__|__|-|__|__|__|__|__|	|__|__|__|-|__|__|__|__|__|	○	|__|
☐ k2. Miliary (W50.2)	|__|	|__|__|__|-|__|__|__|__|__|	|__|__|__|-|__|__|__|__|__|	○	|__|
☐ k3. Abdominal (W50.3)	|__|	|__|__|__|-|__|__|__|__|__|	|__|__|__|-|__|__|__|__|__|	○	|__|
☐ k4. Scrofula (Lymph Nodes) (W50.4)	|__|	|__|__|__|-|__|__|__|__|__|	|__|__|__|-|__|__|__|__|__|	○	|__|
☐ k90. Other (W50.9): *Specify:* K90_TXT	|__|	|__|__|__|-|__|__|__|__|__|	|__|__|__|-|__|__|__|__|__|	○	|__|
☐ **l. Progressive Multifocal Leukoencephalopathy (W51.0)**	|__|	|__|__|__|-|__|__|__|__|__|	|__|__|__|-|__|__|__|__|__|	○	|__|
☐ **m. Mycosis, Disseminated endemic (W52.0)** *Specify:* M_TXT	|__|	|__|__|__|-|__|__|__|__|__|	|__|__|__|-|__|__|__|__|__|	○	|__|
☐ **n. Pneumocystic Carinii Pneumonia (PCP) (W53.0)**	|__|	|__|__|__|-|__|__|__|__|__|	|__|__|__|-|__|__|__|__|__|	○	|__|
☐ **o. Salmonella Septicemia, Non-typhoid (W54.0)**	|__|	|__|__|__|-|__|__|__|__|__|	|__|__|__|-|__|__|__|__|__|	○	|__|
☐ **p. Toxoplasmosis, CNS (W55.0)**	|__|	|__|__|__|-|__|__|__|__|__|	|__|__|__|-|__|__|__|__|__|	○	|__|

[1] *Refer to Diagnosis Method Code List*

Form Completed by: FORMBY SIGNED. ______________________ Date: |__|__|__|-|__|__|__|-|__|__|__|__| FORMDT

QC/QA: ____________ Data Entry: 1st ____________ 2nd ____________

SEQUENCED

MEDICAL HISTORY (page 1 of 4)

Subject ID: |__|__|__|-|__|__|__|__|__| SUBJID Visit Date: |__|__|-|__|__|__|-|__|__|__|__| VISITDT Visit: |____| VISIT

○ Mark if None (all 4 pages) NONE

Record General Medical History and Current Conditions.
If Illness not listed, record Diagnosis on Medical History (page 4 of 4).

**Diagnosis** *(Mark all that apply)*	# of episodes	Start Date (First Episode)	Stop Date (Last Episode)	Ongoing Mark if ongoing	DX Method[1]
**1. Heart Disease** DX1A_7E	EPISODES	STARTDT	STOPDT	ONGO	DX_METH
☐ a. Heart attack (myocardial infarction) (I21)	|___|	|__|__|__|-|__|__|__|__|	|__|__|__|-|__|__|__|__|		DXMETHOD.
☐ b. Heart Failure (I50) DX1A_7E.	|___|	|__|__|__|-|__|__|__|__|	|__|__|__|-|__|__|__|__|	○	|____|
☐ c. Angina (I20)	|___|	|__|__|__|-|__|__|__|__|	|__|__|__|-|__|__|__|__|	○	|____|
☐ **2. High blood pressure/ Essential HTN (I10)**	|___|	|__|__|__|-|__|__|__|__|	|__|__|__|-|__|__|__|__|	○	|____|
**3. Kidney Disease**					
☐ a. Chronic kidney disease (N18)	|___|	|__|__|__|-|__|__|__|__|	|__|__|__|-|__|__|__|__|	○	|____|
☐ b. Kidney stones (N20)	|___|	|__|__|__|-|__|__|__|__|	|__|__|__|-|__|__|__|__|	○	|____|
**4. Diabetes**					
☐ a. Type I (E10)	|___|	|__|__|__|-|__|__|__|__|	|__|__|__|-|__|__|__|__|	○	|____|
☐ b. Type II (E11)	|___|	|__|__|__|-|__|__|__|__|	|__|__|__|-|__|__|__|__|	○	|____|
**5. Anemia**					
☐ a. Related to medication (D61.1)	|___|	|__|__|__|-|__|__|__|__|	|__|__|__|-|__|__|__|__|	○	|____|
☐ b. Related to pregnancy (O99.0)	|___|	|__|__|__|-|__|__|__|__|	|__|__|__|-|__|__|__|__|	○	|____|
**6. Mental health**					
☐ a. Depression (F33)	|___|	|__|__|__|-|__|__|__|__|	|__|__|__|-|__|__|__|__|	○	|____|
☐ b. Alcoholism (F10.2)	|___|	|__|__|__|-|__|__|__|__|	|__|__|__|-|__|__|__|__|	○	|____|
**7. Liver Problem**					
☐ a. Acute hepatitis A (B15)	|___|	|__|__|__|-|__|__|__|__|	|__|__|__|-|__|__|__|__|	○	|____|
☐ b. Chronic hepatitis B (B18.1)	|___|	|__|__|__|-|__|__|__|__|	|__|__|__|-|__|__|__|__|	○	|____|
☐ c. Chronic Hepatitis C (B18.2)	|___|	|__|__|__|-|__|__|__|__|	|__|__|__|-|__|__|__|__|	○	|____|
☐ d. Hepatitis, Alcoholic (K70.1)	|___|	|__|__|__|-|__|__|__|__|	|__|__|__|-|__|__|__|__|	○	|____|
☐ e. Cirrhosis (K74)	|___|	|__|__|__|-|__|__|__|__|	|__|__|__|-|__|__|__|__|	○	|____|

[1] *Refer to Diagnosis Method Code List*

SEQUENCED

MEDICAL HISTORY (page 2 of 4)

Subject ID: |__|__|__|-|__|__|__|__| SUBJID Visit Date: |__|__|-|__|__|__|-|__|__|__|__| VISITDT Visit: |____| VISIT

If Illness not listed, record Diagnosis on Medical History (page 4 of 4)

**Diagnosis** *(Mark all that apply)*	# of episodes	Start Date (First Episode)	Stop Date (Last Episode)	Ongoing Mark if ongoing	DX Method[1]
**8. Cancer** DX8A_10F	EPISODES	STARTDT	STOPDT	ONGO	DX_METH
☐ a. Multiple Myeloma (C90.0)	|____|	|__|__|__|-|__|__|__|__|	|__|__|__|-|__|__|__|__|		DXMETHOD.
☐ b. Lung (C34) DX8A_10F.	|____|	|__|__|__|-|__|__|__|__|	|__|__|__|-|__|__|__|__|	O	|____|
☐ c. Breast (C50)	|____|	|__|__|__|-|__|__|__|__|	|__|__|__|-|__|__|__|__|	O	|____|
☐ d. Cervix (invasive, not in situ) (C53)	|____|	|__|__|__|-|__|__|__|__|	|__|__|__|-|__|__|__|__|	O	|____|
☐ e. Prostate (C61)	|____|	|__|__|__|-|__|__|__|__|	|__|__|__|-|__|__|__|__|	O	|____|
☐ f. Skin Cancer, melanoma (C43)	|____|	|__|__|__|-|__|__|__|__|	|__|__|__|-|__|__|__|__|	O	|____|
**9. Sexually transmitted infections**					
☐ a. Syphilis, primary (A51.0)	|____|	|__|__|__|-|__|__|__|__|	|__|__|__|-|__|__|__|__|	O	|____|
☐ b. Gonorrhea (A54)	|____|	|__|__|__|-|__|__|__|__|	|__|__|__|-|__|__|__|__|	O	|____|
☐ c. Nongonococcal urethritis (N34.1)	|____|	|__|__|__|-|__|__|__|__|	|__|__|__|-|__|__|__|__|	O	|____|
☐ d. Genital Herpes (A60)	|____|	|__|__|__|-|__|__|__|__|	|__|__|__|-|__|__|__|__|	O	|____|
☐ e. Chlamydia (A56.0)	|____|	|__|__|__|-|__|__|__|__|	|__|__|__|-|__|__|__|__|	O	|____|
☐ f. Genital or anal warts (A63.0)	|____|	|__|__|__|-|__|__|__|__|	|__|__|__|-|__|__|__|__|	O	|____|
☐ g. PID (Pelvic Inflammatory Disease) (N73.9)	|____|	|__|__|__|-|__|__|__|__|	|__|__|__|-|__|__|__|__|	O	|____|
☐ h. Trichomoniasis (A59)	|____|	|__|__|__|-|__|__|__|__|	|__|__|__|-|__|__|__|__|	O	|____|
**10. Neurologic**					
☐ a. Migraine (G43)	|____|	|__|__|__|-|__|__|__|__|	|__|__|__|-|__|__|__|__|	O	|____|
☐ b. Epilepsy/Seizures (G40)	|____|	|__|__|__|-|__|__|__|__|	|__|__|__|-|__|__|__|__|	O	|____|
☐ c. Drug–induced neuropathy (G62.0)	|____|	|__|__|__|-|__|__|__|__|	|__|__|__|-|__|__|__|__|	O	|____|
☐ d. Dementia (Non-AIDS) (F03)	|____|	|__|__|__|-|__|__|__|__|	|__|__|__|-|__|__|__|__|	O	|____|
☐ e. Traumatic brain injury (with loss of consciousness) (S06.2)	|____|	|__|__|__|-|__|__|__|__|	|__|__|__|-|__|__|__|__|	O	|____|
☐ f. Stroke (I64)	|____|	|__|__|__|-|__|__|__|__|	|__|__|__|-|__|__|__|__|	O	|____|

[1] *Refer to Diagnosis Method Code List*

QC/QA: ____________ Data Entry: 1st ____________ 2nd ____________

SEQUENCED

MEDICAL HISTORY (page 3 of 4)

Subject ID: |__|__|__|-|__|__|__|__|__| SUBJID Visit Date: |__|__|__|-|__|__|__|-|__|__|__|__| VISITDT Visit: |______| VISIT

If Illness not listed, record Diagnosis on Medical History (page 4 of 4)

**Diagnosis** *(Mark all that apply)*	# of episodes	**Start Date** (First Episode)	**Stop Date** (Last Episode)	**Ongoing** Mark if ongoing	DX Method[1]
**11. Diarrhea over 4 weeks** DX11A17X DX11A17X.	EPISODES	STARTDT	STOPDT	ONGO	DX_METH
☐ a. From medicine (R19.8)	|__|	|__|__|__|-|__|__|__|__|__|	|__|__|__|-|__|__|__|__|__|		DXMETHOD.
☐ b. From other reason not related to medicine (R19.4)	|__|	|__|__|__|-|__|__|__|__|__|	|__|__|__|-|__|__|__|__|__|	○	|__|
☐ **12. Acute Pancreatitis (K85)**	|__|	|__|__|__|-|__|__|__|__|__|	|__|__|__|-|__|__|__|__|__|	○	|__|
**13. Lipodystrophy**					
☐ a. Buffalo hump (dorsocervical fat pad) (E65.0)	|__|	|__|__|__|-|__|__|__|__|__|	|__|__|__|-|__|__|__|__|__|	○	|__|
☐ b. Loss of subcutaneous at in arms/legs (E88.1)	|__|	|__|__|__|-|__|__|__|__|__|	|__|__|__|-|__|__|__|__|__|	○	|__|
☐ c. Accumulation of fat around the waist (E65.1)	|__|	|__|__|__|-|__|__|__|__|__|	|__|__|__|-|__|__|__|__|__|	○	|__|
☐ d. Facial wasting appearance (M62.5)	|__|	|__|__|__|-|__|__|__|__|__|	|__|__|__|-|__|__|__|__|__|	○	|__|
☐ **14. Osteoarthritis (M15.9)**	|__|	|__|__|__|-|__|__|__|__|__|	|__|__|__|-|__|__|__|__|__|	○	|__|
**15. Thyroid disease**					
☐ a. Low thyroid (E03.9)	|__|	|__|__|__|-|__|__|__|__|__|	|__|__|__|-|__|__|__|__|__|	○	|__|
☐ b. High thyroid (E05)	|__|	|__|__|__|-|__|__|__|__|__|	|__|__|__|-|__|__|__|__|__|	○	|__|
**16. Bone density loss**					
☐ a. Osteoporosis without fracture (M81)	|__|	|__|__|__|-|__|__|__|__|__|	|__|__|__|-|__|__|__|__|__|	○	|__|
☐ b. Hip fracture (S72.0)	|__|	|__|__|__|-|__|__|__|__|__|	|__|__|__|-|__|__|__|__|__|	○	|__|
☐ c. Osteoporosis with fracture (M80)	|__|	|__|__|__|-|__|__|__|__|__|	|__|__|__|-|__|__|__|__|__|	○	|__|
☐ **17. Malaria, severe (B50.8)**	|__|	|__|__|__|-|__|__|__|__|__|	|__|__|__|-|__|__|__|__|__|	○	|__|

[1] *Refer to Diagnosis Method Code List*

QC/QA: ____________ Data Entry: 1st ____________ 2nd ____________

SEQUENCED

MEDICAL HISTORY (page 4 of 4)

Page: |____|

Subject ID: |__|__|__|-|__|__|__|__| SUBJID Visit Date: |__|__|-|__|__|__|-|__|__|__|__| VISITDT Visit: |____| VISIT

Record other unlisted Medical History and Current Conditions

**Diagnosis** *(Refer to Diagnosis Code List)*	# of episodes	Start Date (First Episode)	Stop Date (Last Episode)	Ongoing Mark if ongoing	DX Method[1]
**18. Other unlisted illness**	EPISODES	STARTDT	STOPDT	ONGO	DX_METH DXMETHOD.
a. Name: DX_NAME ______ Code: DX_CODE* |__|__|__|.|__|__|	|____|	|__|__|__|-|__|__|__|__|	|__|__|__|-|__|__|__|__|	O	|____|
b. Name: ______ Code: |__|__|__|.|__|__|	|____|	|__|__|__|-|__|__|__|__|	|__|__|__|-|__|__|__|__|	O	|____|
c. Name: ______ Code: |__|__|__|.|__|__|	|____|	|__|__|__|-|__|__|__|__|	|__|__|__|-|__|__|__|__|	O	|____|
d. Name: ______ Code: |__|__|__|.|__|__|	|____|	|__|__|__|-|__|__|__|__|	|__|__|__|-|__|__|__|__|	O	|____|
e. Name: ______ Code: |__|__|__|.|__|__|	|____|	|__|__|__|-|__|__|__|__|	|__|__|__|-|__|__|__|__|	O	|____|
f. Name: ______ Code: |__|__|__|.|__|__|	|____|	|__|__|__|-|__|__|__|__|	|__|__|__|-|__|__|__|__|	O	|____|
g. Name: ______ Code: |__|__|__|.|__|__|	|____|	|__|__|__|-|__|__|__|__|	|__|__|__|-|__|__|__|__|	O	|____|
h. Name: ______ Code: |__|__|__|.|__|__|	|____|	|__|__|__|-|__|__|__|__|	|__|__|__|-|__|__|__|__|	O	|____|

[1]*Refer to Diagnosis Method Code List*

Form Completed by: FORMBY SIGNED. ______________ Date: |__|__|-|__|__|__|-|__|__|__|__| FORMDT

QC/QA: ________ Data Entry: 1st ________ 2nd ________

SEQUENCED

MEDICATION RECORD (page 1 of 3)

Subject ID: |__|__|__|-|__|__|__|__|__| SUBJID **Visit Date:** |__|__|-|__|__|__|-|__|__|__|__| VISITDT **Visit:** |______| VISIT

Mark if None (Never taken ARVs) NONE

Enter fixed dose combinations on ***Medication Record Page 3, Other ARV Medication Use****; refer to the Medication code list for the appropriate code .*

Visit 1: *Record medications subject is currently taking, and also prior antiretroviral regimens.* ***Subsequent Visits****: Refer to medications listed on the this form from the last visit, record medications the subject is still taking and medications the subject has stopped and why (stop code), and add any medications since the last visit.*

****PREP****=Pre Exposure Prophylaxis,* ****PMTCT****=Prevention of Mother To Child Transmission,* ****PEP****=Post Exposure Prophylaxis*

Medication (Mark all that apply) MEDICAT MEDICAT.	Indication* IND_A IND_B IND_C IND_D	Start Date STARTDT	Stop Date STOPDT ONGO	Stop Code[1] STOPCODE STOPCODE.
☐ 1. Abacavir (ABC) 300mg	☐ a. PREP ☐ c. HIV Treatment ☐ b. PMTCT ☐ d. PEP	|__|__|__|-|__|__|__|__|	|__|__|__|-|__|__|__|__| ○ Ongoing	|__|__|
☐ 2. Didanosine (ddI) 400mg	☐ a. PREP ☐ c. HIV Treatment ☐ b. PMTCT ☐ d. PEP	|__|__|__|-|__|__|__|__|	|__|__|__|-|__|__|__|__| ○ Ongoing	|__|__|
☐ 3. Lamivudine (3TC) 150mg	☐ a. PREP ☐ c. HIV Treatment ☐ b. PMTCT ☐ d. PEP	|__|__|__|-|__|__|__|__|	|__|__|__|-|__|__|__|__| ○ Ongoing	|__|__|
☐ 4. Lamivudine (3TC) 300mg	☐ a. PREP ☐ c. HIV Treatment ☐ b. PMTCT ☐ d. PEP	|__|__|__|-|__|__|__|__|	|__|__|__|-|__|__|__|__| ○ Ongoing	|__|__|
☐ 5. Stavudine (d4T) 30mg	☐ a. PREP ☐ c. HIV Treatment ☐ b. PMTCT ☐ d. PEP	|__|__|__|-|__|__|__|__|	|__|__|__|-|__|__|__|__| ○ Ongoing	|__|__|
☐ 6. Tenofovir (TDF) 300mg	☐ a. PREP ☐ c. HIV Treatment ☐ b. PMTCT ☐ d. PEP	|__|__|__|-|__|__|__|__|	|__|__|__|-|__|__|__|__| ○ Ongoing	|__|__|
☐ 7. Zidovudine (AZT) 300mg	☐ a. PREP ☐ c. HIV Treatment ☐ b. PMTCT ☐ d. PEP	|__|__|__|-|__|__|__|__|	|__|__|__|-|__|__|__|__| ○ Ongoing	|__|__|
☐ 8. Efavirenz (EFV) 600mg	☐ a. PREP ☐ c. HIV Treatment ☐ b. PMTCT ☐ d. PEP	|__|__|__|-|__|__|__|__|	|__|__|__|-|__|__|__|__| ○ Ongoing	|__|__|
☐ 9. Nevirapine (NVP) 200mg	☐ a. PREP ☐ c. HIV Treatment ☐ b. PMTCT ☐ d. PEP	|__|__|__|-|__|__|__|__|	|__|__|__|-|__|__|__|__| ○ Ongoing	|__|__|
☐ 10. Atazanavir (ATZ) 300mg	☐ a. PREP ☐ c. HIV Treatment ☐ b. PMTCT ☐ d. PEP	|__|__|__|-|__|__|__|__|	|__|__|__|-|__|__|__|__| ○ Ongoing	|__|__|
☐ 11. Atazanavir/ Ritonavir 300mg/100mg	☐ a. PREP ☐ c. HIV Treatment ☐ b. PMTCT ☐ d. PEP	|__|__|__|-|__|__|__|__|	|__|__|__|-|__|__|__|__| ○ Ongoing	|__|__|
☐ 12. Lopinavir/Ritonavir 400mg/100mg	☐ a. PREP ☐ c. HIV Treatment ☐ b. PMTCT ☐ d. PEP	|__|__|__|-|__|__|__|__|	|__|__|__|-|__|__|__|__| ○ Ongoing	|__|__|
☐ 13. Ritonavir Boost 100mg	☐ a. PREP ☐ c. HIV Treatment ☐ b. PMTCT ☐ d. PEP	|__|__|__|-|__|__|__|__|	|__|__|__|-|__|__|__|__| ○ Ongoing	|__|__|
☐ 14. Emtricitabine (FTC) 200mg	☐ a. PREP ☐ c. HIV Treatment ☐ b. PMTCT ☐ d. PEP	|__|__|__|-|__|__|__|__|	|__|__|__|-|__|__|__|__| ○ Ongoing	|__|__|

Does the pill count confirm adherence to ARVs? ○ 1. Yes ○ 0. No ○ 7. Not Performed PILL_CT YESNONP.

[1] *Refer to Stop Code List; Enter Primary Stop Code only*

QC/QA: ____________ Data Entry: 1st ____________ 2nd ____________

SEQUENCED

MEDICATION RECORD (page 2 of 3)

Subject ID: |__|__|__|-|__|__|__|__|__| SUBJID Visit Date: |__|__|-|__|__|__|-|__|__|__|__| VISITDT Visit: |____| VISIT

Mark if None NONE

Indication	Medication *(Mark all that apply)*	Start Date STARTDT	Stop Date STOPDT	Ongoing (ONGO) Mark if ongoing	Stop Code[1] STOPCODE
1. PCP Prophylaxis PCPMED PCPMED.	a. Co-Trimoxazole (Septrin)	|__|__|__|-|__|__|__|__|__|	|__|__|__|-|__|__|__|__|__|	O	|__|__| STOPCODE.
	b. Dapsone	|__|__|__|-|__|__|__|__|__|	|__|__|__|-|__|__|__|__|__|	O	|__|__|
	c. Pentamidine	|__|__|__|-|__|__|__|__|__|	|__|__|__|-|__|__|__|__|__|	O	|__|__|
	z. Other, *Specify:* OTH_TXT1	|__|__|__|-|__|__|__|__|__|	|__|__|__|-|__|__|__|__|__|	O	|__|__|
2. Treatment of latent TB (IPT) TBIPTMED TBIPTMED.	a. Isoniazid	|__|__|__|-|__|__|__|__|__|	|__|__|__|-|__|__|__|__|__|	O	|__|__|
	b. Rifampicin	|__|__|__|-|__|__|__|__|__|	|__|__|__|-|__|__|__|__|__|	O	|__|__|
	z. Other, *Specify:* OTH_TXT2	|__|__|__|-|__|__|__|__|__|	|__|__|__|-|__|__|__|__|__|	O	|__|__|
3. TB Treatment (active) TBACTMED TBACTMED.	a. Rifampicin	|__|__|__|-|__|__|__|__|__|	|__|__|__|-|__|__|__|__|__|	O	|__|__|
	b. Isoniazid	|__|__|__|-|__|__|__|__|__|	|__|__|__|-|__|__|__|__|__|	O	|__|__|
	c. Streptomycin	|__|__|__|-|__|__|__|__|__|	|__|__|__|-|__|__|__|__|__|	O	|__|__|
	d. Ethambutol	|__|__|__|-|__|__|__|__|__|	|__|__|__|-|__|__|__|__|__|	O	|__|__|
	e. Pyrazinamide	|__|__|__|-|__|__|__|__|__|	|__|__|__|-|__|__|__|__|__|	O	|__|__|
	y. Other (1), *Specify:* OTH1TXT3	|__|__|__|-|__|__|__|__|__|	|__|__|__|-|__|__|__|__|__|	O	|__|__|
	z. Other (2), *Specify:* OTH2TXT3	|__|__|__|-|__|__|__|__|__|	|__|__|__|-|__|__|__|__|__|	O	|__|__|
4. Cryptococcus Treatment CRYTXMED CRYTXMED.	a. Fluconazole	|__|__|__|-|__|__|__|__|__|	|__|__|__|-|__|__|__|__|__|	O	|__|__|
	b. Amphotericin B	|__|__|__|-|__|__|__|__|__|	|__|__|__|-|__|__|__|__|__|	O	|__|__|
	z. Other, *Specify:* OTH_TXT4	|__|__|__|-|__|__|__|__|__|	|__|__|__|-|__|__|__|__|__|	O	|__|__|
5. Cryptococcus Prophylaxis	a. Fluconazole CRYPTMED CRYPTMED.	|__|__|__|-|__|__|__|__|__|	|__|__|__|-|__|__|__|__|__|	O	|__|__|

Medication Allergies: ***(Mark all that apply)***

- ☐ a. Sulfa (ie. Co-Trimoxazole) MED_A
- ☐ b. Penicillin MED_B
- ☐ y. Other (1), *Specify:* OTH1TXTY MED_Y
- ☐ z. Other (2), *Specify:* OTH2TXTZ MED_Z

[1]*Refer to Stop Code List; Enter Primary Stop Code only*

Form Completed by: FORMBY SIGNED. ____________________ Date: |__|__|-|__|__|__|-|__|__|__|__| FORMDT

QC/QA: ____________ Data Entry: 1st ____________ 2nd ____________

SEQUENCED

MEDICATION RECORD (page 3 of 3)

Page # |____|

Subject ID: |__|__|__|-|__|__|__|__|__| SUBJID Visit Date: |__|__|-|__|__|__|-|__|__|__|__| VISITDT Visit: |____| VISIT

*PREP= Pre Exposure Prophylaxis, *PMTCT= Prevention of Mother To Child Transmission, *PEP= Post Exposure Prophylaxis

Other ARV Medication Use:

Medication (CODE and NAME: refer to Medication Code List)	Indication*	Start Date	Stop Date	Ongoing Mark if ongoing	Stop Code[1]
SEQ. #: |____| SEQUENCE CODE: |__|__|__| ARV_CODE* NAME: ARV_NAME	☐ a. PREP ARVIND_A ☐ b. PMTCT ARVIND_B ☐ c. HIV Treatment ARVIND_C ☐ d. PEP ARVIND_D	ASTARTDT |__|__|__|-|__|__|__|__|	ASTOPDT |__|__|__|-|__|__|__|__|	ARVONGO ○	ASTOPCD |__|__| STOPCODE.
SEQ. #: |____| CODE: |__|__|__| NAME: ______	☐ a. PREP ☐ b. PMTCT ☐ c. HIV Treatment ☐ d. PEP	|__|__|__|-|__|__|__|__|	|__|__|__|-|__|__|__|__|	○	|__|__|
SEQ. #: |____| CODE: |__|__|__| NAME: ______	☐ a. PREP ☐ b. PMTCT ☐ c. HIV Treatment ☐ d. PEP	|__|__|__|-|__|__|__|__|	|__|__|__|-|__|__|__|__|	○	|__|__|
SEQ. #: |____| CODE: |__|__|__| NAME: ______	☐ a. PREP ☐ b. PMTCT ☐ c. HIV Treatment ☐ d. PEP	|__|__|__|-|__|__|__|__|	|__|__|__|-|__|__|__|__|	○	|__|__|

Other Medication Use: *(Please re-start sequencing)*

Medication (CODE and NAME: refer to Medication Code List)	Indication (refer to Diagnosis Code List)	Start Date	Stop Date	Ongoing Mark if ongoing	Stop Code[1]
SEQ. #: |____| SEQUENCE CODE: |__|__|__| MD_CODE* NAME: MD_NAME	MD_IND* |__|__|__|.|__|__|	MSTARTDT |__|__|__|-|__|__|__|__|	MSTOPDT |__|__|__|-|__|__|__|__|	MDONGO ○	MSTOPCD |__|__| STOPCODE.
SEQ. #: |____| CODE: |__|__|__| NAME: ______	|__|__|__|.|__|__|	|__|__|__|-|__|__|__|__|	|__|__|__|-|__|__|__|__|	○	|__|__|
SEQ. #: |____| CODE: |__|__|__| NAME: ______	|__|__|__|.|__|__|	|__|__|__|-|__|__|__|__|	|__|__|__|-|__|__|__|__|	○	|__|__|
SEQ. #: |____| CODE: |__|__|__| NAME: ______	|__|__|__|.|__|__|	|__|__|__|-|__|__|__|__|	|__|__|__|-|__|__|__|__|	○	|__|__|

[1] *Refer to Stop Code List; Enter Primary Stop Code only*

Form Completed by: FORMBY SIGNED. ______________ Date: |__|__|-|__|__|__|-|__|__|__|__| FORMDT

QC/QA: ________ Data Entry: 1st ________ 2nd ________

RECENT SYMPTOMS (page 1 of 2)

Subject ID: |__|__|__|-|__|__|__|__|__| SUBJID Visit Date: |__|__|-|__|__|__|-|__|__|__|__| VISITDT Visit: |______| VISIT

○ Mark if All Normal (both pages) ALLNORM *Record symptoms the subject has had within the past week*

Body System	*If Abnormal, Mark all that apply		Comments
**1. General** GEN<br>○ 0. Normal<br>○ 1. Abnormal *<br>NORMAB.	☐ a. Fever GEN_A	○ <1 week<br>○ 1 wk-1 month<br>○ >=1 month<br>GEN_A_D LGWKMON.	GEN_ATXT
	☐ b. Sweats GEN_B	○ <1 month<br>○ >=1 month<br>GEN_B_D LGMON.	GEN_BTXT
	☐ c. Weight loss GEN_C	○ <10 kg<br>○ 10-20 kg<br>○ >20 kg<br>GEN_C_WT LGWEIGHT.	GEN_CTXT
	☐ d. Fatigue GEN_D	○ <1 month<br>○ >=1 month<br>GEN_D_D LGMON.	GEN_DTXT
**2. HEENT** HNT<br>○ 0. Normal<br>○ 1. Abnormal *<br>NORMAB.	☐ a. Blurry vision HNT_A		HNT_ATXT
	☐ b. Eye pain HNT_B		HNT_BTXT
	☐ c. Swollen lymph nodes HNT_C		HNT_CTXT
	☐ d. Sore throat HNT_D		HNT_DTXT
	☐ e. Painful swallowing HNT_E		HNT_ETXT
	☐ f. Mouth lesions HNT_F		HNT_FTXT
**3. Cardio Pulmonary** CP<br>○ 0. Normal<br>○ 1. Abnormal *<br>NORMAB.	☐ a. Chest pain CP_A	○ 1. Exertional<br>○ 2. Pleuritic<br>CP_A_TYP EXEPLEU.	CP_ATXT
	☐ b. Cough CP_B	Duration: |______| Days CP_B_DUR<br>☐ Productive CP_B_PRD	CP_BTXT
	☐ c. Shortness of breath CP_C		CP_CTXT
**4. Gastrointestinal** GAS<br>○ 0. Normal<br>○ 1. Abnormal *<br>NORMAB.	☐ a. Diarrhea GAS_A	Duration: |______| Days GAS_ADUR	GAS_ATXT
	☐ b. Nausea GAS_B		GAS_BTXT
	☐ c. Anorexia GAS_C		GAS_CTXT
	☐ d. Vomiting GAS_D		GAS_DTXT
	☐ e. Abdominal pain GAS_E		GAS_ETXT

QC/QA: __________ Data Entry: 1st __________ 2nd __________

RECENT SYMPTOMS (page 2 of 2)

Subject ID: |__|__|__|-|__|__|__|__| SUBJID Visit Date: |__|__|-|__|__|__|-|__|__|__|__| VISITDT Visit: |______| VISIT

Body System	*If Abnormal, mark all that apply	Comments
**5. Genitourinary** GTY<br>○ 0. Normal<br>○ 1. Abnormal *<br>NORMAB.	☐ a. Vaginal or penile discharge GTY_A	GTY_ATXT
	☐ b. Genital ulcer GTY_B	GTY_BTXT
	☐ c. Blood in urine GTY_C	GTY_CTXT
	☐ d. Burning/Painful urination GTY_D	GTY_DTXT
	☐ e. Vaginal itching GTY_E	GTY_ETXT
	☐ f. Painful intercourse GTY_F	GTY_FTXT
	☐ g. Lower abdominal pain GTY_G	GTY_GTXT
	☐ h. Swollen lymph nodes at groin GTY_H	GTY_HTXT
	☐ i. Genital warts GTY_I	GTY_ITXT
**6. Musculoskeletal** MUS<br>○ 0. Normal<br>○ 1. Abnormal *<br>NORMAB.	☐ a. Arthritis/Swollen joints MUS_A	MUS_ATXT
	☐ b. Joint aches MUS_B	MUS_BTXT
	☐ c. Muscle aches MUS_C	MUS_CTXT
**7. Central Nervous System** CNS<br>○ 0. Normal<br>○ 1. Abnormal *<br>NORMAB.	☐ a. Headache CNS_A	CNS_ATXT
	☐ b. Neck stiffness CNS_B	CNS_BTXT
	☐ c. Confusion CNS_C	CNS_CTXT
	☐ d. Numbness/loss of sensation CNS_D	CNS_DTXT
	☐ e. Loss of balance CNS_E	CNS_ETXT
**8. Skin** SKN NORMAB.<br>○ 0. Normal<br>○ 1. Abnormal *	☐ a. Jaundice SKN_A	SKN_ATXT
	☐ b. Rash SKN_B ☐ Pruritic SKN_BPRU<br>Duration: |______| days SKN_BDUR	SKN_BTXT
**9. Other Body System (1):**<br>OTBS1TXT<br>________	*Specify Symptom(s):*<br>OTSY1TXT<br>________	OTH1_TXT
**10. Other Body System (2):**<br>OTBS2TXT<br>________	*Specify Symptom(s):*<br>OTSY2TXT<br>________	OTH2_TXT

Form Completed by: FORMBY SIGNED. ____________ Date: |__|__|-|__|__|__|-|__|__|__|__| FORMDT

QC/QA: ____________ Data Entry: 1st ____________ 2nd ____________

VITAL SIGNS / PHYSICAL EXAM

Subject ID: |__|__|__|-|__|__|__|__|__| SUBJID   Visit Date: |__|__|-|__|__|__|-|__|__|__|__| VISITDT   Visit: |______| VISIT

1. Vital Signs

Height (cm)	Weight (kg)	Temperature °C ROUTE ROUTE. ◯ 1. Oral ◯ 2. Axillary	Sitting Measurements			
			Pulse (beats/min)	Respirations (breaths/min)	Systolic BP (mmHg)	Diastolic BP (mmHg)
HT	WT	TEMP.	PULSE	RESP	SYSBP	DIABP

2. Physical Characteristics- Circumferences

2a. MUA (mid upper arm) |__MUA__| cm   2b. Waist |WAIST__| cm   2c. Hip |HIP____| cm

3. Chest X-ray performed? ◯ 1. Yes* ◯ 0. No CHXRAY YESNO.

*If Yes, 3a. Chest X-ray result: ◯ 0. Normal ◯ 1. Abnormal XRESULT NORMAB.

3b. Why performed? ◯ 1. For clinical symptoms ◯ 2. For screening WHYXRAY WHYXRAY.

4. Physical Exam ☐ Not Done PE_ND

Body System	Normal/Abnormal/Not Done NORMABND.	Abnormal Findings (*Mark all that apply*)
General GENERAL	◯ 0. Normal ◯ 1. Abnormal ◯ 6. Not Done	☐ a. Generalized wasting GENER_A ☐ b. Lipoatrophy GENER_B ☐ c. Lipoaccumulation GENER_C
Lymph Nodes LYMPH	◯ 0. Normal ◯ 1. Abnormal ◯ 6. Not Done	☐ a. Generalized lymphadenopathy LYMPH_A ☐ b. Axillary LAD LYMPH_B ☐ c. Cervical LAD LYMPH_C ☐ d. Inguinal LAD LYMPH_D
HEENT / Mucocutaneous HEENT	◯ 0. Normal ◯ 1. Abnormal ◯ 6. Not Done	☐ a. Icterus HEENT_A ☐ b. Rash HEENT_B ☐ c. Jaundice HEENT_C ☐ d. KS-like lesions HEENT_D ☐ e. Oral Hairy Leukoplakia HEENT_E ☐ f. Thrush HEENT_F
Chest CHEST	◯ 0. Normal ◯ 1. Abnormal ◯ 6. Not Done	☐ a. Decreased breath sounds CHEST_A ☐ b. Adventitial sounds (e.g. wheeze, rale) CHEST_B
Abdomen ABDOM	◯ 0. Normal ◯ 1. Abnormal ◯ 6. Not Done	☐ a. Hepatomegaly ABDOM_A ☐ b. Splenomegaly ABDOM_B
Urogenital UROGEN	◯ 0. Normal ◯ 1. Abnormal ◯ 6. Not Done	☐ a. Genital ulcer UROGEN_A
Extremities EXTREM	◯ 0. Normal ◯ 1. Abnormal ◯ 6. Not Done	☐ a. Joint swelling EXTREM_A ☐ b. Edema EXTREM_B
Neurological NEURO	◯ 0. Normal ◯ 1. Abnormal ◯ 6. Not Done	☐ a. Unstable gait NEURO_A ☐ b. Neuropathy NEURO_B ☐ c. Tremor NEURO_C
Other Body System: OTBODSYS OTBSYTXT	◯ 0. Normal ◯ 1. Abnormal ◯ 6. Not Done	*Specify*, Abnormal Finding(s): OTHABTXT

Form Completed by: FORMBY SIGNED. ________   Date: |__|__|-|__|__|__|-|__|__|__|__| FORMDT

QC/QA: ________   Data Entry: 1st ________ 2nd ________

SPECIMEN COLLECTION

Subject ID: |__|__|__|-|__|__|__|__| SUBJID **Visit Date:** |__|__|-|__|__|__|-|__|__|__|__| VISITDT **Visit:** |______| VISIT

1. Clinical Blood Draw:

   Date: |__|__|-|__|__|__|-|__|__|__|__| CBDDT Time: |__|__|__|__| hrs (24 hour clock) CBDTIME

2. When was the last time the subject had anything to eat or drink except water, plain tea, coffee or medication?

   Date: |__|__|-|__|__|__|-|__|__|__|__| LEATDT Time: |__|__|__|__| hrs (24 hour clock) LEATTIME

3. Fasting Clinical Blood Draw (*Mark Yes if blood was drawn at least 8 hours after the time in Question 2*): FAST YESNO. ◯ 1. Yes ◯ 0. No

4. When was the last dose of ARV taken by the subject? LSTDOSE

   ◯ **5. NA (HIV Negative or not on ART)**

   Last dose date: |__|__|-|__|__|__|-|__|__|__|__| LSTDSDT Time: |__|__|__|__| Hrs (24 hour clock) LSTDSTM

5. Was this ARV regimen prescribed to be taken once or twice a day?

   ◯ 1. Once ◯ 2. Twice DOSEPDAY ONCETWIC.

6. List which ARVs were taken: ***(Refer to Medication Code List)***

   ARV_A * a. |__|__|__| ARV_B * b. |__|__|__| ARV_C * c. |__|__|__|

Repository Blood Draw

◯ **Subject Refused *(End Form)*** RBD_SUBR

7. Was repository blood draw deferred or reduced? DEFER YESNO.

   ◯ 1. Yes* ◯ 0. No

   7a. ***If Yes**, why was the draw deferred or reduced?*

   (Mark all that apply)

   DEFER_A ☐ a. Pregnant DEFER_B ☐ b. Phlebotomy Failure DEFER_C ☐ c. Anemia

Form Completed by: FORMBY SIGNED.________________ Date: |__|__|-|__|__|__|-|__|__|__|__| FORMDT

QC/QA: ____________ Data Entry: 1st ____________ 2nd ____________

BLOOD CHEMISTRY

Page/Seq. #: |____| SEQUENCE

Subject ID: |__|__|__|-|__|__|__|__|__| SUBJID Visit Date: |__|__|-|__|__|__|-|__|__|__|__| VISITDT Visit: |____| VISIT

Lab Code: |__|__| LABCODEA LABCODE. Draw Date: DRAW1DT |__|__|-|__|__|__|-|__|__|__|__|	1. Glucose	|GLUCOSE.__|	GLU_UN MGDL. ◯ 1. mmol/L ◯ 2. mg/dL
**Basic Chemistry:** Lab Code: |__|__| LABCODEB Draw Date: DRAW2DT |__|__|-|__|__|__|-|__|__|__|__|	CRET_LGE LESEQGR **2. Creatinine** Labcd2-7	|CREAT.__|	CRE_UN MGDL_U. ◯ 1. mmol/L ◯ 2. mg/dL
	3. Blood Urea Nitrogen (BUN)	|BUN.__|	BUN_UN ◯ 1. mmol/L ◯ 2. mg/dL
	4. Sodium	|SODIUM.__|	SOD_UN MEQL. ◯ 1. mmol/L ◯ 2. meq/L
	5. Potassium	|POTASS.__|	POT_UN ◯ 1. mmol/L ◯ 2. meq/L
	6. Chloride	|CHLORIDE.__|	CHL_UN ◯ 1. mmol/L ◯ 2. meq/L
	7. Carbon Dioxide (CO2)	|CO2.__|	CO2_UN ◯ 1. mmol/L ◯ 2. meq/L
**Liver Panel:** LABCODEC Lab Code: |__|__| Draw Date: DRAW3DT |__|__|-|__|__|__|-|__|__|__|__|	8. Albumin Labcd8-13	|ALBUMIN.__|	ALBI_UN GDL ◯ 1. g/L ◯ 2. g/dL
	9. Alkaline Phosphatase (ALK PHOS)	|ALKPHOS.__|	U/L
	10. Aspartate Transaminase (AST/SGOT)	|ASTSGOT.__|	U/L
	11. **Alanine Amino Transferase (ALT/SGPT)**	|ALTSGPT.__|	U/L
	12. Total Bilirubin	|TBILI.__|	TBILI_UN MGDL. ◯ 1. mmol/L ◯ 2. mg/dL
	13. Direct Bilirubin	|DBILI.__|	DBILI_UN ◯ 1. mmol/L ◯ 2. mg/dL
**Lipids:** Lab Code: |__|__| LABCODED Draw Date: DRAW4DT |__|__|-|__|__|__|-|__|__|__|__|	Labcd14-17 14. **Cholesterol Total**	|CHOLES.__|	CHO_UN ◯ 1. mmol/L ◯ 2. mg/dL
	15. Cholesterol HDL	|HDL.__|	HDL_UN ◯ 1. mmol/L ◯ 2. mg/dL
	16. Cholesterol LDL	|LDL.__|	LDL_UN ◯ 1. mmol/L ◯ 2. mg/dL
	17. Triglycerides LESEQGR Tbil_lge	|TRIGLY.__|	TRI_UN ◯ 1. mmol/L ◯ 2. mg/dL
Lab Code: |__|__| LABCODEE Draw Date: DRAW5DT |__|__|-|__|__|__|-|__|__|__|__|	**18. Pregnancy Test (Beta Hcg):**	PREGTEST NEGPOSNA.	◯ 0. Negative ◯ 1. Positive ◯ 5. NA
	19. Pregnancy Test method:	PREGMETH PREGMETH.	◯ 1. Urine ◯ 2. Blood ◯ 5. NA

The Assays listed below are not part of the Schedule of Events, but results should be recorded if obtained as part of subject's clinical care

Lab Code: |__|__| LABCODEF Draw Date: DRAW6DT |__|__|-|__|__|__|-|__|__|__|__|	20. Calcium Labcd20-27	|CALCIUM.__|	CAL_UN MGDL. ◯ 1. mmol/L ◯ 2. mg/dL
	21. Phosphate/Phosphorus	|PHOSPHAT.__|	PHO_UN ◯ 1. mmol/L ◯ 2. mg/dL
	22. Total Protein	|PROTEIN.__|	g/dL
	23. Lactate Dehydrogenase (LDH)	|LDH.__|	U/L
	24. Hgb A1C	|HGBA1C.__|	%
	25. Thyroid Stimulating Hormone (TSH)	|TSH.__|	mIU/L
	26. C-Reactive Protein	|C_REACT.__|	mg/dL
	27. G6PD	G6PD NORMDFNA.	◯ 0. Normal ◯ 1. Deficient ◯ 5. NA

Form Completed by: FORMBY SIGNED. ____________ Date: |__|__|-|__|__|__|-|__|__|__|__| FORMDT

QC/QA: ____________ Data Entry: 1st ____________ 2nd ____________

HEMATOLOGY

Page/Seq. #: |____| SEQUENCE

Subject ID: |__|__|__|-|__|__|__|__| SUBJID **Visit Date:** |__|__|-|__|__|__|-|__|__|__|__| VISITDT **Visit:** |____| VISIT

Lab Code: |__|__| LABCODE LABCODE.

Draw Date: |__|__|-|__|__|__|-|__|__|__|__| DRAWDT

Automated (Machine Differential) — **Manual** (*Mark if so*)

	Value	Units	Manual
1. WBC Count	|____.__| WBC	10^3 cells/ul	
**2. Hemoglobin**	|____.__| HEMOG	g/dl	
3. Mean Corpuscular Volume	|____.__| MCV	fl	
4. Platelet Count	|____.__| PLATELET	10^3 cells/ul	
5. Neutrophil %	|____.__| NEUTRO	%	☐ NEUT_CK
6. Lymphocyte %	|____.__| LYMPH	%	☐ LYMPH_CK
7. Monocyte %	|____.__| MONOCYTE	%	☐ MONO_CK
8. Eosinophil %	|____.__| EOSINO	%	☐ EOS_CK
9. Basophil %	|____.__| BASOPHIL	%	☐ BASO_CK

The Assay listed below is not part of the Schedule of Events, but results should be recorded if obtained as part of subject's clinical care

10. Erythrocyte Sedimentation Rate (ESR) |______| ESR mm/hr

Form Completed by: FORMBY SIGNED. ______________ Date: |__|__|-|__|__|__|-|__|__|__|__| FORMDT

URINALYSIS

Page/Seq. #: |___| SEQUENCE

Subject ID: SUBJID |__|__|__|-|__|__|__|__|__| Visit Date: VISITDT |__|__|-|__|__|__|-|__|__|__|__| Visit: VISIT |___|

The Assays listed below are not part of the Schedule of Events, but results should be recorded if obtained as part of subject's clinical care

Lab Code: LABCODE LABCODE. |__|__| Draw Date: DRAWDT |__|__|-|__|__|__|-|__|__|__|__|

1. Color COLOR COLOR. |__| (0= Yellow; 1= other than Yellow)
2. Appearance APPEAR APPEAR. |__| (0= Clear; 1= other than Clear)
3. Glucose GLUCOSE ZEROFOUR. |__| (0= Neg or Normal; 1= Trace, 1+ 100 or 250; 2= 2+ or 500; 3= 3+ or 1000; 4= 4+ or ≥ 2000)
4. Bilirubin BILIRUB ZEROTHRE. |__| (0= Neg or Normal; 1 = Trace, 1+ or small; 2= 2+ or moderate; 3= 3+ or Large)
5. Ketones KETONES KETONES. |__| (0= Neg or Normal; 1= Trace, 5, 1+, Small, 15; 2= 2+, Moderate, 40; 3= 3+, Large, 80; 4= 4+, 160)
6. SPGR SPGR_LGE LESEQGR. ☐ (<, =, or >) |___.______| SPGR
7. Blood BLOOD ZEROTHRE. |__| (0= Neg or Normal; 1= Trace, 1+ or small; 2= 2+ or moderate; 3= 3+ or Large)
8. pH PH |__.__|
9. Protein PROTEIN PROTEIN. |__| (0= Neg or Normal; 1=Trace, 1+ or 30; 2=2+ or 100; 3=3+ or 300; 4=4+ or >300)
10. Urobilinogen UROB_LGE LESEQGR. ☐ (<, =, or >) |__| UROB
11. Nitrite NITRITE NITRITE. |__| (0= Neg or Normal; 1= Positive)
12. Leukocyte Esterase LEUKO ZEROTHRE. |__| (0= Neg or Normal; 1= Trace, 1+ or small; 2= 2+ or moderate; 3= 3+ or Large)
13. RBC UA RBCUA ZEROFIVE. |__| /HPF
14. WBC UA WBCUA ZEROFIVE. |__| /HPF

Note:
For 13. & 14. use the following codes:

0=Neg, 0, <1	3=11-29
1=1-4	4=30-50
2=5-10	5=>50, TNTC, PACKED/HPF

15. Micro-albumin ALBUMIN |______.___| mg/L
16. Protein-Creatinine Ratio PCRATIO |______.___| PCTIME PCTIME. ◯ 1. 24 Hr ◯ 2. Random

(Complete 17. & 18. only if 16. Protein-Creatinine Ratio not provided)

17. Protein PROTEINC |______.___| mg/dL
18. Creatinine CREAT |______.___| mg/dL

Form Completed by: FORMBY SIGNED. ____________________ Date: FORMDT |__|__|-|__|__|__|-|__|__|__|__|

QC/QA: __________ Data Entry: 1st __________ 2nd __________

SEQUENCED

LYMPHOCYTE SUBSET PROFILE

Page #: |____|

Subject ID: |__|__|__|-|__|__|__|__| SUBJID   Visit Date: |__|__|-|__|__|__|-|__|__|__|__| VISITDT   Visit: |____| VISIT

Seq. #: |____| SEQUENCE

Draw Period: ◯ 1. At study visit ◯ 2. Outside of study visit DRAWPER DRAWPER.

Lab Code: |__|__| LABCODE LABCODE.   Draw Date: |__|__|-|__|__|__|-|__|__|__|__| DRAWDT

1. WBC WBC	|____________| /mm³	
2. Lymphs LYMPH	|____________| /mm³	LYMPH_P |_____._| %
3. CD3+ CD3_N	|_____._| m³	
**4. CD3+CD4+** CD3_4_N	|____________| /mm³	CD3_4_P |_____._| %
5. CD3+CD8+ CD3_8_N	|____________| /mm³	CD3_8_P |_____._| %
6. % CD19+		CD19_P |_____._| %
7. % CD3-CD56+		CD3_56_P |_____._| %

Seq. #: |____|

Draw Period: ◯ 1. At study visit ◯ 2. Outside of study visit

Lab Code: |__|__| Draw Date: |__|__|-|__|__|__|-|__|__|__|__|

1. WBC	|____________| /mm³	
2. Lymphs	|____________| /mm³	|_____._| %
3. CD3+	|_____._| m³	
**4. CD3+CD4+**	|____________| /mm³	|_____._| %
5. CD3+CD8+	|____________| /mm³	|_____._| %
6. % CD19+		|_____._| %
7. % CD3-CD56+		|_____._| %

Form Completed by: FORMBY SIGNED. ______________________   Date: |__|__|-|__|__|__|-|__|__|__|__| FORMDT

QC/QA: ____________   Data Entry: 1st ____________ 2nd ____________

ADDITIONAL MICROBIOLOGY

Page/Seq. #: SEQUENCE |__|

Subject ID: SUBJID |__|__|__|-|__|__|__|__|__| Visit Date: VISITDT |__|__|-|__|__|__|-|__|__|__|__| Visit: VISIT |__|

1. TB Xpert: TBXPERT NEGPOSND. Lab Code: LABCODE LABCODE. |__|__| Date: TBXDT |__|__|-|__|__|__|-|__|__|__|__| Specimen Code[1]: TBXSPCM SPECIMEN. |__|__|__|

0. Negative

1. Positive → ***If 1. Positive***, drug resistance to Rifampin? RESIS YESNO. 1. Yes 0. No

6. Not Done → ***If 6. Not Done***, 1. Stored 2. No sputum produced TBXPRTND TBXPRTND.

The Assays listed below are not part of the Schedule of Events, but results should be recorded if obtained as part of subject's clinical care

2. Stool Exams for ova and parasites: STOOLEXA NEGPOS.
0. Negative 1. Positive
Date: SEDT |__|__|-|__|__|__|-|__|__|__|__|
Code[2] (1) SE_1CD* |__|__|__| Code[2] (2) SE_2CD* |__|__|__| Code[2] (3) SE_3CD* |__|__|__|
Other, *Specify*: SE_TXT ____________

3. Stool Culture: STOOLCUL NEGPOS.
0. Negative 1. Positive
Date: SCDT |__|__|-|__|__|__|-|__|__|__|__|
Code[2] (1) SC_1CD* |__|__|__| Code[2] (2) SC_2CD* |__|__|__| Code[2] (3) SC_3CD* |__|__|__|
Other, *Specify*: SC_TXT ____________

4. Blood Culture: BLOODCUL NEGPOS.
0. Negative 1. Positive
Date: BCDT |__|__|-|__|__|__|-|__|__|__|__|
Code[2] (1) BC_1CD* |__|__|__| Code[2] (2) BC_2CD* |__|__|__| Code[2] (3) BC_3CD* |__|__|__|
Other, *Specify*: BC_TXT ____________

5. Other Body Fluid Culture: FLUIDCUL NEGPOS.
0. Negative 1. Positive
Date: BFCDT |__|__|-|__|__|__|-|__|__|__|__|
Code[2] (1) BFC_1CD* |__|__|__| Code[2] (2) BFC_2CD* |__|__|__| Code[2] (3) BFC_3CD* |__|__|__|
Specimen Code[1]: BFCSPCM SPECIMEN. |__|__|__| Other, *Specify*: BFC_TXT ____________

6. Chlamydia: CHLAMYDI NEGPOS. 0. Negative 1. Positive Date: CHLMDT |__|__|-|__|__|__|-|__|__|__|__|
Type of Test: CHLMTEST CHLMTEST. 1. NAAT 2. Rapid Diagnostic (RDT) 90. Other, *Specify*: CHLM_TXT ____________

7. Gonorrhea: GONORRHE NEGPOS. 0. Negative 1. Positive Date: GONDT |__|__|-|__|__|__|-|__|__|__|__|
Type of Test: GONTEST GONTEST. 1. NAAT 2. Culture 90. Other, *Specify*: GON_TXT ____________

8. HPV (cervical specimen): HPV NEGPOS. 0. Negative
1. Positive → ***If Positive***, Subtype: HPV_TXT ____________

9. Mycobacterial specimen type**: MYCOBTP SPECIMEN. |__|__|__|

9a. Mycobacterial smear: Date: MYCSMDT |__|__|-|__|__|__|-|__|__|__|__|
MYCOBSM NEGPOS. 0. Negative 1. Positive

9b. Mycobacterial culture Date: MYCCUDT |__|__|-|__|__|__|-|__|__|__|__|
MYCOBCUL MYCOBCUL. 0. Negative 1. Positive, MTB* 2. Positive, MAC* 3. Positive, Other*, *Specify*: MYC_TXT ____________

If Positive, MTB, MAC, or Other,** Sensitivity Testing Results ***(Mark all resistance that applies):
ISONIAZ Isoniazid PYRAZIN Pyrazinamide RIFAMPIN Rifampin ETHAM Ethambutol OTHER Other, *Specify*: PMYC_TXT ____________

[1]Refer to Specimen Code List; [2]Refer to Organism Code List

Form Completed by: FORMBY SIGNED. ____________ Date: FORMDT |__|__|-|__|__|__|-|__|__|__|__|

QC/QA: ____________ Data Entry: 1st ____________ 2nd ____________

SEQUENCED

VIRAL LOAD (HIV Positive)

Page #: |____|

Subject ID: |__|__|__|-|__|__|__|__| SUBJID Visit Date: |__|__|-|__|__|__|-|__|__|__|__| VISITDT Visit: |____| VISIT

○ NA (HIV Negative) NAHIVNEG

SEQUENCE DRAWPER DRAWPER.

Seq. #: |____| Draw Period: ○ 1. At study visit ○ 2. Outside of study visit

Lab Code: |__|__| LABCODE LABCODE.

Draw Date: |__|__|-|__|__|__|-|__|__|__|__| DRAWDT

Circle One: < = > |________| copies/mL VLCOPY

VL_CIRC LESEQGR. NODETECT ○ Not Detected

Seq. #: |____| Draw Period: ○ 1. At study visit ○ 2. Outside of study visit

Lab Code: |__|__|

Draw Date: |__|__|-|__|__|__|-|__|__|__|__|

Circle One: < = > |________| copies/mL

○ Not Detected

Seq. #: |____| Draw Period: ○ 1. At study visit ○ 2. Outside of study visit

Lab Code: |__|__|

Draw Date: |__|__|-|__|__|__|-|__|__|__|__|

Circle One: < = > |________| copies/mL

○ Not Detected

Seq. #: |____| Draw Period: ○ 1. At study visit ○ 2. Outside of study visit

Lab Code: |__|__|

Draw Date: |__|__|-|__|__|__|-|__|__|__|__|

Circle One: < = > |________| copies/mL

○ Not Detected

Seq. #: |____| Draw Period: ○ 1. At study visit ○ 2. Outside of study visit

Lab Code: |__|__|

Draw Date: |__|__|-|__|__|__|-|__|__|__|__|

Circle One: < = > |________| copies/mL

○ Not Detected

Seq. #: |____| Draw Period: ○ 1. At study visit ○ 2. Outside of study visit

Lab Code: |__|__|

Draw Date: |__|__|-|__|__|__|-|__|__|__|__|

Circle One: < = > |________| copies/mL

○ Not Detected

Form Completed by: FORMBY SIGNED. ________________ Date: |__|__|-|__|__|__|-|__|__|__|__| FORMDT

QC/QA: ________ Data Entry: 1st ________ 2nd ________

SEQUENCED

SEROLOGY (HIV Positive & Negative)

Page #: |____|

Subject ID: |__|__|__|-|__|__|__|__|__| SUBJID   Visit Date: |__|__|-|__|__|__|-|__|__|__|__| VISITDT   Visit: |____| VISIT

Seq. #: SEQUENCE A. |____| B. |____| C. |____|

Draw Date: DRAWDT
A. |__|__|-|__|__|__|-|__|__|__|__| B. |__|__|-|__|__|__|-|__|__|__|__| C. |__|__|-|__|__|__|-|__|__|__|__|

LABCODE
LABCODE.

0= Non-Reactive 1= Reactive 2= Indeterminate → LABCD1-18	A LAB CODE: |__|__| (0, 1, or 2)	B LAB CODE: |__|__| (0, 1, or 2)	C LAB CODE: |__|__| (0, 1, or 2)
**1. Hepatitis C virus AB (AntiHCV)**........ HEPCAB	|__| NOREIND.	|__|	|__|
2. Hepatitis C confirmatory test........ HEPC	|__|	|__|	|__|
**3. Syphilis screen:** ◯ 1. RPR ◯ 2. VDRL SYPHSCRN SCRNRPR RPRVDRL.	|__|	|__|	|__|
4. Syphilis (Titer):........	SYPHTITE 1: |__|__|__|__|__|	1: |__|__|__|__|__|	1: |__|__|__|__|__|
5. FTA-ABS........ FTAABS	|__| NOREIND.	|__|	|__|
6. MHA-TP/TP-PA........ MHATP	|__|	|__|	|__|
**7. Hepatitis B surface Antigen**........ HEPBSA	|__|	|__|	|__|
8. Hepatitis B surface Antigen confirmatory test HEPBSAC	|__|	|__|	|__|
9. Hepatitis B e Antigen........ HEPBEA	|__|	|__|	|__|
10. Hepatitis B Core Antibody........ HEPBCA	|__|	|__|	|__|
**11. Quantiferon TB ELISA**........ TBELISA	|__|	|__|	|__|
**12. Serum Cryptococcal Antigen**........ SERUMCRY	|__|	|__|	|__|
**The Assays listed below are not part of the Schedule of Events, but results should be recorded if obtained as part of subject's clinical care**			
13. Herpes Simplex Virus I........ VIRUS_I	|__|	|__|	|__|
14. Herpes Simplex Virus II........ VIRUS_II	|__|	|__|	|__|
15. Hepatitis A Antibody........ HEPAA	|__|	|__|	|__|
16. CSF Cryptococcal Antigen........ CSFCA	|__|	|__|	|__|
17. Toxoplasma Serology........ TOXSERO	|__|	|__|	|__|
18. Cytomegalovirus (CMV)........ CMV	|__|	|__|	|__|

Form Completed by: FORMBY SIGNED. ____________________   Date: |__|__|-|__|__|__|-|__|__|__|__| FORMDT

QC/QA: ____________   **Data Entry: 1st** ____________ **2nd** ____________

SEROLOGY (HIV Negative only)

Page/Seq. #: |___| SEQUENCE

Subject ID: |__|__|__|-|__|__|__|__|__| SUBJID Visit Date: |__|__|-|__|__|__|-|__|__|__|__| VISITDT Visit: |___| VISIT

○ NA (HIV Positive) NAHIVPOS

Lab Code (for Rapid Diagnostic Test(s)): |__|__| LABCODEA LABCODE.

1. HIV Rapid Diagnostic Test (1):

Date of Test: |__|__|-|__|__|__|-|__|__|__|__| RDT1DT

○ 0. Negative ○ 1. Positive HIV_1RDT NEGPOS.

Type: RDT1TYPE RDTTYPE.
- ○ 1. Determine
- ○ 2. Unigold
- ○ 3. Bioline
- ○ 4. Statpak
- ○ 90. Other, *Specify*: OTH1_TXT ______

2. HIV Rapid Diagnostic Test (2):

Date of Test: |__|__|-|__|__|__|-|__|__|__|__| RDT2DT

○ 0. Negative ○ 1. Positive HIV_2RDT NEGPOS.

Type: RDT2TYPE RDTTYPE.
- ○ 1. Determine
- ○ 2. Unigold
- ○ 3. Bioline
- ○ 4. Statpak
- ○ 90. Other, *Specify*: OTH2_TXT ______

3. HIV Rapid Diagnostic Test (3):

Date of Test: |__|__|-|__|__|__|-|__|__|__|__| RDT3DT

○ 0. Negative ○ 1. Positive HIV_3RDT NEGPOS.

Type: RDT3TYPE RDTTYPE.
- ○ 1. Determine
- ○ 2. Unigold
- ○ 3. Bioline
- ○ 4. Statpak
- ○ 90. Other, *Specify*: OTH3_TXT ______

Lab Code (for ELISA & Western Blot Tests): |__|__| LABCODEB LABCODE.

4. HIV ELISA Test:

Date of Test: |__|__|-|__|__|__|-|__|__|__|__| ELISADT

ELISRSLT NOREIND.
- ○ 0. Nonreactive
- ○ 1. Reactive
- ○ 2. Indeterminate

5. HIV Western Blot Test:

Date of Test: |__|__|-|__|__|__|-|__|__|__|__| BLOTDT

BLOTRSLT NEGPOSIN.
- ○ 0. Negative
- ○ 1. Positive
- ○ 2. Indeterminate

Form Completed by: FORMBY SIGNED. ______ Date: |__|__|-|__|__|__|-|__|__|__|__| FORMDT

QC/QA: ______ Data Entry: 1st ______ 2nd ______

RESISTANCE TEST

Page/Seq. #: |___| SEQUENCE

Subject ID: |_|_|_|-|_|_|_|_| SUBJID Visit Date: |_|_|-|_|_|_|-|_|_|_|_| VISITDT Visit: |___| VISIT

○ NA (HIV Negative) NAHIVNEG

Type of Sample: SAMPLETP PLASSERU Lab Code: |_|_| LABCODE LABCODE.

○ 1. Plasma ○ 2. Serum

Draw Date: |_|_|-|_|_|_|-|_|_|_|_| DRAWDT

HIV Subtype/clade: HIVCLADE HIVCLADE.

○ 1. A ○ 4. D ○ 7. G ○ 10. J
○ 2. B ○ 5. E ○ 8. H ○ 11. K
○ 3. C ○ 6. F ○ 9. I ○ 12. CRF01_AE
○ 13. CRF02_AG
○ 14. CRF09_CPX
○ 15. Other Circulating, Recombinant strain: CLADETXT ____________

GENOTYPES:

Major NRTI mutations:	Major NNRTI mutations:	Major PI mutations:	Major Integrase mutations:
☐ M184V/I M184V	☐ K103N K103N	☐ PI mutation (1), *Specify*: PIA PI1TXT ______	☐ Integrase mutation (1), *Specify*: INTA INT1TXT ______
☐ K65R K65R	☐ Y181C Y181C	☐ PI mutation (2), *Specify*: PIB PI2TXT ______	☐ Integrase mutation (2), *Specify*: INTB INT2TXT ______
☐ M41L M41L	☐ Y188L Y188L	☐ PI mutation (3), *Specify*: PIC PI3TXT ______	☐ Integrase mutation (3), *Specify*: INTC INT3TXT ______
☐ T69D T69D	☐ Y188C Y188C	☐ PI mutation (4), *Specify*: PID PI4TXT ______	
☐ T215Y T215Y	☐ Y188H Y188H	☐ PI mutation (5), *Specify*: PIE PI5TXT ______	
☐ T215S T215S	☐ V106A V106A	☐ PI mutation (6), *Specify*: PIF PI6TXT ______	
☐ K219E K219E	☐ V106M V106M		
☐ K219D K219D	☐ K101E K101E		
☐ L210W L210W	☐ G190A G190A		
☐ L74I L74I	☐ G190S G190S		
☐ L74V L74V	☐ L100I L100L		
☐ K70R K70R	☐ Other (1), *Specify*: NOTHA NOTH1TXT ______		
☐ D67N D67N	☐ Other (2), *Specify*: NOTHB NOTH2TXT ______		
☐ Other (1), *Specify*: OTHA OTH1TXT ______	☐ Other (3), *Specify*: NOTHC NOTH3TXT ______		
☐ Other (2), *Specify*: OTHB OTH2TXT ______	☐ Other (4), *Specify*: NOTHD NOTH4TXT ______		
☐ Other (3), *Specify*: OTHC OTH3TXT ______			
☐ Other (4), *Specify*: OTHD OTH4TXT ______			

PHENOTYPES: List codes of ARV drugs classified as resistant ***(Refer to Medication Code List)***

	Code*	Fold Change		Code*	Fold Change		Code*	Fold Change		Code*	Fold Change
1.	CODE_A |_|_|_|_|	FOLD_A |_|_|_|_|	4.	CODE_D |_|_|_|_|	FOLD_D |_|_|_|_|	7.	CODE_G |_|_|_|_|	FOLD_G |_|_|_|_|	10.	CODE_J |_|_|_|_|	FOLD_J |_|_|_|_|
2.	CODE_B |_|_|_|_|	FOLD_B |_|_|_|_|	5.	CODE_E |_|_|_|_|	FOLD_E |_|_|_|_|	8.	CODE_H |_|_|_|_|	FOLD_H |_|_|_|_|	11.	CODE_K |_|_|_|_|	FOLD_K |_|_|_|_|
3.	CODE_C |_|_|_|_|	FOLD_C |_|_|_|_|	6.	CODE_F |_|_|_|_|	FOLD_F |_|_|_|_|	9.	CODE_I |_|_|_|_|	FOLD_I |_|_|_|_|	12.	CODE_L |_|_|_|_|	FOLD_L |_|_|_|_|

Form Completed by: FORMBY SIGNED. ______________ Date: |_|_|-|_|_|_|-|_|_|_|_| FORMDT

QC/QA: ______________ Data Entry: 1st ______________ 2nd ______________

COGNITIVE EVALUATION (page 1 of 2)

Subject ID: |__|__|__|-|__|__|__|__|__| SUBJID **Visit Date:** |__|__|-|__|__|__|-|__|__|__|__| VISITDT **Visit:** |______| VISIT

The following section is a summary of the clinical team's final impression of the subject's level of function as it relates to cognition. It should be based on all available information and sources including the subject evaluation, information from friends and family members, and recent information from clinical encounters.

Language used during testing: LANGUAGE LANGUAGE.
◯ 1. English ◯ 2. Kiswahili ◯ 3. Luo ◯ 4. Luganda ◯ 5. Pidgin English ◯ 90. Other, *Specify:* OTH_1TXT

1. Please indicate the level of difficulty the subject currently experiences in facets of daily living, using a scale of: 0 = Normal, 1 = Mild, 2 = Moderate/Severe ***(Indicate only one level per facet on each row)***

NORMIMOD.

	**Normal**	**Mild**	**Moderate/Severe**
	**0**	**1**	**2**
**a. Memory** FACET_A |__|	Normal memory or slight inconsistent forgetfulness; similar to peers and not problematic	Mild memory loss; may interfere with daily activities	At least moderate memory loss; clearly effects daily function and may lead to inability to carry out daily activities or work
**b. Orientation** FACET_B |__|	Fully oriented	Fully oriented but may have difficulty with time relationships and occasionally disoriented	Moderate/severe difficulty with time orientation; may get lost; usually disoriented to time or to place
**c. Higher cognitive thinking** FACET_C |__|	No problem solving or judgment issues; able to manage all personal business & financial issues; normal judgment	Mild impairment in solving problems, judgment or more complex thinking	At lest moderate difficulty in handling problems; social judgment may be impaired
**d. Activities outside of the home** FACET_D |__|	Independent function at usual level in job, shopping, subject & social groups	Mild impairment that is due to cognition and is not due to physical limitations alone	Unable to function independently at these activities that is due to cognition and is not due to physical limitations alone
**e. Activities within the home** FACET_E |__|	Normal ability to participate in life at home including hobbies	Mild limitations in life at home, hobbies that is due to cognition and is not due to physical limitations alone	At least moderate impairment of function at home that is due to cognition and is not due to physical limitations alone
**f. Personal care (Activities of Daily Living)** FACET_F |__|	Independent in personal care, (bathing toileting, transferring position, dressing, feeding)	Mild impairment in these activities of daily living that is due to cognition and is not due to physical limitations alone	At least moderate impairment in these activities of daily living that is due to cognition and is not due to physical limitations alone

2. Sources of information used to rate functions: ***(Mark all that apply)***

- ☐ a. Subject SOURCE_A
- ☐ b. Family member SOURCE_B
- ☐ c. Close friend/peer SOURCE_C
- ☐ d. Clinical history SOURCE_D
- ☐ e. Other, *Specify:* SOURCE_E OTH_2TXT ______________________

QC/QA: ____________ **Data Entry: 1st** ____________ **2nd** ____________

COGNITIVE EVALUATION (page 2 of 2)

Subject ID: SUBJID |__|__|__|-|__|__|__|__|__| Visit Date: VISITDT |__|__|-|__|__|__|-|__|__|__|__| Visit: VISIT |______|

Please consult with the Cognitive Evaluation Source Document for more information about the following tests.

I. INTERNATIONAL HIV DEMENTIA SCALE

1. Registration - Words remembered:

**Trial 1:**	TRIAL11T ☐ 1. Red	TRIAL12T ☐ 2. Dog	TRIAL13T ☐ 3. Cap	TRIAL14T ☐ 4. Beans	
**Trial 2:**	TRIAL21T ☐ 1. Red	TRIAL22T ☐ 2. Dog	TRIAL23T ☐ 3. Cap	TRIAL24T ☐ 4. Beans	TRIAL_NA ◯ ***7. N/A – (4 of 4 remembered in Trial 1)***

2. Motor Speed - Number of taps in 5 seconds: NUMTAPS |___|___| *(number of taps)* TAPSPTS |___| points awarded *(max of 4)*

3. Psychomotor Speed - Number of sequences correctly performed in 10 seconds: COR_SEQS |______|

4. Memory Recall – Points for words remembered: NUMWORDS |___|.|___| *(max of 4.0, 1 each, 0.5 each if prompted)*

VALID_1T VALIDITY.
Test I. Validity: ◯ 1. Valid ◯ 0. Invalid, *Specify:* VAL1_TXT ____________

II. WHO-UCLA AUDITORY VERBAL LEARNING TEST

List: ◯ 1. Primary ◯ 2. Secondary AVLTLIST PRIMSEC.

Trial	Correct Responses	Intrusions	Repetitions
I.	T1_RESP |______|	T1_INT |______|	T1_REPET |______|
II.	T2_RESP |______|	T2_INT |______|	T2_REPET |______|
III.	T3_RESP |______|	T3_INT |______|	T3_REPET |______|
IV.	T4_RESP |______|	T4_INT |______|	T4_REPET |______|
V.	T5_RESP |______|	T5_INT |______|	T5_REPET |______|

Trial	Correct Responses	Intrusions	Repetitions
VI.	T6_RESP |______|	T6_INT |______|	T6_REPET |______|
VII.	T7_RESP |______|	T7_INT |______|	T7_REPET |______|
VIII.	T8_RESP |______|	T8_INT |______|	T8_REPET |______|
IX.	Hits T9_HITS |______|	False Positives T9_FPOS |______|	

VALID_2T VALIDITY.
Test II. Validity: ◯ 1. Valid ◯ 0. Invalid, *Specify:* VAL2_TXT ____________

III. GROOVED PEGBOARD TEST

DOM_HAND

**Dominant Hand:** Right Left (Circle one)	**Non-dominant Hand:**
Time: DOM_MIN |___|' DOM_SEC |___|___|" ***(max of 5'00")*** *(minutes' seconds")*	Time: NDM_MIN |___|' NDM_SEC |___|___|" ***(max of 5'00")*** *(minutes' seconds")*
Drops: DOM_DROP |_____| Correct: DOM_CORR |_____|	Drops: NDM_DROP |_____| Correct: NDM_CORR |_____|

VALID_3T VALIDITY.
Test III. Validity: ◯ 1. Valid ◯ 0. Invalid, *Specify:* VAL3_TXT ____________

IV. ACTION FLUENCY TEST

Correct Responses: FT_CORR |_____| Rule Violations: FT_VIOLA |_____| Repetitions: FT_REPET |_____|

VALID_4T VALIDITY.
Test IV. Validity: ◯ 1. Valid ◯ 0. Invalid, *Specify:* VAL4_TXT ____________

V. TRAILS A TEST

Time: TT_MIN |___|' TT_SEC |___|___|" *(minutes' seconds")* Total Number of Correct Lines: TT_LINE |_____| Errors: TT_ERROR |_____| Prompts: TT_PROMP |_____|

VALID_5T VALIDITY.
Test V. Validity: ◯ 1. Valid ◯ 0. Invalid, *Specify:* VAL5_TXT ____________

Form Completed by: **FORMBY** SIGNED. **FRMBYTXT** ____________ Date: FORMDT |__|__|-|__|__|__|-|__|__|__|__|

QC/QA: ____________ Data Entry: 1st ____________ 2nd ____________

SEQUENCED

CURRENT MEDICAL CONDITIONS

Page #: |____|

Subject ID: |__|__|__|-|__|__|__|__| SUBJID   Visit Date: |__|__|-|__|__|__|-|__|__|__|__| VISITDT   Visit: |____| VISIT

○ Mark if None NONE

Update ongoing conditions from previous visit and add new medical conditions

Seq. #:	Codes			Start Date	Stop Date	Mark if Ongoing
	Diagnosis[1]	Diagnosis Method[2]	Treatment Outcome[3]	STARTDT	STOPDT	ONGO
SEQUENCE |____|	DX* |__|__|__|__|.|__|__|__|	DX_METH |____| DXMETHOD.	OUT_TX |__|__| TXOUTCM.	|__|__|-|__|__|__|-|__|__|__|__|	|__|__|-|__|__|__|-|__|__|__|__|	○
|____|	|__|__|__|__|.|__|__|__|	|____|	|__|__|	|__|__|-|__|__|__|-|__|__|__|__|	|__|__|-|__|__|__|-|__|__|__|__|	○
|____|	|__|__|__|__|.|__|__|__|	|____|	|__|__|	|__|__|-|__|__|__|-|__|__|__|__|	|__|__|-|__|__|__|-|__|__|__|__|	○
|____|	|__|__|__|__|.|__|__|__|	|____|	|__|__|	|__|__|-|__|__|__|-|__|__|__|__|	|__|__|-|__|__|__|-|__|__|__|__|	○
|____|	|__|__|__|__|.|__|__|__|	|____|	|__|__|	|__|__|-|__|__|__|-|__|__|__|__|	|__|__|-|__|__|__|-|__|__|__|__|	○
|____|	|__|__|__|__|.|__|__|__|	|____|	|__|__|	|__|__|-|__|__|__|-|__|__|__|__|	|__|__|-|__|__|__|-|__|__|__|__|	○
|____|	|__|__|__|__|.|__|__|__|	|____|	|__|__|	|__|__|-|__|__|__|-|__|__|__|__|	|__|__|-|__|__|__|-|__|__|__|__|	○
|____|	|__|__|__|__|.|__|__|__|	|____|	|__|__|	|__|__|-|__|__|__|-|__|__|__|__|	|__|__|-|__|__|__|-|__|__|__|__|	○
|____|	|__|__|__|__|.|__|__|__|	|____|	|__|__|	|__|__|-|__|__|__|-|__|__|__|__|	|__|__|-|__|__|__|-|__|__|__|__|	○
|____|	|__|__|__|__|.|__|__|__|	|____|	|__|__|	|__|__|-|__|__|__|-|__|__|__|__|	|__|__|-|__|__|__|-|__|__|__|__|	○
|____|	|__|__|__|__|.|__|__|__|	|____|	|__|__|	|__|__|-|__|__|__|-|__|__|__|__|	|__|__|-|__|__|__|-|__|__|__|__|	○
|____|	|__|__|__|__|.|__|__|__|	|____|	|__|__|	|__|__|-|__|__|__|-|__|__|__|__|	|__|__|-|__|__|__|-|__|__|__|__|	○

Comments

CMC_TXT1 ____

____

____

____

[1]*Refer to Diagnosis Code List;* [2]*Refer to Diagnosis Method Code List;* [3]*Refer to Treatment Outcome Code List*

Form Completed by: FORMBY SIGNED. ______________________   Date: |__|__|-|__|__|__|-|__|__|__|__| FORMDT

QC/QA: ____________   Data Entry: 1st ____________ 2nd ____________

ACUTE FEBRILE ILLNESS

Subject ID: |__|__|__|-|__|__|__|__| SUBJID **Visit Date:** |__|__|-|__|__|__|-|__|__|__|__| VISITDT **Visit:** |______| VISIT

Complete if clinical concern for Malaria and/or fever

Date of Examination: |__|__|-|__|__|__|-|__|__|__|__| EXAMDT

1. Temperature ≥ 99.5°F (37.5°C) or higher: ◯ 1. Yes ◯ 0. No ORALTEMP YESNO.

2. Any symptoms? ◯ 1. Yes* ◯ 0. No SYMPTOMS YESNO.

***If Yes, (*Mark all that apply*)** SYMP_A – SYMP_M

☐ a. Feverishness
☐ b. Headache
☐ c. Malaise/Fatigue
☐ d. Chills
☐ e. Arthralgias
☐ f. Myalgias
☐ g. Nausea
☐ h. Vomiting
☐ i. Diarrhea
☐ j. Abdominal pain
☐ k. Anorexia
☐ l. Chest Pain
☐ m. Low back pain
☐ z. Other, *Specify:* SYMP_Z OTH2_TXT

3. Do you sleep under a mosquito net? MOSQUITO NSANR.

◯ 0. Not at all ◯ 1. Sometimes ◯ 2. Always ◯ 8. No Response

4. In the time since your last study visit, have you taken medicine to treat malaria? MAL_TX YESNONR.

◯ 1. Yes ◯ 0. No ◯ 8. No Response

5. Malaria Smear: ◯ 0. Negative ◯ 1. Positive* ◯ 6. Not Done SMEAR NEGPOSND.

***If Positive,** 5a. ☐ a. Malariae SMEAR_A ☐ b. Falciparum SMEAR_B ☐ c. Ovale SMEAR_C ☐ d. Vivax SMEAR_D

5b. On which smears were parasites seen? ☐ a. Thick SMEARS_A ☐ b. Thin SMEARS_B

6. Rapid Diagnostic Test (RDT): RDT NPINCND.

◯ 0. Negative ◯ 2. Inconclusive
◯ 1. Positive ◯ 6. Not Done

6a. If performed, RDT Name: RDTNAME RDTNAME.

◯ 1. SD Bioline Pf ◯ 4. First Response
◯ 2. SD Bioline Pan ◯ 5. ICT
◯ 3. Carestat ◯ 90. Other, *Specify:* OTH6_TXT

7. Diagnosis given to subject[1]: |__|__|__|.|__|__| DXGIVEN*

[1] *Refer to Diagnosis Code List; if Diagnosis is Malaria, use the following codes:*
B50.0=Cerebral Malaria
B50.8=Severe Malaria, non-cerebral
B50.9=Uncomplicated Malaria

8. Treatment given (***Mark all that apply***) TX_A – TX_J

☐ a. Oral Artemesinin combination
☐ b. Oral Quinine
☐ c. Oral Chloroquine
☐ d. Oral Fansidar
☐ e. Oral Primaquine
☐ f. Oral Clindamycin
☐ g. Oral Doxycycline or Tetracycline
☐ h. IV/IM Artemether
☐ i. IV/IM Quinine
☐ j. IV/IM Artesunate
☐ z. Other, *Specify:* TX_Z OTH8_TXT

9. DBS for PCR obtained? ◯ 1. Yes ◯ 0. No DBS_PCR YESNO.

Form Completed by: FORMBY SIGNED. ________ Date: |__|__|-|__|__|__|-|__|__|__|__| FORMDT

QC/QA: ________ **Data Entry: 1st** ________ **2nd** ________

SEQUENCED HOSPITALIZATION / SERIOUS/ACUTE VISIT

Page #: |____|

Subject ID: |__|__|__|-|__|__|__|__| SUBJID Visit Date: |__|__|-|__|__|__|-|__|__|__|__| VISITDT Visit: |____| VISIT

Sequence #: SEQUENCE |____|

Date of hospital visit: HOSPVDT |__|__|-|__|__|__|-|__|__|__|__|

NOTHOSP ◯ Not Hospitalized

Date of discharge, if hospitalized: DISDT |__|__|-|__|__|__|-|__|__|__|__|

	Diagnosis[1]	Dx Method[2] DXMETHOD.	Treatment[3] TXCODE.	TX Outcome[4] TXOUTCM.	Comments
a.	DX_A* |__|__|__|__|.|__|__|__|	DXMETH_A |____|	TX_1A – TX_4A |__| |__| |__| |__|	TX_OUT_A |__|__|	A_TXT
b.	DX_B* |__|__|__|__|.|__|__|__|	DXMETH_B |____|	TX_1B – TX_4B |__| |__| |__| |__|	TX_OUT_B |__|__|	B_TXT
c.	DX_C* |__|__|__|__|.|__|__|__|	DXMETH_C |____|	TX_1C – TX_4C |__| |__| |__| |__|	TX_OUT_C |__|__|	C_TXT

Hospital Name: HOSPNAME ______________________________

Location: HOSPLOC ______________________________

Sequence #: |____|

Date of hospital visit: |__|__|-|__|__|__|-|__|__|__|__|

◯ Not Hospitalized

Date of discharge, if hospitalized: |__|__|-|__|__|__|-|__|__|__|__|

	Codes				Comments
	Diagnosis[1]	Dx Method[2]	Treatment[3]	TX Outcome[4]	
a.	|__|__|__|__|.|__|__|__|	|____|	|__| |__| |__| |__|	|__|__|	
b.	|__|__|__|__|.|__|__|__|	|____|	|__| |__| |__| |__|	|__|__|	
c.	|__|__|__|__|.|__|__|__|	|____|	|__| |__| |__| |__|	|__|__|	

Hospital Name: ______________________________

Location: ______________________________

[1]*Refer to Diagnosis Code List;* [2]*Refer to Diagnosis Method Code List;* [3]*Refer to Treatment Code List;*
[4]*Refer to Treatment Outcome Code List*

Form Completed by: FORMBY SIGNED. ____________ Date: |__|__|-|__|__|__|-|__|__|__|__| FORMDT

QC/QA: ____________ Data Entry: 1st ____________ 2nd ____________

WOMEN'S HEALTH

Subject ID: |__|__|__|-|__|__|__|__|__| SUBJID **Visit Date:** |__|__|-|__|__|__|-|__|__|__|__| VISITDT **Visit:** |_____| VISIT

1. Date of last menstrual period: |__|__|-|__|__|__|-|__|__|__|__| LASTMDT

2. Gravida: |_____| GRAVIDA Para: |_____| PARA

3. Are you sexually active? SEX_ACT YESNO.

◯ 1. Yes* **◯ 0. No *(if No, end form)***

4. Are you currently pregnant? CURRPREG YESNO.

◯ 1. Yes * ◯ 0. No

***If Yes, record pregnancy test result on the Blood Chemistry CRF and end form**

5. Do you use a method of family planning? FAMPLAN YESNO.

◯ 1. Yes* ◯ 0. No**

*** If Yes, *(Mark all that apply)***

- ☐ a. Abstinence FPLAN_YA
- ☐ b. Lactation Amenorrhea FPLAN_YB
- ☐ c. Condoms (Male or Female) FPLAN_YC
- ☐ d. Birth control pills FPLAN_YD
- ☐ e. Birth control injections FPLAN_YE
- ☐ f. Birth control implant FPLAN_YF
- ☐ g. Intrauterine device FPLAN_YG
- ☐ h. Natural family planning FPLAN_YH
- ☐ i. Female surgery (tubal ligation) FPLAN_YI
- ☐ j. Male surgery (vasectomy) FPLAN_YJ
- ☐ z. Other, *Specify:* FPLAN_YZ OTHY_TXT __________

**** If No, *(Mark all that apply)***

- ☐ a. Trying to conceive FPLAN_NA
- ☐ b. No partner currently FPLAN_NB
- ☐ c. Partner uses condoms FPLAN_NC
- ☐ d. Can't afford FPLAN_ND
- ☐ e. Desired method not available FPLAN_NE
- ☐ f. Side effects FPLAN_NF
- ☐ g. Interactions with ART FPLAN_NG
- ☐ z. Other, *Specify:* FPLAN_NZ OTHN_TXT __________

Form Completed by: **FORMBY** SIGNED. ________________ Date: |__|__|-|__|__|__|-|__|__|__|__| FORMDT

QC/QA: __________ **Data Entry: 1st** __________ **2nd** __________

PAST/CURRENT OBSTETRIC HISTORY (page 1 of 2) Pregnancy /Seq. #: SEQUENCE

Subject ID: SUBJID Visit Date: VISITDT Visit: VISIT

1. Pregnancy Outcome: *(Note: Subject is excluded from study if pregnant at Visit 1)* PREG_OUT

○ 1. Live birth ○ 2. Abortion ○ 3. Stillbirth ○ 4. Ongoing PREG_OUT.

If Result is 2. Abortion or 3. Stillbirth for Q1: only respond to Q2.- Q8. for corresponding pregnancy.

2. Age at delivery/end of pregnancy: |____| years AGE_DEL

3. Was Mother diagnosed with HIV during this pregnancy? ○ 1. Yes* ○ 0. No ○ 5. NA MOTH_HIV YNONANK.

3a. ***If Yes**, when? ○ 1. 1st Trimester (weeks 1-12) ○ 3. 3rd Trimester (week 27+) ○ 2. 2nd Trimester (weeks 13-27) ○ 4. At Delivery HIV_WHEN HIV_WHEN.

4. PMTCT services accessed? ○ 1. Yes* ○ 0. No PMTCT YNONANK.

4a. ***If Yes**, when? ***(Mark all that apply)***

☐ a. Before delivery PMTCT_A ☐ b. At time of delivery PMTCT_B ☐ c. Postpartum PMTCT_C

5. Was ART eligibility evaluation done? ○ 1. Yes* ○ 0. No ○ 7. Unknown ELIG_EVA YNONANK.

5a. ***If Yes**, method: ***(Mark all that apply)***

☐ a. Clinical (WHO) METH_A ☐ b. Immunological (CD4) ☐ c. Unknown METH_C METH_TXT

6. ARV prescribed to mother? ***(Mark all that apply)*** ○ 0. None ○ **5.** NA ARV_PRES NONENA.

☐ a. Antepartum AZT PRES_A
☐ b. Antepartum Triple ARV prophylaxis PRES_B
☐ c. Antepartum HAART PRES_C
☐ d. Intrapartum SD NVP PRES_D
☐ e. Intrapartum AZT PRES_E
☐ f. Intrapartum 3TC PRES_F
☐ g. Postpartum AZT PRES_G
☐ h. Postpartum 3TC PRES_H
☐ i. Postpartum triple ARV PRES_I
☐ j. Postpartum HAART PRES_J
☐ k. Unknown PRES_K

PRES_TXT

Dates of ARTs should be included on Medication Record CRF

7. Was ARV taken by mother as prescribed? TAKE_PRE NSY.

○ 0. No, never taken ○ 1. Sometimes taken ○ 2. Yes, always taken ○ NA

8. Gestational age at delivery/end of pregnancy: |____| GEST_AGE

○ 1. Weeks ○ 2. Months ○ 5. NA ○ 7. Unknown GEST_P WMNAUNK.

9. Delivered by skilled birth attendant? ○ 1. Yes ○ 0. No D_SKILL YESNO.

10. Place of delivery: ○ 1. Home ○ 2. Hospital D_PLACE HOMEHOSP.

11. Method of delivery: ○ 1. Vaginal ○ 2. C Section DELIVMET DELIVMET.

12. Was mother offered ART postpartum? ○ 1. Yes* ○ 0. No ART_POST YNONANK

12a. ***If Yes**, Duration of post-partum ART? POST_DUR ○ 1. Days ○ 2. Weeks ○ 3. Months POST_P ODWM.

12b. ***If Yes**, Was post-partum ART taken as prescribed? POST_PRE NSY.

○ 0. No, never taken ○ 1. Sometimes taken ○ 2. Yes, always taken

13. ARV prescribed to child? ***(Mark all that apply)*** ○ 0. None ○ 5. NA CHD_ARV NONENA.

☐ a. SD NVP CHD_A
☐ b. Continuous NVP CHD_B
☐ c. 3TC CHD_C
☐ d. AZT CHD_D
☐ e. Triple ARV CHD_E
☐ f. Unknown CHD_F
☐ z. Other, *Specify*: CHD_Z OTH13TXT

QC/QA: ______ Data Entry: 1st ______ 2nd ______

PAST/CURRENT OBSTETRIC HISTORY (page 2 of 2)

Pregnancy /Seq. #: |_____| SEQUENCE

Subject ID: |__|__|__|-|__|__|__|__| SUBJID Visit Date: |__|__|-|__|__|__|-|__|__|__|__| VISITDT Visit: |_____| VISIT

14. Duration ARV prescribed to child postpartum: |_____| CHD_DUR
   ◯ 0. Ongoing ◯ 1. Days ◯ 2. Weeks ◯ 3. Months CHD_ONGO ODWM.

15. Was ARV given to child as prescribed? CHD_GIVE NSY.
   ◯ 0. No, never given ◯ 1. Sometimes given ◯ 2. Yes, always given

16. Was mother counseled on infant/young child feeding? ◯ 1. Yes* ◯ 0. No FEED YESNO.

16a. ***If Yes**, which method was used? FEED_MET FEED_MET.
   ◯ 1. Exclusive breastfeeding ◯ 2. Exclusive replacement feeding ◯ 3. Mixed

17. Duration of breastfeeding? FEED_DUR |_____| ◯ 1. Days ◯ 2. Weeks ◯ 3. Months ◯ 5. NA FEEDFREQ DWMNA.

18. Current health status of child? CHD_STAT CHD_STAT.
   ◯ 1. Alive and well ◯ 3. Deceased Date: |__|__|-|__|__|__|-|__|__|__|__| DEATHDT
   ◯ 2. Alive and chronically ill Age: |_____| CHD_AGE ◯ 1. Days ◯ 2. Months ◯ 3. Years CHD_DMY DMY.

19. Result of Child's HIV tests:

	Test #1	Test #2	Test #3	Test #4
Draw Date	T1_DT |__|__|-|__|__|__|-|__|__|__|__|	T2_DT |__|__|-|__|__|__|-|__|__|__|__|	T3_DT |__|__|-|__|__|__|-|__|__|__|__|	T4_DT |__|__|-|__|__|__|-|__|__|__|__|
Age	T1_AGE |_____| T1_DWM DWM. ◯ 1. Days ◯ 2. Weeks ◯ 3. Months	T2_AGE |_____| T2_DWM DWM. ◯ 1. Days ◯ 2. Weeks ◯ 3. Months	T3_AGE |_____| T3_DWM DWM. ◯ 1. Days ◯ 2. Weeks ◯ 3. Months	T4_AGE |_____| T4_DWM DWM. ◯ 1. Days ◯ 2. Weeks ◯ 3. Months
DNA PCR	|__| Results* T1_DNA NPINDNU.	|__| Results* T2_DNA NPINDNU.	|__| Results* T3_DNA NPINDNU.	|__| Results* T4_DNA NPINDNU.
RDT	|__| Results* T1_RDT NPINDNU.	|__| Results* T2_RDT NPINDNU.	|__| Results* T3_RDT NPINDNU.	|__| Results* T4_RDT NPINDNU.
EIA/WB	|__| Results* T1_EIA NPINDNU.	|__| Results* T2_EIA NPINDNU.	|__| Results* T3_EIA NPINDNU.	|__| Results* T4_EIA NPINDNU.
Viral Load (RNA PCR)	T1_VLLGE LESEQGR. T1_VL |_____|	T2_VLLGE LESEQGR. T2_VL |_____|	T3_VLLGE LESEQGR. T3_VL |_____|	T4_VLLGE LESEQGR. T4_VL |_____|
Unknown	|__| Results* T1_NK NPINDNU.	|__| Results* T2_NK NPINDNU.	|__| Results* T3_NK NPINDNU.	|__| Results* T4_NK NPINDNU.

Results* 0. Negative 1. Positive 2. Indeterminate 5. NA 7. Unknown

20. Definitive HIV status of child: ◯ 0. Negative ◯ 1. Positive* ◯ 7. Unknown DEF_HIV NEGPOSNU
   Added 5. NA to database

20a. ***If Positive**, is child on ART? ◯ 1. Yes* ◯ 0. No** YNONANK.
   CHD_ART

20a1. ***If Yes**, duration: |_____| ART_DUR
   ART_WMY WMY.
   ◯ 1. Weeks
   ◯ 2. Months
   ◯ 3. Years

20a2. ****If No**, (***Mark All that Apply***):
   ☐ a. Non-Adherence ART_A
   ☐ b. Prior ART Toxicity ART_B
   ☐ z. Other, *Specify*: ART_Z
   OTH20TXT ____________________

Form Completed by: FORMBY SIGNED. ____________________ Date: |__|__|-|__|__|__|-|__|__|__|__| FORMDT

QC/QA: __________ Data Entry: 1st __________ 2nd __________

CERVICAL CANCER SCREENING

Page/Seq. #: |____| SEQUENCE

Subject ID: |__|__|__|-|__|__|__|__| SUBJID **Visit Date:** |__|__|-|__|__|__|-|__|__|__|__| VISITDT **Visit:** |____| VISIT

Date of Exam: |__|__|-|__|__|__|-|__|__|__|__| EXAMDT

1. Abnormal vaginal discharge: AB_VAGIN ○ 1. Yes ○ 0. No YESNO.
2. Heavy menstrual bleeding (more than one pad/hour): HM_B ○ 1. Yes ○ 0. No YESNO.
3. Intermenstrual bleeding: INTER_B ○ 1. Yes ○ 0. No YESNO.
4. Post-coital bleeding: POST_B ○ 1. Yes ○ 0. No YESNO.
5. Pain during sexual intercourse: PAIN ○ 1. Yes ○ 0. No YESNO.
6. Lower abdominal pain not related to menstruation: LOWPAIN ○ 1. Yes ○ 0. No YESNO.
7. External Genitalia: EXTERG ○ 0. Normal ○ 1. Abnormal NORMAB.
8. Vagina Wall: VAGINA ○ 0. Normal ○ 1. Abnormal NORMAB.
9. Bimanual exam: ○ 0. Normal ○ 1. Abnormal* ○ 6. Not Done B_EXAM NORMABND.

9a. ***If Abnormal, (Mark all that apply)***
- ☐ a. Adnexal mass B_EXAMA
- ☐ b. Enlarged uterus B_EXAMB
- ☐ z. Other abnormality, *Specify:* B_EXAMZ OTH_9TXT

10. Cervical Exam (VIA/VILI): ○ 0. Normal ○ 1. Abnormal* ○ 6. Not Done C_EXAM NORMABND.

10a. ***If Abnormal, (Mark all that apply)***
- ☐ a. Acetowhite/yellow lesion C_EXAMA
- ☐ z. Other abnormality, *Specify:* C_EXAMZ OTH10TXT
- ☐ b. Cryotherapy performed C_EXAMB

11. Was the subject referred? ○ 1. Yes* ○ 0. No REF YESNO.

11a. ***If Yes**, why?:
- ☐ a. Acetowhite/yellow lesion not amenable to cryotherapy
- ☐ b. Cryotherapy not available REF_WHYA - REFWHYC
- ☐ c. Findings suspicious of cancer
- ☐ z. Other, *Specify:* REF_WHYZ OT11ATXT

11b. ***If Yes**, outcome of referral: ○ Did not see consultant REF_NOCON
- ☐ a. LEEP REF_OUTA – REF_OUTF
- ☐ b. Hysterectomy
- ☐ z. Other, *Specify:* REF_OUTZ OT11BTXT
- ☐ c. Observation
- ☐ d. Biopsy
- ☐ e. Radiotherapy
- ☐ f. Cryotherapy

Form Completed by: FORMBY SIGNED. __________ Date: |__|__|-|__|__|__|-|__|__|__|__| FORMDT

QC/QA: ________ Data Entry: 1st ________ 2nd ________

MISSED VISIT

Subject ID: |__|__|__|-|__|__|__|__| SUBJID **Visit Date:** |__|__|-|__|__|__|-|__|__|__|__| VISITDT **Visit:** |______| VISIT

Form to be completed when the allotted visit window has closed.
Use the scheduled visit number for this form.

Visit Window:

Follow the schedule of 180-day study visit windows based on the subject's enrollment date. Within the visit windows, the range to complete the study visit is 90 days before the scheduled visit date, or within 90 days after. A Missed Visit form must be completed after the visit window has closed. A visit must never be *completed* less than 90 days since the previous completed study visit. A visit date must be *scheduled* no less than 90 days, and no more than 180 days, after the previous completed visit.

1. Last Completed Study Visit #: |______| LAST_VIS

2. Date of Last Completed Study Visit :

|__|__|-|__|__|__|-|__|__|__|__| LAST_DT

3. Date of most recent subject contact (any contact):

|__|__|-|__|__|__|-|__|__|__|__| CONTACDT

4. Reason for missed visit: ***(Mark all that apply)***

- ☐ a. Unknown/Unable to contact REAS_A
- ☐ b. Work schedule/conflict REAS_B
- ☐ c. Hospitalized or ill REAS_C
- ☐ d. Moved out of area REAS_D
- ☐ e. Deceased, Date of Death: REAS_E |__|__|-|__|__|__|-|__|__|__|__| DEATHDT
- ☐ f. No longer wishes to participate in study REAS_F
- ☐ z. Other, *Specify*: REAS_Z ______ OTH_ZTXT ______

Form Completed by: **FORMBY** SIGNED. ______________ Date: |__|__|-|__|__|__|-|__|__|__|__| FORMDT

QC/QA: ________ **Data Entry: 1st** ________ **2nd** ________

STATUS CHANGE

Page/Seq. #: |__| SEQUENCE

Subject ID: |__|__|__|-|__|__|__|__|__| SUBJID Visit Date: |__|__|-|__|__|__|-|__|__|__|__| VISITDT Visit: |__| VISIT

Date of Status Change: |__|__|-|__|__|__|-|__|__|__|__| SCDT

Date of Last Protocol Visit:
(including current visit) |__|__|-|__|__|__|-|__|__|__|__| LASTDT

Is the subject being terminated from the study? ◯ 1. Yes ◯ 0. No BE_TERM YESNO.

Status Change: SCHANGE SCHANGE.

◯ 1*. Transfer to Site – Enter Site Code[1]: |__|__|__| SC_TO SITECODE.
Transfer from Site – Enter Site Code[1]: |__|__|__| SC_FROM SITECODE.
◯ 2*. Reactivated
*If Status Change = 1, 2, or 3 Date of re-consent: |__|__|-|__|__|__|-|__|__|__|__| RECONDT
◯ 3*. New HIV Infection → *(If enrolled as HIV Negative, re-enroll subject as HIV Positive)*

◯ 4. Subject withdrew consent
◯ 5. Lost to Follow-up (no contact attained for 360 days since initial missed visit)
◯ 6. Moved out of area- no research contact available
◯ 7. Became incarcerated
◯ 8. Protocol violation, *Specify*: SC8_TXT ____________________
◯ 9. Concurrent Illness precludes participation
◯ 90. Other, *Specify*: OTH_TXT ____________________

◯ 10. Death, complete additional information below:

Date of Death: |__|__|-|__|__|__|-|__|__|__|__| DEATHDT

10a. Was an autopsy performed? ◯ 1. Yes ◯ 0. No AUTOPSY YESNO.

10b. Primary cause of death? ◯ 7. Unknown DTH_PRI DTH_PRI.
◯ 1. Known, code[2]: |__|__|__|__|.|__|__|__| PRI_CODE *
◯ 2. Known, Other, *Specify*: PRI_TXT ____________________

10c. Secondary if any. DTH_SEC DTH_SEC.
◯ 1. Known, code[2]: |__|__|__|__|.|__|__|__| SEC_CODE *
◯ 2. Known, Other, *Specify*: SEC_TXT ____________________

[1]*Refer to Site Code List:* [2]*Refer to Diagnosis Code List*

All information entered onto this Case Report Form by myself or my designee for this subject is correct to the best of my knowledge.

PI Signature: PI_SIGN ____________________ Date: |__|__|-|__|__|__|-|__|__|__|__| PIDT

Form Completed by: FORMBY SIGNED. ____________________ Date: |__|__|-|__|__|__|-|__|__|__|__| FORMDT

QC/QA: QC_QA SIGNED. ________ Data Entry: 1st ________ 2nd ________

PATHOLOGY

Page/Seq. #: |____| SEQUENCE

Subject ID: |__|__|__|-|__|__|__|__| SUBJID **Visit Date:** |__|__|-|__|__|__|-|__|__|__|__| VISITDT **Visit:** |____| VISIT

Other Pathology Results (Male and Female)

Date: |__|__|-|__|__|__|-|__|__|__|__| PATHDT

Specimen Code *(refer to Specimen Code List)*: |__|__|__| PATHSPCM SPECIMEN. *or, if no code,* Other, *Specify:* ______________ PATHSTXT

Diagnosis Code *(refer to Diagnosis Code List)*: |__|__|__|.|__|__| PATHDX* *or, if no code,* Other, *Specify:* ______________ PATHDTXT

Results (path report summary): PATHRTXT

____

____

____

PAP Smear Results (Female only)

Date: |__|__|-|__|__|__|-|__|__|__|__| PAPDT

1. Specimen satisfactory for evaluation? PAPSAT YESNO. ◯ 1. Yes* ◯ 0. No ⟶ If No, **End Form.**

1a. *If Yes, Negative for intraepithelial lesion or malignancy? PAPNEG YESNO. ◯ 1. Yes ◯ 0. No**

1a1. ***Mark all that apply***:

- ☐ a. *Trichomonas vaginalis* PAP_A
- ☐ b. Fungal organisms consistent with *Candida* PAP_B
- ☐ c. Shift in flora suggestive of bacterial vaginosis PAP_C
- ◯ None PAPNONE
- ☐ d. Cellular changes consistent with herpes simplex virus PAP_D
- ☐ e. Other non-neoplastic changes, *Specify:* PAP_E ______________ PAP_ETXT

1a2. ****If No** (***Mark a. Squamous Cell, b. Glandular Cell, and/or c. Other abnormalities as applicable***):

a. Squamous Cell: SQUAMCEL SQUAMCEL.

- ◯ 1. Atypical squamous cells of undetermined significance (ASCUS)
- ◯ 2. Atypical squamous cells cannot exclude HSIL (ASCH)
- ◯ 3. Low grade squamous intraepithelial lesion (LSIL) – encompassing HPV/mild dysplasia/ cervical intraepithelial neoplasia (CIN 1)
- ◯ 4. High grade squamous intraepithelial lesion (HSIL) – encompassing moderate and severe dysplasia, carcinoma in situ (CIN 2 and CIN 3)
- ◯ 5. Squamous cell carcinoma
- ◯ 0. No squamous cell abnormality

b. Glandular Cell: GLANDCEL GLANDCEL.

- ◯ 1. Atypical glandular cells (AGC)
- ◯ 2. Atypical glandular cells, favor neoplastic
- ◯ 3. Endocervical adenocarcinoma in situ (AIS)
- ◯ 4. Adenocarcinoma
- ◯ 0. No glandular cell abnormality

c. Other abnormality ***(Mark all that apply)***:

- ☐ a. Endometrial cells in a woman ≥40 years of age OTHABN_A
- ☐ z. Other, *Specify:* ______________ OTHABN_Z OTHABTXT
- ◯ No other abnormality NOOTHAB

Form Completed by: FORMBY SIGNED. ______________ Date: |__|__|-|__|__|__|-|__|__|__|__| FORMDT

QC/QA: ____________ Data Entry: 1st ____________ 2nd ____________

RV329 - AFRICOS CRFs

ANNOTATION KEY

SAS VARIABLE/SAS DATA SET LABELS IN BLUE
IE – SUBJID/R_SYMPT

CLINPLUS FORMAT LABELS/CLINPLUS SCREEN LABELS IN RED
IE – YESNO/R_SYMPT2. IF HIGHLIGHTED IN PURPLE, REFERS TO STUDY CODE LIST WHICH HAS BEEN INTEGRATED INTO CLINPLUS AS A FORMAT.
CHECKBOX VARIABLES, IE, IND_A, HAVE THE DEFAULT CHECKBOX. FORMAT AND THEREFORE ARE NOT INDIVIDUALLY ANNOTATED.
FORMS ANNOTATED AS "SEQUENCED" IN RED INDICATE ONE FORM ACCOMMODATES MULTIPLE RECORDS IN CLINPLUS, IE, WHO CLASS.

CLINPLUS/SAS VARIABLES ASSOCIATED STUDY CODES WHICH ARE NOT CLINPLUS-FORMATTED ARE NOTED WITH *

REFER TO CLINPLUS DATA STRUCTURE DOCUMENTATION FOR DETAILS ON CLINPLUS/SAS VARIABLES

REFER TO THE FORMAT SAS SCRIPT FOR DETAILS ON FORMATS

REFER TO THE STUDY CODES DOCUMENT FOR DETAILS ON STUDY CODE LISTS

RV329 - AFRICOS CRFs

Version History

Changes made in version 1.7
Q2 Creatinine – added CRET_LGE (< = >) and format LESEQGR.
Q12 Total Bilirubin – added format LESEQGR.

Changes made in version 1.6
CRF OBX_HX1
Q7 – Added option NA to format NSY in field TAKE_PRE (9/23/2013)

Changes made in version 1.5 (30OCT2013)
CRF OBX_HX1
Q5a – Added METH_TXT comment field for ART eligibility evaluation method comment
Q6 – Added PRES_TXT free text field for prescribed ARV comment

Changes made in version 1.4 (13SEP2013)

OBS_HX1
Q12a changed format from DWM to ODWM for added option ONGOING

Changes made in version 1.3 (12Aug2013)

Extract
Added following variable to Q12 VLDT_E-J/VLCD4_E-J/VLCD4P_E-J/
VL_LGE_E-J/VLCOPY_E-J/RESDN_E-J

Blood Chemistry
Added-Labcd2-7,Labcd8-13,Labcd14-17,Tbil_lge,Labcd20-27

Serology
Added-LABCD1-18

HIV Status
Q2e – added variable ELIG_D for option d. Plan B+

Changes made in version 1.2 (18Jul2013)

OBS_HX2
Q20 – changed format from NEGPOSNK to NEGPOSNU for added option NA

RV329 - AFRICOS CRFs

Version History

Changes made in version 1.1 (01Jul2013)

OBS_HX1:
Q3 – changed format from YESNONA to YNONANK for added option UNKOWN
Q4 – changed format from YESNONA to YNONANK for added options NA, UNKNOWN
Q5 – changed format from YESNONK to YNONANK for added options NA
Q6 – added option UNKNOWN to format NONENA
Q12 – changed format from YESNO to YNONANK for added options NA, UNKNOWN,
Q13 - added UNKNOWN to format CHD_ARV

OBS_HX2:
Q16 – changed format from YESNO to YNONANK for added options NA, UNKNOWN
Q17 - added option ONGOING to format DWMNA
Q18 - added option UNKNOWN to format CHD_STAT
Q20a – changed format from YESNO to YNONANK for added option UNKNOWN,

EXTRACT:
Q12a, b, c, d – (VLCOPY_A, VLCOPY_B, VLCOPY_C, VLCOPY_D) added format FIELD_DA for none numerical responses.
