# Supplementary material for: HIV virologic failure and its predictors among HIV-infected adults on antiretroviral therapy in the African Cohort Study
Source: PLoS One. 2019 Feb 5;14(2):e0211344. doi: 10.1371/journal.pone.0211344 (PMC6363169; doi:10.1371/journal.pone.0211344)
Supplement: S2 PDF — (PDF) [file pone.0211344.s002.pdf]

Annotated

Case Report Form

For

SUBJECT QUESTIONNAIRE

(Enrollment/Visit 1 and Subsequent Visits)

Protocol RV329 / WRAIR 1897

Version 1.2

July 8, 2013

African Cohort Study (AFRICOS)

Study Conducted by US Military HIV Research Program

Study Supported by

Data Coordinating and Analysis Center (DCAC), MHRP

Henry M.Jackson Foundation (HJF)

RV329 AFRICOS

SUBJECT QUESTIONNAIRE*

(Enrollment/Visit 1 and Subsequent Visits)

TABLE OF CONTENTS

QUESTIONNAIRE	PAGE	VISIT
1. **Demographics**	3	All
2. **Section A** – HIV	6	All
3. **Section B**<br>Sexual History and Behaviors	9	All
4. **Section C**<br>Social History and Behaviors	13	All
5. **Section D**<br>General Health and Medical Conditions	16	All
6. **Section E** - Cognition	19	All

Subject Questionnaire can be used as a Source Document

SUBJECT QUESTIONNAIRE

Subject ID: |__|__|__|-|__|__|__|__|__| SUBJID **Visit Date:** |__|__|-|__|__|__|-|__|__|__|__| VISITDT **Visit #:** |__VISIT|

"Thank you for participating in our health study. I would like to ask you various questions about you and your medical history. Please answer the questions truthfully and as accurately as you can. If you do not want to answer a question, you can refuse to answer and we will skip to the next one. You have this right not to answer a question, and it is better not to answer a question than to provide false information. If you do not know the answer to a question, please indicate so. However, we encourage you to answer all the questions to the best of your knowledge. This survey does not have your name or identification information on it, only your confidential study ID. Your choice to complete this survey will not disrupt or prevent your health care. All the information you provide will be kept confidential."

DEMOGRAPHICS

1. What is your current marital status? MARITAL MARITALZ.
   - ◯ 1. Single
   - ◯ 2. Married (traditional or civil, includes living together with partner)*
   - ◯ 3. Divorced/Separated
   - ◯ 4. Widowed
   - ◯ 90. Other, specify: ______MARITXT______

   1a. ***If Married,** how many spouses do you have now?

   |______| **# of spouses** MARINUM

2. Are you able to read and write? READWRIT YESNO.
   - ◯ 1. Yes
   - ◯ 0. No

3. Highest level of education completed? EDUCAT EDUCATZ.
   - ◯ 0. No schooling
   - ◯ 1. Some primary school
   - ◯ 2. Completed primary school
   - ◯ 3. Some secondary school
   - ◯ 4. Completed secondary school
   - ◯ 5. Some post-secondary school (university)
   - ◯ 6. Completed post-secondary school
   - ◯ 7. Some vocational or training school
   - ◯ 8. Completed vocational or training school
   - ◯ 90. Other, specify: ______EDUTXT______

4. Are you currently employed? EMPLOYED YESNO.
   - ◯ 1. Yes*
   - ◯ 0. No

   ***If Yes,**

   4a. Which occupation earns the majority of your income?

   Specify Primary Occupation: PROCCTXT ____________________

QC/QA: ____________ **Data Entry: 1st** ____________ **2nd** ____________

3

SUBJECT QUESTIONNAIRE

Subject ID: |__|__|__|-|__|__|__|__|__| SUBJID **Visit Date: |__|__|-|__|__|__|-|__|__|__|__|** VISITDT **Visit #: |____|** VISIT

4b. Please classify your Primary Occupation:
(Mark all that apply)

OCCUP_A – OCCUP_I

- ☐ a. Student
- ☐ b. Housewife
- ☐ c. Personal or domestic services
- ☐ d. Unskilled laborer
- ☐ e. Farmer
- ☐ f. Entertainment/Services/Hospitality
- ☐ g. Skilled trade (tailor, hairdresser, etc.)
- ☐ h. Professional/managerial
- ☐ i. Commerce/business
- ☐ z. Other, specify: ____________________ OCCUPTXT

OCCUP_Z

5. How many people currently live in your household (including yourself)?

ADULTNUM **|______| Adults (≥ 18 years)** CHILDNUM **|______| Children**

6. Who currently lives in your household?
(Mark all that apply) HHOLD_A – HHOLD_I

- ☐ a. Spouse/partner
- ☐ b. Parent (yours or spouse's/partner's)
- ☐ c. Brother or sister
- ☐ d. Sons or daughters
- ☐ e. Grandparent (yours or spouse's/partner's)
- ☐ f. Extended adult family member (cousin, uncle/aunt, etc.)
- ☐ g. Extended adolescent family member (niece, nephew, etc.)
- ☐ h. Friend
- ☐ i. Roommate
- ☐ z. Other, specify: HHOLD_Z ____________________ HHOLDTXT
- ○ Live alone/Only you HH_ALONE

7. How many people do you provide for financially (including yourself)? PROVIDE

PROVIDE.

- ○ 0. No person
- ○ 1. 1 person
- ○ 2. 2-5 persons
- ○ 3. 6-10 persons
- ○ 4. > 10 persons

QC/QA: ____________ **Data Entry: 1st** ____________ **2nd** ____________

SUBJECT QUESTIONNAIRE

Subject ID: |__|__|__|-|__|__|__|__|__| SUBJID **Visit Date:** |__|__|-|__|__|__|-|__|__|__|__| VISITDT **Visit #:** |__| VISIT

8. What is your household's total income per week?

HHINCOME

|________________|
(amount)

HCURRTYP CURRTYPZ.

- ◯ 1. Kenyan Shillings
- ◯ 2. Nigerian Naira
- ◯ 3. Tanzanian Shillings
- ◯ 4. Ugandan Shillings
- ◯ 90. Other, specify: ____________________ HCURRTXT

9. How far do you live from this facility? ***(For subsequent visits, only respond if you have moved since the last visit)***

HOWFRKM HOWFREST

|________| km ☐ Estimated

10. How long does it take to get to this health facility from your home?

HOWLONGH HOWLONGM HOWLEST

|________| Hours |________| Minutes ☐ Estimated

11. How much did it cost to travel to this health facility from home?

HOMECOST

|________________|
(amount)

TCURRTYP CURRTYPZ.

- ◯ 1. Kenyan Shillings
- ◯ 2. Nigerian Naira
- ◯ 3. Tanzanian Shillings
- ◯ 4. Ugandan Shillings
- ◯ 90. Other, specify: ________________________ TCURRTXT

12. Have you had enough food to eat over the past 12 months?

- ◯ 1. Yes
- ◯ 0. No

FOOD YESNO.

13. On average, how many meals do you have in a day?

|________| **# of meals per day** MEALNUM

END OF DEMOGRAPHICS

QC/QA: ____________ **Data Entry: 1st** ____________ **2nd** ____________

SUBJECT QUESTIONNAIRE

Subject ID: |__|__|__|-|__|__|__|__|__| SUBJID **Visit Date:** |__|__|-|__|__|__|-|__|__|__|__| VISITDT **Visit #:** |____| VISIT

"I would like, now, to ask you some questions relating to HIV. Again, please answer them to the best of your knowledge, and know that you may refuse any question. All your answers will be kept confidential. Thank you."

SECTION A - HIV

1. What is your HIV status? HIVSTAT POSNEG.
   - ◯ 1. Positive
   - ◯ 0. Negative

2. How do you think you were infected with HIV?
   (Answer only if HIV + at Visit 1, or if HIV status changed from HIV – to HIV + since the last visit)
   HIVINFCT HIVINFCT.
   - ◯ 1. Sexual contact with regular partner
   - ◯ 2. Sexual contact with casual partner
   - ◯ 3. Forced sexual contact
   - ◯ 4. Blood transfusion
   - ◯ 5. Open wound-to-wound contact (sports, military exercises)
   - ◯ 6. Combat activities
   - ◯ 7. Intravenous drug use (sharing a needle)
   - ◯ 90. Other, specify: ______ HIVINTXT ______

3. What is your spouse's/partner's HIV status? PHIVSTAT POSNEGNA.
   - ◯ 1. Positive*
   - ◯ 0. Negative
   - ◯ 5. N/A
   - ◯ 7. Unknown

   3a. ***If Positive,** is your spouse/partner on antiretroviral therapy? ______ Q3_comments ______
   ◯ 1. Yes ◯ 0. No PHIVARTX YESNO.

4. For **Visit 1**, indicate which members of your immediate family have *ever* been diagnosed as infected with HIV.

   For each **Subsequent Visit**, indicate which members of your immediate family have been diagnosed as infected with HIV *since your last scheduled study visit*.

   (Mark all that apply) FHIV_A – FHIV_D
   - ☐ a. Son or daughter
   - ☐ b. Brother or sister
   - ☐ c. Mother
   - ☐ d. Father
   - ◯ None FHIVNONE ______ Q4_comments ______

5. **If HIV Negative,** how often have you accessed voluntary HIV counseling and testing services? ***(For Subsequent Visits, answer how often since your last scheduled study visit)***
   CTHIVFRQ MONTHFRQ.
   - ◯ 1. Monthly
   - ◯ 2. Every other month
   - ◯ 3. Every three months
   - ◯ 4. Every six months
   - ◯ 90. Other, specify: ______ CTHIVTXT ______

QC/QA: ______ Data Entry: 1st ______ 2nd ______

SUBJECT QUESTIONNAIRE

Subject ID: |__|__|__|-|__|__|__|__|__| SUBJID **Visit Date:** |__|__|-|__|__|__|-|__|__|__|__| VISITDT **Visit #:** |__VISIT__|

"I would like, now, to ask you some questions relating to HIV. Again, please answer them to the best of your knowledge, and know that you may refuse any question. All your answers will be kept confidential. Thank you."

Questions 6 through 10 below, and 11 through 14 on the next page, apply to HIV Positive subjects only

6. Are you taking Antiretroviral (ARV) drugs? ◯ 1. Yes ◯ 0. No -> *(If No, skip to 10.)* TAKEARV YESNO.

6a. How many pills are in your expected dose? |PILLNUM| **# of pills per dose** ◯ No Response PILLNR

6b. In the past month, how many days did you miss a dose of your ARVs?

◯ 0. None
◯ 1. 1-2 days
◯ 2. 3-5 days
◯ 3. 6-10 days
◯ 4. More than 10 days

MISSARV MISSARV.

7. What are some of the reasons that have caused you to miss a dose of your ARV meds? *(Mark all that apply)*

For Subsequent Visits, provide reasons for missing a dose <u>since your last scheduled study visit</u>

MSARV_A – MSARV_P

☐ a. Meds make you feel ill
☐ b. Too many pills to take
☐ c. You simply forgot
☐ d. You already felt too ill
☐ e. You had shared your meds
☐ f. You lost or ran out of meds
☐ g. You were unable to pay for meds
☐ h. You had delivery or travel problems
☐ i. The pharmacy was out of stock
☐ j. Lack of food with meds
☐ k. You drank too much alcohol
☐ l. You felt depressed
☐ m. To avoid stigma for taking meds
☐ n. You believe in using herbs instead
☐ o. You believe in faith healing instead
☐ p. You were feeling better
☐ z. Other, specify: MSARV_Z ______MSARVTXT______

◯ **Mark if None** MSARNONE

8. How much are you normally charged for a clinic visit for your ARVs? ACURRTYP CURRTYPZ.

ARVCOST |________________|
(amount)

◯ 1. Kenyan Shillings
◯ 2. Nigerian Naira
◯ 3. Tanzanian Shillings
◯ 4. Ugandan Shillings
◯ 90. Other, specify: ______ACURRTXT______

9. Do you have a treatment supporter or treatment companion, that is, someone who supports you in taking your ARVs? ARVSUPP

◯ 1. Yes ◯ 0. No YESNO.

10. Do you receive food as part of your HIV care? HIVFOOD

◯ 1. Yes* ◯ 0. No YESNO.

10a. ***If Yes,** what kind of food aid do you receive? *(Mark all that apply)*

☐ a. Standard food HVFD_A
☐ b. Nutritional supplement HVFD_B
☐ z. Other, specify: HVFD_Z ______HVFDTXT______

QC/QA: ____________ Data Entry: 1st ____________ 2nd ____________

7

SUBJECT QUESTIONNAIRE

Subject ID: |__|__|__|__|-|__|__|__|__|__| SUBJID **Visit Date:** |__|__|-|__|__|__|-|__|__|__|__| VISITDT **Visit #:** |__ VISIT|

**For Subsequent Visits, Questions 11 through 14 below refer to events that occurred since your last scheduled study visit*

*11. Which of the following types of HIV-associated Stigma have you experienced? *(Mark all that apply)*

HVSTE_A – HVSTE_C

- ☐ a. Social isolation
- ☐ b. Physical violence
- ☐ c. Broken family relationships
- ☐ z. Other, specify: ___HVSTE_Z___ ___HVSTETXT___

○ **Mark if None** HVSTENO

*12. How has HIV-associated Stigma disrupted your HIV care? *(Mark all that apply)*

- ☐ a. Missed visits HVSTD_A
- ☐ b. Missed medicine HVSTD_B

HVSTDNO ○ **Mark if None**

*13. Who has become aware of your HIV status? *(Mark all that apply)*

WHIV_A – WHIV_I

- ☐ a. Spouse/partner
- ☐ b. Parent (yours or spouse's/partner's)
- ☐ c. Brother or sister
- ☐ d. Sons or daughters
- ☐ e. Grandparent (yours or spouse's/partner's)
- ☐ f. Extended adult family member (cousin, uncle/aunt, etc.)
- ☐ g. Extended adolescent family member (niece, nephew, etc.)
- ☐ h. Friend
- ☐ i. Roommate
- ☐ z. Other, specify: ___WHIV_Z___ ___WHIVTXT___

WHIVNONE ○ No one

*14. How often have you attended an HIV support group? SGHIVFRQ SUPPFRQ.

- ○ 0. Not at all
- ○ 1. Less than once a month
- ○ 2. Once a month
- ○ 3. More than once a month
- ○ 4. Several times within a month

15. We'd like to know how satisfied you are with the services you receive at this ART clinic. Please give us your honest opinion as it will help us to improve. I will read a list of services and please tell me if you are **satisfied** with the service, or if it **needs to improve *(applies to all subjects regardless of HIV status)***

Services ARTSRV_A – ARTSRV_F	Satisfied (1)	Needs to improve (2)	Refused (8)
a. Waiting time	SERVSAT.		
b. Health care worker skills			
c. Health care workers attitudes			
d. Quality of the clinic building			
e. Overall quality of the care you receive			
f. Anything else you feel needs improving?<br>Specify: ___ARTSRTXT___			8

QC/QA: __________ Data Entry: 1st __________ 2nd __________ **END OF SECTION A - HIV**

SUBJECT QUESTIONNAIRE

Subject ID: |__|__|__|-|__|__|__|__|__| SUBJID **Visit Date:** |__|__|-|__|__|__|-|__|__|__|__| VISITDT **Visit #:** |__| VISIT

"I would like, now, to ask you some questions relating to sexual history and behaviors. For the purpose of this study, sex refers to any vaginal, oral, or anal intercourse. When we ask you about persons you have had sex with, please respond with all partners: male or female. We understand that this is a sensitive and personal topic. However, it is important for the purpose of this study that you are as open and honest as possible. Again, we want to assure you that anything you say will be kept confidential. Please answer them to the best of your knowledge, and know that you may refuse any question. Thank you."

SECTION B – SEXUAL HISTORY & BEHAVIORS

1. Have you ever had sexual intercourse? (***skip if answered Yes at a previous visit***)

   ◯ 1. Yes HADSEX YESNO.
   ◯ 0. No -> ***(Skip to Section C.)***

2. Approximately how old were you the first time you had sexual intercourse? (***skip if answered Yes at a previous visit***)

   |__HADSEXAG__| **years old** HADSEXNK ◯ Unknown

3. For **Visit 1**, indicate approximately how many sexual partners you have had in your *lifetime*, including any current partners.

   For each **Subsequent Visit**, indicate how many sexual partners you have had *since your last scheduled study visit*, including any current partners.

   SXPTLNUM FIELD_DA.
   |__________| **# of partners**

4. How many times in the last month have you engaged in sexual intercourse? SXACTFRQ NUMFRQ.

   ◯ 0. None ◯ 3. 7-10
   ◯ 1. 1-3 ◯ 4. More than 10
   ◯ 2. 4-6 ◯ 8. No Response

5. ***"Regular partners" include your spouse, boyfriend/girlfriend, or any person with whom you have a committed relationship.***

   In the past 6 months, how many regular partners did you have sexual intercourse with?

   SXPTRNUM
   |__________| **# of regular partners *(If None, write "0" in the box and Skip to 11.)***

Answer the following questions about the last time you had sexual intercourse with your regular partner.

6. Was a condom used the last time you had sexual intercourse with this regular partner?

   ◯ 1. Yes USECONDR
   ◯ 0. No YESNO.

7. How often was a condom used with this regular partner in the last 6 months?

   ◯ 0. Never CONDRFRQ
   ◯ 1. Sometimes FREQZ.
   ◯ 2. Frequently
   ◯ 3. All the time

QC/QA: ______________ Data Entry: 1st ______________ 2nd ______________

SUBJECT QUESTIONNAIRE

Subject ID: |__|__|__|-|__|__|__|__|__| SUBJID **Visit Date:** |__|__|-|__|__|__|-|__|__|__|__| VISITDT **Visit #:** |___| VISIT

8. Was this regular partner's status HIV positive, HIV negative, or unknown?

   ◯ 1. HIV positive (+) RHIVSTAT POSNEGHV.
   ◯ 2. HIV negative (-)
   ◯ 7. Unknown

9. What is the gender of this regular partner? GENDERR GENDERNR.

   ◯ 1. Male
   ◯ 2. Female
   ◯ 8. No Response

10. What type of sexual intercourse did you have? ***(Mark all that apply)***

   ☐ a. Vaginal SXTYPR_A - SEXTYPR_D
   ☐ b. Anal insertive
   ☐ c. Anal receptive
   ☐ d. Oral

   ◯ No Response SXTYPRNR

11. ***"Casual partners" includes any person with whom you have sexual intercourse but did not have a committed relationship. This <u>does not</u> include your spouse, boyfriend/girlfriend, or regular partners.***

   In the past 6 months, how many <u>casual partners</u> did you have sexual intercourse with?
   SXPTCNUM

   |__________| **# of casual partners** ***(If None, write "0" in the box and Skip to 17.)***

Answer the following questions about the <u>last time</u> you had sexual intercourse with your <u>casual partner</u>.

12. Was a condom used the last time you had sexual intercourse with this casual partner?

   ◯ 1. Yes USECONDC
   ◯ 0. No YESNO.

13. How often was a condom used with this casual partner in the last 6 months?

   ◯ 0. Never CONDCFRQ
   ◯ 1. Sometimes FREQZ.
   ◯ 2. Frequently
   ◯ 3. All the time

14. Was this casual partner's status HIV positive, HIV negative, or unknown?

   ◯ 1. HIV positive (+) CHIVSTAT POSNEGHV.
   ◯ 2. HIV negative (-)
   ◯ 7. Unknown

QC/QA: ______________ Data Entry: 1st ______________ 2nd ______________

SUBJECT QUESTIONNAIRE

Subject ID: |__|__|__|-|__|__|__|__|__| SUBJID **Visit Date: |__|__|-|__|__|__|-|__|__|__|__|** VISITDT **Visit #: |____|** VISIT

15. What is the gender of this casual partner? GENDERC GENDERNR.
   - ◯ 1. Male
   - ◯ 2. Female
   - ◯ 8. No Response

16. What type of sexual intercourse did you have? ***(Mark all that apply)*** SXTYPC_A - SXTYPC_D
   - ☐ a. Vaginal
   - ☐ b. Anal insertive
   - ☐ c. Anal receptive
   - ☐ d. Oral
   - ◯ No Response SXTYPCNR

We now would like to collect some more information about any sexual partners you have had.

(*For Subsequent Visits, Questions 17 through 19 below refer to events that occurred since your last scheduled study visit)

*17. Have you <u>received</u> money, shelter, food, drugs, favors, or gifts in exchange for sex? SEXRCV YESNONR.
   - ◯ 1. Yes*
   - ◯ 0. No
   - ◯ 8. No Response

*18. Have you received sex after exchanging any of the following? ***(Mark all that apply)*** SEXRCV_A - SEXRCV_E
   - ☐ a. Providing shelter
   - ☐ b. Food
   - ☐ c. Drugs
   - ☐ d. Favors
   - ☐ e. Gifts SEXRCV_Z SEXRCTXT
   - ☐ z. Other, specify: ______________________________

SXRCNONE ◯ **Mark if None**

*19. Have you had sex with any of the following? ***(Mark all that apply)*** HADSEX_A – HADSEX_D
   - ☐ a. Bar/pub/karaoke worker
   - ☐ b. Boda-boda or motorbike driver
   - ☐ c. Long distance truck driver
   - ☐ d. Commercial sex worker

HDSXNONE ◯ **Mark if None**

QC/QA: ____________ Data Entry: 1st ____________ 2nd ____________

11

SUBJECT QUESTIONNAIRE

Subject ID: |__|__|__|-|__|__|__|__|__| SUBJID **Visit Date: |__|__|-|__|__|__|-|__|__|__|__|** VISITDT **Visit #: |____|** VISIT

We now would like to collect some information about any condom use and availability.

20. Have you used a condom? ***(For Subsequent Visits, refer to events that occurred since your last scheduled study visit)***

USECOND
YESNONR.

- ◯ 1. Yes
- ◯ 0. No
- ◯ 8. No Response

21. If you do not (typically/generally) use condoms, what are the reasons you don't like to use them?

(Mark all that apply)

NOCOND_A - NOCOND_J

- ◯ a. None. I (typically/generally) use condoms
- ☐ b. Allergic
- ☐ c. Don't trust them
- ☐ d. Don't have the right brand
- ☐ e. I trust my partner(s)
- ☐ f. Too expensive
- ☐ g. Condoms are too small
- ☐ h. God doesn't approve
- ☐ i. Want flesh to flesh sex
- ☐ j. It is embarrassing to buy them
- ☐ z. Other, *specify*: ________ NOCOND_Z NOCONTXT ________

22. How easy or difficult is it for you to get condoms?

GETCOND
GETCOND.

- ◯ 0. Very easy
- ◯ 1. Easy
- ◯ 2. Difficult
- ◯ 3. Very difficult
- ◯ 4. I don't try to get condoms

23. Do you have access to free condoms?

FREECOND
YESNONR.

- ◯ 1. Yes
- ◯ 0. No
- ◯ 8. No Response

END OF SECTION B – SEXUAL HISTORY & BEHAVIORS

QC/QA: ______________ Data Entry: 1st ______________ 2nd ______________

12

SUBJECT QUESTIONNAIRE

Subject ID: |__|__|__|-|__|__|__|__|__| SUBJID **Visit Date: |__|__|-|__|__|__|-|__|__|__|__|** VISITDT **Visit #: |____|** VISIT

"I would like, now, to ask you some questions relating to social behaviors. We understand that this is a sensitive and personal topic. However, it is important for the purpose of this study that you are as open and honest as possible. Again, we want to assure you that anything you say will be kept confidential. Please answer them to the best of your knowledge, and know that you may refuse any question. Thank you."

SECTION C – SOCIAL HISTORY & BEHAVIORS

1. Do you consume alcohol? ALCOHOL YESNO.

- ◯ 1. Yes
- ◯ 0. No -> (***Skip to 2.)***

1a. How many days a week do you drink? ALCOHNUM

|________| **# of days per week (1-7)**

1b. On average, how many drinks do you have on a day you drink? ALCDRNUM NUMFRQDR.

- ◯ 1. 1-2
- ◯ 2. 3-4
- ◯ 3. 5 or more

For Subsequent Visits, Questions 1c – 1g below refer to events that occurred since your last scheduled study visit)

*1c. Have you engaged in any type of sexual activity while intoxicated? ALCOHSEX FREQZ.

- ◯ 0. Never
- ◯ 1. Sometimes
- ◯ 2. Frequently
- ◯ 3. All the time

*1d. Have you felt you should cut down on your drinking?............. ALCOHCUT YESNO. ◯ 1. Yes ◯ 0. No

*1e. Have people annoyed you by criticizing your drinking?........... ALCOHCRT YESNO. ◯ 1. Yes ◯ 0. No

*1f. Have you felt guilty about your drinking?................................. ALCOHGLT YESNO. ◯ 1. Yes ◯ 0. No

*1g. Have you had a drink first thing in the morning to steady your nerves or get rid of a hangover?............................. ALCOHFST YESNO. ◯ 1. Yes ◯ 0. No

2. Do you smoke cigarettes? CIGARET YESNO.

- ◯ 1. Yes*
- ◯ 0. No

2a. ***If Yes,** for how many years have you been smoking cigarettes?

CIGARNUM |________| **# of years** CIGARNR ◯ No Response

SUBJECT QUESTIONNAIRE

Subject ID: |__|__|__|-|__|__|__|__|__| SUBJID **Visit Date: |__|__|-|__|__|__|-|__|__|__|__|** VISITDT **Visit #: |____|** VISIT

***For Subsequent Visits, the starred (*) below refer to events that occurred since your last scheduled study visit**

*3. Have you used recreational drugs, such as inhalants, consumables, or injectables?

◯ 1. Yes* DRUG
◯ 0. No -> (***Skip to 4.***) YESNO.

*3a. ***If Yes**, have you used any of the following? ***(Mark all that apply)***

DRUG_A – DRUG_M

☐ a. Marijuana/Cannabis/Bhang
☐ b. Hashish
☐ c. Crack
☐ d. Methamphetamines
☐ e. Glue/Petrol sniffing
☐ f. Cocaine
☐ g. Ecstasy
☐ h. Khat
☐ i. Poppers
☐ j Mushrooms
☐ k. LSD
☐ l. GHB
☐ m. Heroin
☐ z. Other, specify: DRUG_Z DRUG_TXT ____________________

◯ None DRUGNONE

3b. For how many years have you used any of the previous drugs? ***(Answer for Visit 1 only)***

|______| DRUGNUM **# of years**

◯ No Response DRUGNR

*3c. Have you injected drugs?

◯ 1. Yes* DRUGINJ
◯ 0. No YESNONR.
◯ 8. No Response

3c1. ***If Yes**, did you share needles with someone while injecting drugs?

◯ 1. Yes DRUGSHAR
◯ 0. No YESNO.

*4. Have you spent time in a jail or prison? ◯ 1. Yes ◯ 0. No PRISON YESNO.

*5. Have you experienced any of the following due to violence in your home? ***(Mark all that apply)***

☐ a. Physical assault VIOLNH_A – VIOLNH_F
☐ b. Sexual assault
☐ c. Interruption/disruption of healthcare
☐ d. Homelessness/Displacement
☐ e. Loss of employment
☐ f. Destruction of property
☐ z. Other, specify: VIOLNH_Z VIOLHTXT ____________________

◯ **Mark if None**

VIOHNONE

QC/QA: ____________ Data Entry: 1st ____________ 2nd ____________

14

SUBJECT QUESTIONNAIRE

Subject ID: |__|__|__|-|__|__|__|__|__| SUBJID **Visit Date: |__|__|-|__|__|__|-|__|__|__|__|** VISITDT **Visit #: |____|** VISIT

For Subsequent Visits, the starred (*) below refer to events that occurred since your last scheduled study visit

*6. Have you experienced any of the following due to political violence? ***(Mark all that apply)***

- ☐ a. Physical assault VIOLNP_A – VIOLNP_F
- ☐ b. Sexual assault
- ☐ c. Interruption/disruption of healthcare
- ☐ d. Homelessness/Displacement
- ☐ e. Loss of employment
- ☐ f. Destruction of property
- ☐ z. Other, specify: VIOLNP_Z ______ VIOLPTXT ______

◯ **Mark if None** VIOPNONE

*7. Have you been injured or physically harmed by a partner or acquaintance?

◯ 1. Yes HARM
◯ 0. No YESNONR.
◯ 8. No Response

*8. Have you been physically harmed or threatened as a result of the perception of your sexual orientation (i.e. being perceived as being gay or lesbian)?

◯ 1. Yes HARMSXOR
◯ 0. No YESNONR.
◯ 8. No Response

9. Did an adult ever have sex with you or touch your private parts when you were a child? ***(Answer for Visit 1 only)***

◯ 1. Yes SEXCHILD
◯ 0. No YESNONR.
◯ 8. No Response

*10. Have you been pressured or forced into sex when you didn't want it?

◯ 1. Yes SEXFORCE
◯ 0. No YESNONR.
◯ 8. No Response

*11. Have you been afraid of your husband/wife or boyfriend/girlfriend?

◯ 1. Yes AFRAID
◯ 0. No YESNONR.
◯ 8. No Response

QC/QA: ______ **Data Entry: 1st** ______ **2nd** ______

SUBJECT QUESTIONNAIRE

Subject ID: |__|__|__|-|__|__|__|__|__| SUBJID **Visit Date: |__|__|-|__|__|__|-|__|__|__|__|** VISITDT **Visit #: |____|** VISIT

"Now I would like to ask you some questions relating to general medical health and conditions. Again, please answer them to the best of your knowledge, and know that you may refuse any question. Please remember that all your answers will be kept confidential. Thank you."

SECTION D - GENERAL HEALTH & MEDICAL CONDITIONS

1. Comparing your health now to one year ago, how would you assess your health?

   ◯ 1. Improved **HLTHASSE**
   ◯ 2. About the same **HLTHASSE.**
   ◯ 3. Gotten worse

2. How frequently do you come to the clinic for routine follow-up appointments? *(For Subsequent Visits, refer to events that occurred since your last scheduled study visit)*

   ◯ 1. Monthly **HLTHFU**
   ◯ 2. Every other month **MONTHFRQ.**
   ◯ 3. Every three months
   ◯ 4. Every six months
   ◯ 90. Other, specify ______________________ **HLTHFTXT**

3. How many times in the past 6 months have you missed a clinic follow-up appointment?

   HLTHMNUM
   |________| **# of missed appointments *(If None, write "0" in the box and skip to 4.)***

   3a. What are some of the reasons that have caused you to miss a follow-up appointment at the clinic? *(Mark all that apply)*

   HLTHM_A – HLTHM_O

   ☐ a. You didn't have time to go
   ☐ b. You didn't have enough money to pay
   ☐ c. You had transportation problems
   ☐ d. You felt too ill
   ☐ e. The clinic is too far from home
   ☐ f. You were afraid to disclose your HIV status
   ☐ g. Your partner or family didn't want you to go
   ☐ h. You were seeing an alternative healer instead
   ☐ i. You were concerned about Stigma or discrimination
   ☐ j. You were feeling better
   ☐ k. HIV treatment isn't important to you
   ☐ l. Clinic staff are disrespectful or uncaring
   ☐ m. Waiting times at the clinic are too long
   ☐ n. Clinic doesn't provide good care
   ☐ o. Military leave or deployment
   ☐ z. Other, specify **HLTHM_Z** ______ **HLTHMTXT** ______

QC/QA: ____________ Data Entry: 1st ____________ 2nd ____________

16

SUBJECT QUESTIONNAIRE

Subject ID: |__|__|__|-|__|__|__|__|__| SUBJID **Visit Date:** |__|__|-|__|__|__|-|__|__|__|__| VISITDT **Visit #:** |__| VISIT

For Subsequent Visits, Questions 4 through 9 below refer to events that occurred since your last scheduled study visit

*4. How many times have you been told by a healthcare provider that you had gonorrhea, chlamydia, syphilis, trichomonas, genital herpes, or any other STD (excluding HIV)?

STDNUM
|________| **# of times**

*5. Do you have any immediate family (parent, sister, brother, children) that have experienced...? ***(Mark all that apply)***

FAMMHX_A - FAMMHX_E

- ☐ a. Cancer
- ☐ b. Diabetes
- ☐ c. Tuberculosis
- ☐ d. Heart Disease (heart attacks)
- ☐ e. Stroke
- ◯ **None** FMHXNONE

*6. Have you had a head injury (or multiple) with...? ***(Mark all that apply)***

- ☐ a. Loss of consciousness for less than one hour HDINJ_A - HDINJ_C
- ☐ b. Loss of consciousness for more than one hour
- ☐ c. Prolonged problems with memory and thinking for over weeks or more
- ◯ **None** HDINNONE

*7. Have you been diagnosed with Tuberculosis (TB)? ◯ 1. Yes ◯ 0. No TBDX YESNO.

*8. Have you used traditional healers (persons) for certain ailments or HIV?

- ◯ 0. Do not use traditional healers
- ◯ 1. Yes, but only for HIV HEALERS
- ◯ 2. Yes, but only for other ailments HEALERS.
- ◯ 3. Yes, for both HIV and other ailments

*9. Have you used traditional remedies for certain ailments or HIV?

- ◯ 0. Do not use traditional remedies
- ◯ 1. Yes, but only for HIV REMEDIES
- ◯ 2. Yes, but only for other ailments REMEDIES.
- ◯ 3. Yes, for both HIV and other ailments

10. ***For Males Only:*** Have you been circumcised?

- ◯ 1. Yes CIRCUMCI
- ◯ 0. No YESNO.

11. How many days in the past 30 days have you been unable to work or perform your daily activities due to poor health?

UNABLNUM
|________| **# of days (0-30)**

QC/QA: ______________ Data Entry: 1st ______________ 2nd ______________

17

SUBJECT QUESTIONNAIRE

Subject ID: |__|__|__|-|__|__|__|__|__| SUBJID **Visit Date:** |__|__|-|__|__|__|-|__|__|__|__| VISITDT **Visit #:** |____| VISIT

12. Do you sleep under a mosquito net? MOSQUITO NSAZ.

◯ 0. Not at all ◯ 1. Sometimes ◯ 2. Always

13. In the last 6 months, have you had a fever? FEVER YESNO.

◯ 1. Yes* ◯ 0. No

13a. ***If Yes,** how many times did you have a fever in the last six months?
FEVERNUM
|________| **# of times**

14. In the last 6 months, have you been diagnosed with Malaria? MALARIA YESNO.

◯ 1. Yes ◯ 0. No -> ***(Skip to 15.)***

14a. How many times have you been diagnosed with Malaria in the last six months?
MALANUM
|________| **# of times diagnosed**

14b. Did you have severe Malaria? ◯ 1. Yes* ◯ 0. No MALASEV YESNO.

14b1. ***If Yes, (Mark all that apply)***
☐ a. Cerebral Malaria MALASV_A
☐ b. Anemia requiring transfusion MALASV_B

14c. In the last 6 months, have you taken medication to treat Malaria?

◯ 1. Yes* ◯ 0. No MALATX YESNO.

14c1. ***If Yes,** how many times have you taken medication to treat Malaria in the last 6 months? MALTXNUM
|________| **# of times taken**

15. In the last 6 months, has anyone in your household been diagnosed with Malaria? How many times?
(Mark all that apply) <u># of times diagnosed</u>

MALAHH_A – MALAHH_I

MALHANUM – MALHINUM

☐ a. Spouse/partner.............................. |________|
☐ b. Parent (yours or spouse's/partner's).............................. |________|
☐ c. Brother or sister.............................. |________|
☐ d. Sons or daughters.............................. |________|
☐ e. Grandparent (yours or spouse's/partner's).............................. |________|
☐ f. Extended adult family member (cousin, uncle/aunt, etc.).............................. |________|
☐ g. Extended adolescent family member (niece, nephew, etc.).............................. |________|
☐ h. Friend.............................. |________|
☐ i. Roommate.............................. |________|

MALAHH_Z
☐ z. Other, specify ______________ MALAHTXT |________| MALHZNUM

◯ **None** MALANONE

END OF SECTION D - GENERAL HEALTH & MEDICAL CONDITIONS 18

SUBJECT QUESTIONNAIRE

Subject ID: |__|__|__|-|__|__|__|__|__| SUBJID Visit Date: |__|__|-|__|__|__|-|__|__|__|__| VISITDT Visit #: |__| VISIT

SECTION E - COGNITION

1. Do you have problems with your memory? MEMORY

   ◯ 1. Yes YESNO.
   ◯ 0. No

2. Have you had difficulty remembering things such as appointments or meetings?

   ◯ 1. Yes MEMORMTG
   ◯ 0. No YESNO.

3. Have you had problems with misplaced objects?

   ◯ 1. Yes MEMOROBJ
   ◯ 0. No YESNO.

4. Do you have problems with concentration? MEMORCON

   ◯ 1. Yes YESNO.
   ◯ 0. No

5. Have you had problems with speech, such as trouble finding the right word or name for something?

   ◯ 1. Yes MEMORSPC
   ◯ 0. No YESNO.

6. Have you noticed slowness in thinking?

   ◯ 1. Yes THINKSLO
   ◯ 0. No YESNO.

7. In the past week, did you drink alcohol more than usual? ALC_MORE

   ◯ 0. Rarely or none of the time (less than one day) ALC_FRQZ.
   ◯ 1. Some or little of the time (1-2 days)
   ◯ 2. Occasionally or a moderate amount of time (3-4 days)
   ◯ 3. Most or all of the time (5-7 days)

8. Which is your dominant hand? *(Answer for Visit 1 only)*

   ◯ 1. Right DOM_HAND
   ◯ 2. Left DOM_HANZ.
   ◯ 3. Ambidextrous

QC/QA: ______________ Data Entry: 1st ______________ 2nd ______________

19

SUBJECT QUESTIONNAIRE

Subject ID: |__|__|__|-|__|__|__|__|__| SUBJID **Visit Date:** |__|__|-|__|__|__|-|__|__|__|__| VISITDT **Visit #:** |__| VISIT

9. **CES-D**

Using the scale below, please indicate how often you experienced certain feelings in the past ***week***.

(Choose only one response per activity on each row)

CESD.

**Feeling**<br>CESD_A – CESD_T	Rarely or none of the time (less than 1 day)<br>0	Some or little of the time (1-2 days)<br>1	Occasionally or a moderate amount of time (3-4 days)<br>2	Most or all of the time (5-7 days)<br>3	No Response<br>8
A. I was bothered by things that usually don't bother me	◯	◯	◯	◯	◯
B. I did not feel like eating; my appetite was poor	◯	◯	◯	◯	◯
C. I felt that I couldn't shake off a feeling of sadness even with help from my family or friends	◯	◯	◯	◯	◯
D. I felt I was just as good as other people	◯	◯	◯	◯	◯
E. I had trouble keeping my mind on what I was doing	◯	◯	◯	◯	◯
F. I felt depressed	◯	◯	◯	◯	◯
G. I felt that everything I did was an effort	◯	◯	◯	◯	◯
H. I felt hopeful about the future	◯	◯	◯	◯	◯
I. I thought my life had been a failure	◯	◯	◯	◯	◯
J. I felt fearful	◯	◯	◯	◯	◯
K. My sleep was restless	◯	◯	◯	◯	◯
L. I was happy	◯	◯	◯	◯	◯
M. I talked less than usual	◯	◯	◯	◯	◯
N. I felt lonely	◯	◯	◯	◯	◯
O. People were unfriendly	◯	◯	◯	◯	◯
P. I enjoyed life	◯	◯	◯	◯	◯
Q. I had crying spells	◯	◯	◯	◯	◯
R. I felt sad	◯	◯	◯	◯	◯
S. I felt that people dislike me	◯	◯	◯	◯	◯
T. I could not "get going"	◯	◯	◯	◯	◯

END OF SECTION E - COGNITION

END OF QUESTIONNAIRE. THANK YOU FOR YOUR TIME.

Form Completed by: FORMBY SIGNED. ______________________ Date: |__|__|-|__|__|__|-|20|__|__| FORMDT

QC/QA: ____________ Data Entry: 1st ____________ 2nd ____________

RV329 - AFRICOS SUBJQ

Version History

Changes made in version 1.2

SUBJQ_B:
Q3 – added format FIELD_DA for the combo box field

SUBJQ_A:
Q3, Q4 – added Q3_COMMENTS, Q4_COMMENTS

SUBJQ_C:
Q11 – Changed format from YESNONR to YNONRNA for added NA option
